# Tandem Reactivity of Metal−Carbon and Carbon−Silicon Bonds in Mononuclear α‐Silyl Organolithium or Organosodium Complexes Towards CO, CO_2_ and Heteroallenes

**DOI:** 10.1002/anie.8906317

**Published:** 2026-04-11

**Authors:** Xiao Yang, Jack M. Hemingway, Wataru Kanna, Nathan Davison, Hiroki Hayashi, Louise Male, Paul G. Waddell, James A. Dawson, Erli Lu

**Affiliations:** ^1^ School of Chemistry University of Birmingham Birmingham UK; ^2^ Chemistry–School of Natural and Environmental Sciences Newcastle University Newcastle upon Tyne UK; ^3^ Institute For Chemical Reaction Design and Discovery (WPI‐ICReDD) Hokkaido University Sapporo Hokkaido Japan; ^4^ Birmingham Centre for Mechanochemistry and Mechanical Processing University of Birmingham Birmingham UK

**Keywords:** CO, CO_2_, C─Si bond, heteroallenes, organolithium, organosodium, silyl migration

## Abstract

α‐Silyl alkyl complexes, such as the ubiquitous MCH_2_SiMe_3_, are some of the most used organometallic building blocks and reagents. Hitherto, most reports focus on the reactive metal–carbon bond, while the carbon–silicon bond has thus far been limited to a handful of serendipitous reaction patterns. Herein, by examining a range of reactions between mononuclear Li or Na α‐silyl alkyl complexes and CO, CO_2_, or heteroallenes (isocyanates, isothiocyanates, carbodiimides), we report tandem M─C and C─Si bond reactivity, with in‐depth mechanistic understanding unveiled by computational reaction pathway studies enabled by the artificial force induced reaction (AFIR) method. By bringing the long‐neglected C─Si bond reactivity to the awareness of synthetic chemists, we demonstrate that the C─Si bond should be taken into account in reaction design when using these α‐silyl alkyl complexes.

## Graphical abstract

Anionic α‐silyl alkyl is one of the most used organometallic building blocks and funtional groups. Traditionally, the C─Si bond in these complexes is considered as inert. In this work, we report a series of reactions between mononuclear organo‐lithium and ‐sodium complexes with CO, CO_2_, or heteroallenes. Both M─C and C─Si bonds take part into the reactions.

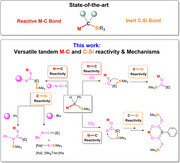

## 1 Introduction

α‐Silyl alkyls, such as ─CH_2_SiMe_3_ and ─CH(SiMe_3_)_2_, were introduced to organometallic chemistry by the late Michael Lappert in the 1970s [1, 2, 3, 4, 5, 6, 7]. Since then, they have developed into one of the most popular functional groups in organometallic chemistry [8]. One major motivation for introducing the α‐silyl substitution, especially trimethylsilyl (‐SiMe_3_), was to suppress *β*‐hydrogen elimination in organometallic complexes (e.g., ─CH_2_CH_3_ vs ─CH_2_SiMe_3_), which was a major cause of their poor thermal stability [9, 10, 11, 12]. Moreover, compared to their carbon analogues (e.g., ─CH_2_SiMe_3_ vs ─CH_2_ *t*Bu), the carbanion is stabilised by the α‐silyl group through so‐called α‐silicon effect, which occurs primarily through negative hyperconjugation: the electron‐rich carbanion donates part of its electron density to the low‐lying σ* orbital of the C─Si bond hence providing stabilisation [13, 14].

**FIGURE 1:**
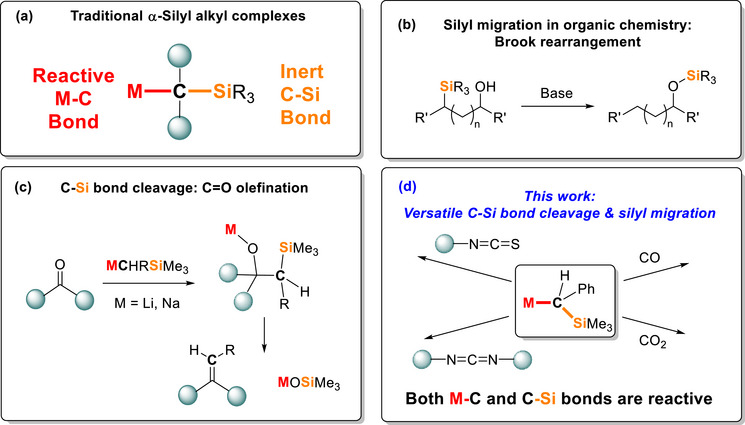
(a) Traditional α‐silyl alkyl complexes: reactive M─C bond, inert C─Si bond; (b) silyl migration in organic chemistry is common, exemplified by Brook rearrangement; (c) the C─Si bond in α‐silyl alkyl complexes can be reactive and unlock new reactivity, exemplified by our recent observations of C═O bond olefination; (d) this work: exploit the reactive C─Si bond in group‐1 α‐silyl alkyl complexes.

Over recent decades, α‐silyl alkyl organometallics MCHRSiR’_3_ (R, R’ = alkyls or aryls), such as the most common MCH_2_SiMe_3_, have been synthesised for most metals across the periodic table. The M─C bond is the major reactive site. For s‐ [15], p‐ [16, 17, 18, 19, 20], f‐ [21, 22, 23, 24, 25] and early d‐block [26, 27, 28, 29, 30] metals, the M─C bond mostly exhibits nucleophilicity and Brønsted basicity, while for mid‐ and late d‐block metals, homolysis of the M─C bond was reported [31]. In most cases, the C─Si bond in the α‐silyl alkyls remains unchanged (Figure 1a).

However, the C─Si bond could be, in principle, reactive. C─Si cleavage in general has been well documented since the 1960s [32, 33, 34] exemplified by the classic Brook rearrangement in organic synthesis (Figure 1b) [35]. However, in the specific area of α‐silyl alkyls organometallic complexes, despite discrete reports over the decades [36, 37, 38], C─Si bond cleavage and resultant silyl migrations of α‐silyl organometallic complexes remain serendipitous findings, instead of a prevalent factor which needs to be taken into account in reaction design.

Nevertheless, such C─Si bond reactivity could have a pronounced influence on the reaction outcome and potentially unlock new reaction patterns. This was exemplified by our recent observations of Li/Na α‐silyl alkyl complexes mediated C═O olefination [39, 40, 41]. It was widely reported that the C═O bond in ketones and aldehydes reacts with the M─C bond in M─CHR(SiMe_3_) complexes of group‐1 [42], group‐2 [43], group‐3 [44, 45, 46, 47, 48], group‐4 [49] and group 13 [50] metals via nucleophilic addition, producing metal alkoxide complexes M─O[C](CHRSiMe_3_) or their hydrolysed product alcohols HO[C](CHRSiMe_3_) ([C]: the substituents on the ketone/aldehyde substrates). In sharp contrast, we observed that, in some cases (particularly for organosodiums), after the initial nucleophilic addition, the C─Si bond was cleaved, eliminating MOSiMe_3_ and generating the corresponding olefins, that is, C═O olefination in a single step, unlike the stepwise protocol generally required for Petersen olefination (Figure 1c) [37, 38, 39].

Given the ubiquitous applications of α‐silyl alkyl organometallics, a systematic understanding of the potential C─Si bond cleavage and silyl migration is essential yet still out of reach. This is particularly important for group‐1 organometallics, which are not only investigated for their own reactivity, but also as building blocks for other organometallics via salt elimination reactions.

In this work, by investigating reactions between mononuclear Li/Na α‐silyl alkyl complexes and a range of molecules with C═E unsaturated bonds (CO, CO_2_, R─N═C═O, R─N═C═S, R─N═C═N─R), we provide a systematic investigation into the behaviour of α‐silyl‐groups (C─Si bond cleavage, silyl migration) in alkali metal (M = Li/Na) organometallic complexes (Figure 1d). Importantly, by isolating and characterising organometallic products resulting from diverse behaviours of the α‐silyl group (1,2‐ and 1,3‐silyl migration), we were able to undertake comprehensive computational studies to elucidate the underlying reaction mechanisms, validating the C─Si bond cleavage processes involved. Noteworthily, new reaction patterns of CO and CO_2_ activations involving C─Si bond cleavage are observed. Our study provides a systematic experimental and computational understanding of C─Si bond reactivity of the α‐silyl alkyl organometallic complexes.

## 2 Results and Discussion

### 2.1 Choices of the Group‐1 α‐silyl Alkyl Complexes Platform

**FIGURE 2:**
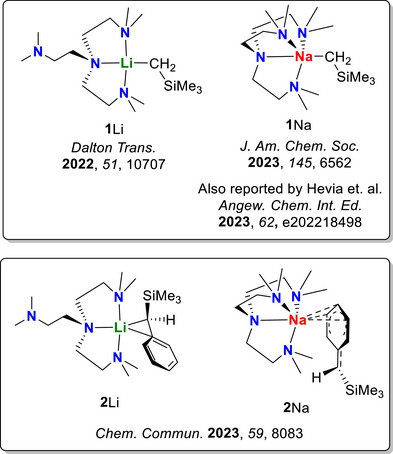
The mononuclear group‐1 α‐silyl alkyl complexes in this study.

Group‐1 organometallics are well known for assembling into clusters—such assemblies profoundly influence their reactivity by introducing multimetallic synergistic effects [51, 52, 53]. To eliminate such multimetallic effects and focus on reactivity of the M─C and C─Si bonds themselves, we utilise the previously reported mononuclear organolithium and organosodium complexes as the platform for this work: [Li(CH_2_SiMe_3_)(Me_6_Tren)] (**1**Li) [54], [Na(CH_2_SiMe_3_)(Me_6_Tren)] (**1**Na) [39, 55], [Li(CHPhSiMe_3_)(Me_6_Tren)] (**2**Li) [38] and [Na(CHPhSiMe_3_)(Me_6_Tren)] (**2**Na) [38]. **1**Li/Na and **2**Li/Na differ from their steric bulkiness on the α‐carbon, i.e., ─CH_2_SiMe_3_ vs ─CHPhSiMe_3_. It should be noted that coordination mode of **2**Na is different from the rest of the group (Figure 2).

We first tested the reactions of **1**Li/Na towards CO and CO_2_, which produced intractable mixtures. We rationalised that this unsatisfactory product stability could be due to the insufficient steric protection of the ‐CH_2_SiMe_3_ group. Hence, we turned to **2**Li/Na, which feature bulkier silyl benzyl group, ─CHPhSiMe_3_.

### 2.2 Reactions Between 2Li/Na and CO—Experimental Observations

As a C1 feedstock, CO has been reported to react with several s‐ and p‐block metal complexes [56], the vast majority from group‐2 and p‐block metal complexes. A small collection of reactions between group‐1 metal complexes and CO were documented as early as in the 1970s, initially with organolithiums [35, 57, 58, 59, 60, 61, 62, 63, 64, 65], more recently with benzyl potassium [66], Li/K amides [67, 68] and a potassium phosphide [69]. According to the literature, the first step is the CO insertion into the polarised AM─E bond (AM: alkali metal; E: C, N, P), which can follow two pathways: (i) 1,1‐insertion to produce an acyl intermediate; (ii) 1,2‐insertion to produce a carbene intermediate. The acyl or carbene intermediates then undergo a range of rearrangements and/or aggregates into isolated products.

**FIGURE 3:**
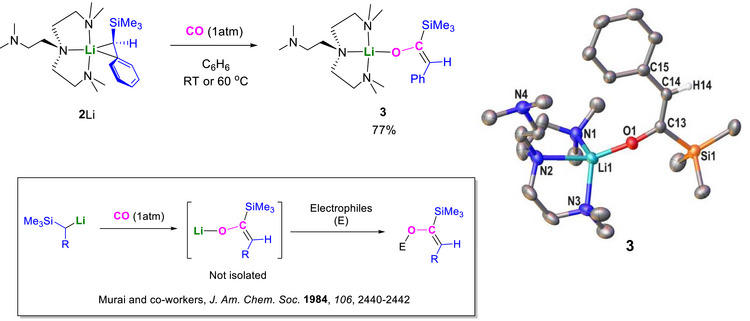
Reaction between **2**Li and CO and the crystal structure of **3** with ellipsoids drawn at the 50% probability level. With the exception of H14, which highlights the hybridisation at C14, hydrogen atoms have been omitted for clarity.

The reaction between **2**Li and CO (1 atm) was first monitored at NMR‐scale in d_6_‐benzene, followed by a scaling up reaction in benzene at room temperature or 60°C, affording **3** in a 77% isolated yield as yellow crystals. Single‐crystal x‐ray diffraction study of **3** reveals its α‐silyl enolate structure (Figure 3), which matches its NMR spectra (see Supporting Information). The α‐silyl enolate structure of **3** matches with the hypothesis from Murai and co‐workers in 1984 [35], where the lithium silyl enolate was not isolated but quenched with electrophiles.

**FIGURE 4:**
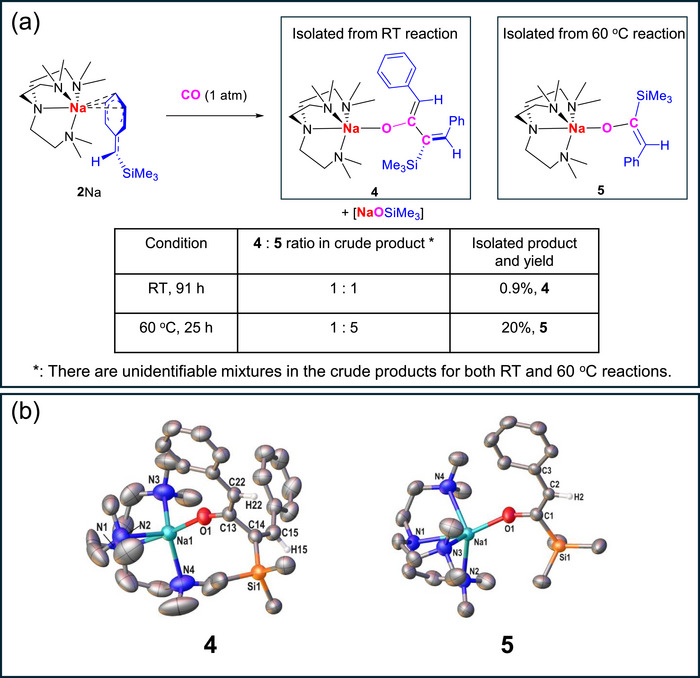
(a) Reactions between CO and **2**Na at room temperature and 60°C; (b) the crystal structures of **4** and **5** with ellipsoids drawn at the 50% probability level. In **5**, the carbons in Me_6_Tren are disordered over two positions, with only the major component shown for clarity. With the exception of those that show the presence of the double bond on the enolate, hydrogen atoms, as well as solvent molecules, have been omitted for clarity.

We then investigated the reactions between **2**Na and 1 atm of CO at room temperature, as well as 60°C. Interestingly, two structurally distinct products, **4** and **5**, were obtained, and their molecular structures can be found in Figure 4b. Both **4** and **5** are produced at room temperature or 60°C, but in different ratios (Figure 4a). At room temperature, the **4**:**5** ratio is approximately 1:1 in the crude product, among other intractable mixtures. When the temperature is elevated to 60°C, the reaction is cleaner and the **4**:**5** ratio in the crude product is approximately 1:5. Fractional crystallisation of the crude products yielded **4** (0.9% yield) and **5** (20% yield) from the room temperature and 60°C reactions, respectively (Figure 4a). It should be noted that the crystalline yields, especially for **4**, are low but reproducible.

While **5** is an analogue of **3**, **4** represents a new reaction pattern for CO activation. Formally, two CO molecules are consumed in the formation of **4**, as indicated in Figure 4a by the magenta C and O atoms. We hypothesise that the other O atom of the two CO molecules is incorporated in the [NaOSiMe_3_]_n_, which is observed in the NMR monitoring experiment and matches with our previous report [39]. We carried out computational reaction pathway studies to understand the mechanisms, and hopefully shed light on the distinct behaviors between **2**Li and **2**Na.

### 2.3 Reactions Between 2Li/Na and CO—Reaction Mechanisms

**FIGURE 5:**
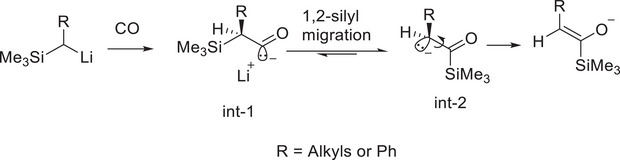
Hypothesised mechanism to produce enolate from α‐silyl organolithium and CO reaction by Murai and co‐workers [35].

The formation of **3**, **4** and **5** presents a challenge of our mechanistic understanding of these reactions. This is particularly important for **5**, which represents a new CO activation pattern. For **3** and **4**, though it was hypothesised as early as in 1984 that an α‐silyl enolate could be formed from a reaction between α‐silyl organolithiums and CO (Figure 3 Murai and co‐workers [35]), the detailed mechanism remains unclear. Murai and co‐workers postulated that the CO undergoes a 1,1‐insertion into the Li─C bond to form an acyl intermediate (Figure 5 int‐1), which then undergoes a 1,2‐silyl migration to form the α‐carbonyl carbon anion intermediate (Figure 5 int‐2), followed by the enolisation. But this postulated mechanism was not confirmed by state‐of‐the‐art computational reaction pathway studies. Moreover, the alkali metal identity, that is, Li vs Na, obviously plays a decisive role in the CO activation reactions. To answer these questions, in‐depth computational reaction pathway investigations are needed.

Given the status quo, we conducted computational reaction pathway studies for the reactions between **2**Li/**2**Na and CO. To explore the reaction in a robust manner, we utilised the artificial force induced reaction (AFIR) method [70, 71, 72, 73, 74], which has proved its vast capability in predicting new and validating experimental reaction pathways and intermediates [75, 76, 77, 78, 79]. The AFIR method was used to explore the force‐modulated potential energy surface (PES). Rather than providing a transition state guess, the AFIR method instead requires a selection of atoms between which an external force is applied that carries the structure over the barrier on the PES returning an approximate transition state structure thus avoiding unintentional biasing of the calculations towards a preconceived outcome. The force‐modulated potential energy surface was then relaxed using the locally‐updated planes (LUP) method which gives an improved PES which more closely aligns with the true PES, also yielding an improved transition state approximation. Further geometric relaxation of the approximate transition state (to a first order saddle point) is conducted using the methodology outlined in the Supporting Information. This process was repeated for every step of each reaction considered in this work.

**FIGURE 6:**
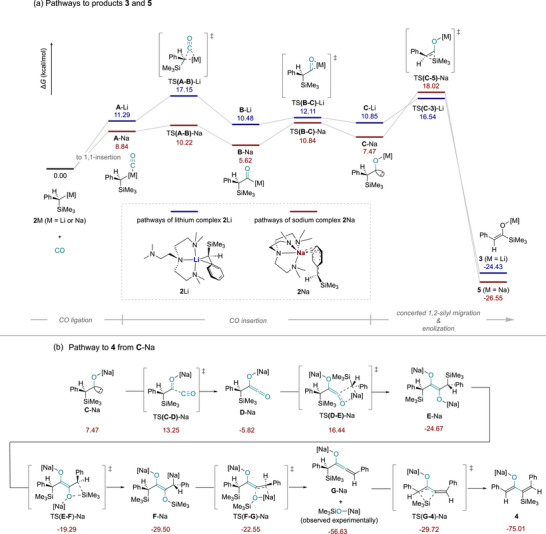
(a) Calculated reaction pathways to produce the enolates, structures **3** and **5**; (b) schematic reaction pathway outlining the reaction pathway to product **4**.

Using the AFIR method on the reactions between CO and **2**Li/**2**Na to generate the reaction pathways therefore allowed us to interrogate the differences in reactivity. The calculated reaction pathways towards the enolate products (**3** and **5**) are exhibited in Figure 6a. It is obvious that, compared to the rather simple hypothesis in Figure 5, the calculated scenario is more complicated. Indeed, the 1,1‐insertion acyl intermediate is located as **B**‐Li/Na in Figure 6a, but instead of a direct silyl migration to form the enolate, it undergoes a C‐to‐O metal migration to form the carbene intermediate (Figure 6a **C**‐Li/Na). The silyl migration takes place from the carbene intermediate to form the isolated enolate products. It is worth noting that our calculations are based on un‐truncated models, that is, all the steric factors are considered, including the potential ligand coordination dynamics.

Through consideration of Figure 6a, it is clear to see that the two reaction pathways for Li and Na are similar, with the most noticeable difference being that each step of the reaction is lower in energy for the Na system relative to its starting material than that of the Li system, with the exception of the silyl migration TS (TS(**C**‐**3**/**5**)‐M). Both reactions were found to progress through an intermediary 5/6‐coordinated alkali metal structure (for Li/Na, respectively) that mediated the insertion (**A**‐Li/Na). The insertion step, that being the step going from **A**‐Li/Na to **B**‐Li/Na via TS(**A**‐**B**)‐Li/Na, is a lower barrier for the Na system than the Li system potentially due to the longer Na bonding distances than those of Li allowing for less of a barrier to insertion. It is clear that the barrier to silyl migration (that of TS(**C**‐**3**/**5**)‐M) is lower for Li than it is for Na, 5.70 versus 9.90 kcalmol^−1^. This alone does not offer an explanation for the difference in observed reactivities outlined previously but does suggest that **C**‐Na may persist in the reaction mixture longer than **C**‐Li, as such the reaction pathway for the formation of **4** was explored to see if further insight could be gained.

The reaction to form **4** was thought to proceed through the same initial steps as the previously outlined reaction to form **5**, up to **C‐**Na (the carbene intermediate), at which point a different route of reaction would occur to yield **4** in the case of Na, but not in the case of Li (schematic diagram for the subsequent steps to yield **4** are shown in Figure 6b). In this case, **C**‐Na further reacts with an additional molecule of CO to yield a ketene intermediate, **D**‐Na, which undergoes a series of further reaction steps to yield product **4**. Gratifyingly, the computed reaction pathway also results in the formation of [Na]OSiMe_3_ as was discussed previously and corroborated by experiment.

**FIGURE 7:**
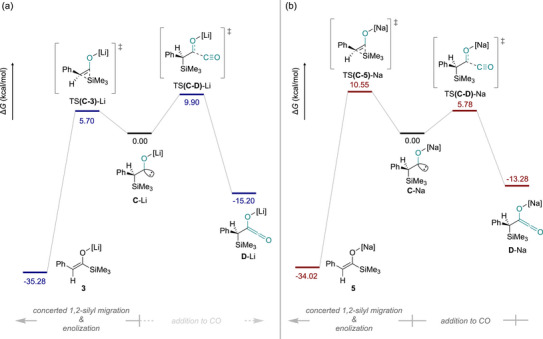
Comparison of calculated reaction pathways of **C**‐M (where M = Li (a) and M = Na (b)) for the silyl migration to form the enolate and reaction with additional CO to form the ketene intermediate. All energies are given in kcalmol^−1^ and have been normalised to the value of **C**‐M in each respective case.

To explore the origin of the difference in reactivity for the two alkali metal species, a comparison between the silyl migration step and the reaction with an additional CO molecule (both stemming from **C**‐M) was completed and is shown in Figure 7.

**FIGURE 8:**
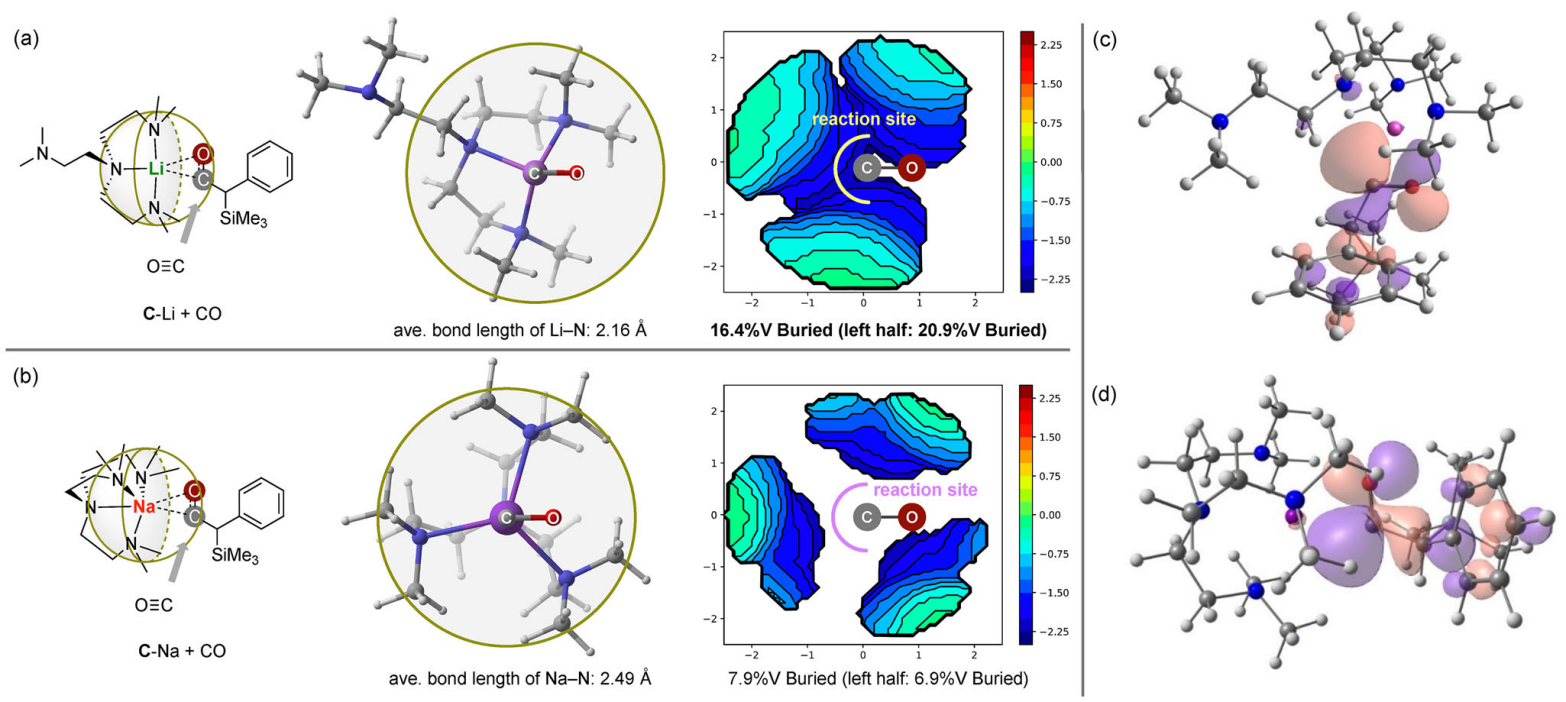
Highlights the steric maps of **C**‐Li/Na (a and b, respectively) as well as the HOMO orbitals (c and d, respectively).

From Figure 7, it is clear that the most favourable reaction from **C**‐Li is that of silyl migration to form product **3**, being 4.20 kcalmol^−1^ lower in energy than the reaction with another molecule of CO. In contrast, for **C**‐Na, the reaction with an additional CO molecule is lower in energy than that of the silyl migration, suggesting that reaction with CO is more plausible. One possible reason for this is the increased Na─N^Tren^ bond length (2.49 Å ave.) compared to the Li─N^Tren^ bond lengths (2.16 Å ave.) resulting in a less sterically congested carbene centre for the CO to interact with. This hypothesis is supported using topographical steric maps (shown in Figure 8) which show that the buried volume is significantly larger in the Li system (16.4%) when compared to the Na system (7.9%) with this effect being even more pronounced at the reactive carbene carbon (20.9% vs. 6.9% for Li and Na, respectively). Moreover, consideration of the HOMOs of **C**‐Li/Na (also shown in Figure 8) reveals that they are centred on the carbene carbon atom with the energy of **C**‐Na being larger than **C**‐Li (−3.42 vs. −3.68 eV) suggesting a higher degree of nucleophilicity. Overall, the decrease in steric crowding and relative increase in nucleophilicity of the carbene carbon suggest that **C**‐Na is more likely to react with CO than **C**‐Li, giving rise to the lower barrier relative to the silyl migration.

It should be noted that an increased amount of **5** is formed relative to **4** at higher temperature, we suggest that this is a kinetic effect whereby a larger proportion of **C**‐Na molecules have sufficient energy to overcome the barrier to silyl migration prior to reacting with additional CO. This hypothesis was strengthened by an observed reduction in formation of **5** at low temperature (−20°C) (for details see Figure S19).

### 2.4 Reactions Between 2Li/Na and CO_2_

Several examples of reactions between CO_2_ and AM─E bonds (AM: alkali metal; E: C, N) have been reported, but the reaction patterns are rather limited. The prevalent reaction pattern is 1,2‐insertion of the C═O bond(s) into the AM─E bond, forming carboxylates or *bis*‐metalated diols [80, 81, 82, 83, 84]. In most of the examples, there was no further rearrangement observed. The only exception was reported by Mulvey and co‐workers [85], where a Li–Na carbamato‐anhydride was isolated as the product, along with a *bis*‐trimethylsilyl ether, but the mechanism was not discussed therein. Expanding the reaction patterns to a range of alkali metal complex‐mediated CO_2_ activation reactions is highly desirable to broaden our fundamental understanding of CO_2_‐based chemical conversions.

**FIGURE 9:**
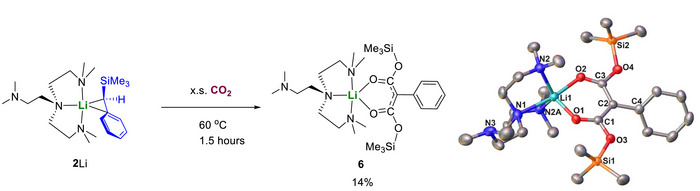
Reaction between **2**Li and CO_2_ to produce **6** and its crystal structure with ellipsoids drawn at the 50% probability level and hydrogen atoms omitted for clarity. In **6**, part of Me_6_Tren is disordered over two positions, with only the major component shown for clarity. Hydrogen atoms have been omitted for clarity.

We tested reactions between **2**Li/Na and CO_2_. While **2**Na and CO_2_ reacted rapidly at room temperature yet produced an intractable mixture, the reaction between **2**Li and CO_2_ produced a clean product **6** after being heated at 60°C for 18 h, along with unidentifiable white solids. Further heating the product at 60°C in *d_6_*‐benzene for 8 h leads to only negligible decomposition (Figure S20). The reaction was scaled up in benzene at 60°C and complex **6** was isolated as colorless crystals in 14% crystalline yield. It should be noted that the low crystalline yield is a result of the poor solubility of **6** and the resultant difficulty in crystallisation. Indeed, **6** is the major soluble product as indicated by the ^1^H NMR spectrum of the crude product (Figure S22). Single crystals of **6** were obtained from *n*‐hexane solution and its crystal structure is shown in Figure 9.

**FIGURE 10:**
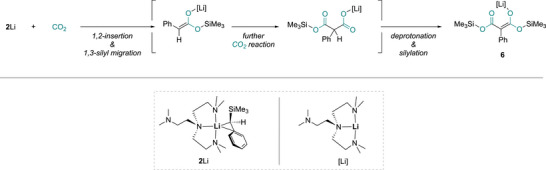
Postulated reaction mechanism to form **6**.

**6** is a silyloxo‐substituted acetylacetonate (*acac*) complex, with no proton on C2. The formation of **6** is different from all the reported reaction patterns between organo‐alkali metal reagents and CO_2_. We postulate that **6** is formed via a 2:2 reaction between **2**Li and CO_2_, initiated by 1,2‐insertion of one of the two C═O bonds of CO_2_ into Li─C bond, followed by 1,3‐silyl migration, then the intermediate further reacts with the second molecule of CO_2_, followed by further reaction with another molecule of **2**Li leading to deprotonation and silylation (or vice versa) to produce the final product **6** (Figure 10).

### 2.5 Reactions Between 2Li/Na and Isocyanates/Isothiocyanates

Following CO_2_, we investigate reactions between **2**Li/Na and more accumulated E═C═E heteroallene substrates, namely isocyanate, isothiocyanate and carbodiimide.

**FIGURE 11:**
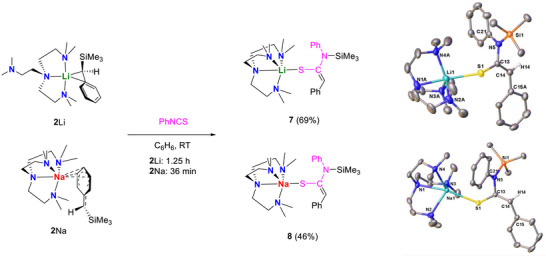
Synthesis and crystal structures of **7** and **8** with ellipsoids drawn at the 50% probability level. In **7**, the Me_6_tren and phenyl groups are disordered over two positions, with only the major component shown for clarity. Solvent molecules and hydrogen atoms have been omitted for clarity, except for H14, which highlights the hybridisation at C14.

Phenyl isocyanate (PhNCO) was mixed with **2**Li/Na at room temperature in benzene. The reactions were fast, leading to immediate consumption of the starting materials, however an intractable mixture was produced in both cases. Gratifyingly, switching from O to S, that is, changing from PhNCO to PhNCS, yielded clean products, **7** and **8**, in the reactions with **2**Li and **2**Na, respectively (Figure 11). **7** and **8** were isolated as yellow crystalline solids in 69% and 46% yield, respectively. Their crystal structures (Figure 11) share similar structural features, hence, will be discussed together.

**FIGURE 12:**
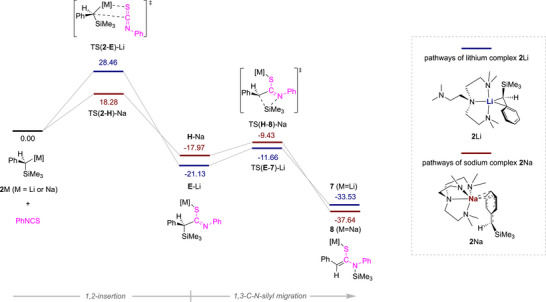
Reaction pathways for the formation of **7** & **8** from **2**Li/Na and PhNCS.

In the structures of **7** and **8**, the C13─C14 bond lengths are 1.3537(18) and 1.358(2) Å, respectively, in the range of a typical C═C double bond. The C13─N5 bond lengths, 1.4608(15) and 1.465(2) Å, indicate C─N single bonds. Based on the structures of **7** and **8**, we hypothesise that their formation is a result of an initial 1,2‐insertion of the S═C bond into the M─C bond followed by a 1,3‐C─N silyl migration, which is corroborated by calculations, reaction pathways shown in Figure 12.

The calculated pathways for both reactions follow the same mechanism with the key difference being the barrier to the initial insertion of S═C into the M─C bond. We hypothesise that the increased barrier to insertion provided in the case of **2**Li is as a result of a more sterically shielded reactive carbanion attributable to the smaller ionic radius of Li^+^ cf. Na^+^. While a barrier of circa 28.5 kcal per mol may appear high (TS(2‐E)‐Li), the coordination mode of the Me_6_Tren ligand is flexible in solution which allows for increased likelihood of insertion [86].

**FIGURE 13:**
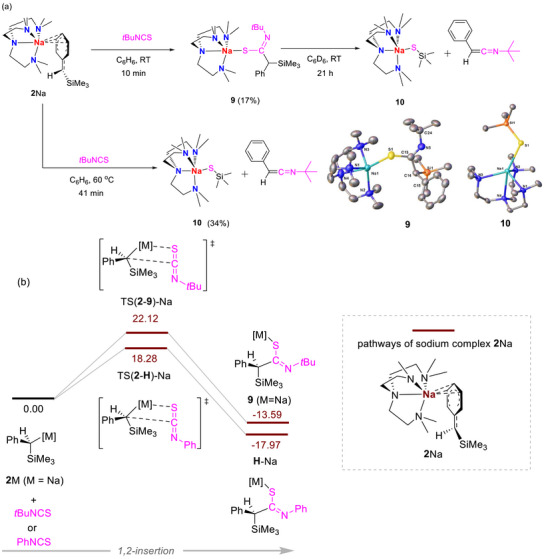
(a) Reactions between **2**Na and *t*BuNCS, and the crystal structure of **9** and **10** with ellipsoids drawn at the 50% probability level and hydrogen atoms omitted for clarity. (b) The reaction pathway diagram for the formation of **9** with 1,2‐insertion step for PhNCS shown for comparative purposes.

Interestingly, the substituent of isothiocyanate has a profound effect on the reaction outcome. *Tert*‐butyl isothiocyanate, *t*BuNCS, reacted with **2**Na rapidly at room temperature to produce **9** in a low but reproducible yield (17%) (Figure 13a). It should be noted that the low yield of **9** is a result of its good solubility, instead of the presence of side product(s), which is confirmed by the crude ^1^H NMR spectrum (Figure S40). **9** can be considered as the product of a formal C═S bond insertion into Na─C bond, prior to the silyl migration. A reaction pathway outlining the formal 1,2‐insertion (Figure 13b) outlines this process.

However, heating **9** to 60°C for 41 min (Figures 13a and S45) did not promote the silyl migration but instead, resulted the in an olefination process via silyl elimination, relevant to our reported C═O olefinations [41, 42, 43], to form a sodium trimethylsilanethiolate **10** (isolation yield: 34%) and a *C*‐Ph‐*N*‐*t*Bu‐ketenimine. The ketenimine was not isolated but its presence was confirmed by comparing the ^1^H NMR spectrum of the NMR‐scale reaction with previous reports [87, 88, 89, 90, 91]. These products were also observed in the room‐temperature NMR scale reaction but at longer time (Figures S39 and S45).

### 2.6 Reactions Between 2Li/Na and Carbodiimides

With the observation of the different C–Si reactivity induced by the *t*Bu group in *t*BuNCS, we next explored the reaction between **2**Li/Na and *t*Bu‐N═C═N‐*t*Bu, wondering how the two *t*Bu groups would influence the reaction outcome. Insertion of C═N bond of carbodiimides into a polarised M─C bond is a well‐established method to synthesise the widely used amidinate ligands [92, 93]. α‐Silyl alkyl complexes were extensively investigated in this reaction, including LiCH_2_SiMe_3_ [94]. While the C═N insertion is facile even for the sterically congested cases [95], silyl migration is scarce.

**FIGURE 14:**
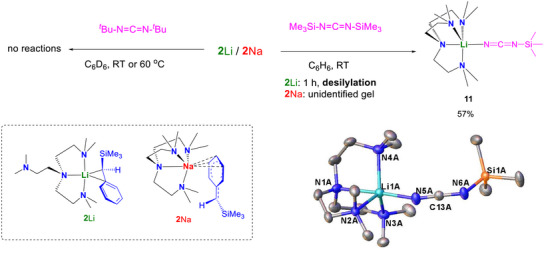
Reactions between **2**Li/Na and *t*Bu‐N═C═N‐*t*Bu or Me_3_Si‐N═C═N‐SiMe_3_, and crystal structure of **11** with ellipsoids drawn at the 50% probability level and hydrogen atoms omitted for clarity. **11** contains two crystallographically‐independent molecules in the asymmetric unit, of which only one is shown.

To our surprise, neither **2**Li nor **2**Na react with *t*Bu‐N═C═N‐*t*Bu at room temperature or 60°C (Figure 14): even the C═N bond insertion does not occur. We attribute this inertness to the enhanced steric congestion of the system, though the detailed mechanism remains unclear. Nevertheless, changing the carbodiimide *t*Bu group into ─SiMe_3_ resulted in unexpected reactivity. Despite the similar steric profile of *t*Bu and ─SiMe_3_. Me_3_Si‐N═C═N‐SiMe_3_ reacted with **2**Li at room temperature, in 1 h, **11** was isolated in 57% yield and its crystal structure is exhibited in Figure 14. **11** is a product of Me_3_Si‐N═C═N‐SiMe_3_ desilylation. Similar desilylation was reported in zirconium complexes [96, 97], but to the best of our knowledge, this is new to group‐1 metal chemistry. Organic product of the desilylation, PhCH(SiMe_3_)_2_, was observed in the NMR scale reaction between **2**Li and Me_3_Si‐N═C═N‐SiMe_3_. Unlike **2**Li, reaction between **2**Na and Me_3_Si‐N═C═N‐SiMe_3_ produced a colourless gel which is insoluble in *d*_6_‐benzene, accompanied by PhCH(SiMe_3_)_2_ (Figure S59 for the ^1^H NMR spectrum). Further work is underway in our group to fully exploit the diversified reactivity of **2**Li and **2**Na towards carbodiimides.

## 3 Conclusion

**FIGURE 15:**
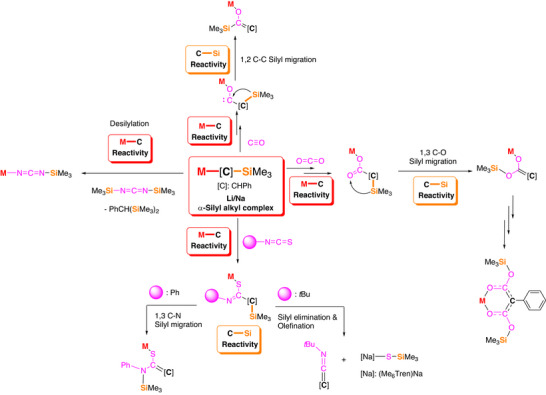
Summarisation of the M─C and C─Si bond reactivity of mononuclear Li/Na α‐silyl benzyl complexes with CO, CO_2_, isothiocynates and carbodiimides.

Through the reactions in this work (summarised in Figure 15), we conclude that C─Si bond in α‐silyl alkyl complexes should be treated as reactive, instead of inert as is convention. This is a particularly important factor if the substrate contains a heteroatom (e.g., N, S, O). We observed here that the reactions are usually initiated by the polarised M─C bond reacting with an electrophilic site (such as C═E bond), following the nucleophilic addition/insertion manner. Subsequently, if there is a site where the silyl can readily migrate to, such as N, O, or carbene, the C─Si bond will cleave and the silyl undergoes 1,2‐ or 1,3‐migration. Longer distance migration was not observed, and intramolecular migration was observed in most cases, except the CO_2_ case where apparently an intermolecular mechanism operates. We attribute the prevalent C─Si bond cleavage and silyl migration to the formation of strong N─Si or O─Si bonds. This is a key point to note for synthetic chemists: when using these group‐1 α‐silyl alkyl complexes with substrates featuring N/O atoms adjacent to the designed electrophilic reactive site, judicious considerations must be taken to control, or to exploit, the reactive C─Si bond.

## Funding

The authors thank the Leverhulme Trust for Research Project Grants (RPG‐2022‐231: J.M.H., J.A.D, N.D., E.L; RPG‐2023‐159: E.L.). X.Y. thanks University Of Birmingham for a PhD studentship. H.H. gratefully acknowledge JSPS‐WPI, JST‐ERATO (JPMJER1903), Transformative Research Areas (A) Green Catalysis Science for Renovating Transformation of Carbon Based Resources (Green Catalysis Science) (24H01830) for research support. The authors thank University of Birmingham, Newcastle University and Hokkaido University for their generous financial support.

## Conflicts of Interest

The authors declare no conflicts of interest.

## Supporting information




**Supporting File 1**: anie72186‐sup‐0001‐SuppMat.docx.

**Supporting File 2**: anie72186‐sup‐0002‐CIF.zip.

## Data Availability

The Supporting Information is available free of charge for additional experimental details, materials, and methods. CCDC 2324926 (**3**), 2324927 (**4**), 2439663 (**5**), 2531967 (**6**), 2533795 (**7**), 2533796 (**8**), 2533797 (**9**), 2539212 (**10**) and 2533798 (**11**) contain the supplementary crystallographic data for this paper. These data can be obtained free of charge via www.ccdc.cam.ac.uk/data_request/cif, or by emailing data_request@ccdc.cam.ac.uk, or by contacting The Cambridge Crystallographic Data Centre, 12 Union Road, Cambridge CB2 1EZ, UK; fax: +44 1223 336033.
