# Supplementary material for: Tandem Reactivity of Metal−Carbon and Carbon−Silicon Bonds in Mononuclear α‐Silyl Organolithium or Organosodium Complexes Towards CO, CO2 and Heteroallenes
Source: Angew Chem Int Ed Engl. 2026 Apr 11;65(21):e8906317. doi: 10.1002/anie.8906317 (PMC13182206; doi:10.1002/anie.8906317)
Supplement: Supplementary file 1 — Supporting File 1: anie72186‐sup‐0001‐SuppMat.docx. [file ANIE-65-e8906317-s002.docx]

Supplementary Materials for

**Tandem Reactivity of Metal−Carbon and Carbon−Silicon Bonds in Mononuclear α-Silyl Organolithium or Organosodium Complexes towards CO, CO_2_ and Heteroallenes**

Xiao Yang,^1^ Jack M. Hemingway,^2^* Wataru Kanna,^3^ Nathan Davison,^1^ Hiroki Hayashi,^3^* Louise Male,^1^ Paul G. Waddell,^2^ James A. Dawson,^2^ Erli Lu ^1,4^*¶

Affiliation:

1. School of Chemistry, University of Birmingham, Birmingham, B15 2TT (UK).

2. Chemistry‒School of Natural and Environmental Sciences, Newcastle University, Newcastle upon Tyne, NE1 7RU (UK).

3. Institute for Chemical Reaction Design and Discovery (WPI-ICReDD), Hokkaido University, Kita 21 Nishi 10, Kita Ku, Sapporo, Hokkaido, Japan

4. Birmingham Centre for Mechanochemistry and Mechanical Processing, University of Birmingham, Birmingham, B15 2TT (UK).

*Corresponding author: e.lu@bham.ac.uk (E. L.); hhayashi@icredd.hokudai.ac.jp (H. H.); jack.hemingway@newcastle.ac.uk (J. M. H.)

¶ Homepage: <https://www.birmingham.ac.uk/staff/profiles/chemistry/lu-erli>

**This PDF file includes:**

Materials and Methods

Supplementary Text

Figures S1 to S62

Tables S1 to S20

Section1.Experimental methods and data------------------------------------------------------------S2

Section2 Computational methods------------------------------------------------------------------- S62

References---------------------------------------------------------------------------------------------S100

**Section 1**

**Materials and Methods**

- 1. General procedures

All manipulations were carried out using Schlenk techniques, in a Vigor^TM^ glovebox equipped with a −35 ˚C freezer and a cold well, or in a MBraun glovebox equipped with a −40 ˚C freezer and a cold well, under an atmosphere of dry argon. Benzene, toluene and *n*-hexane was dried with activated 4Å molecule sieves and kept in the glovebox. Chemicals were purchased from Merck, Fluorochem, TCI, Fisher or Alfa Aesar, and dried under dynamic vacuum for several hours (for solids), or over activated 4Å molecular sieves (for liquids), prior to use. CO gas (N3.7, Research Grade, 99.97%) and CO_2_ were purchased from the BOC and used without further purification.

All glassware, including pipettes, vials and ampoules, must be silylated prior to use by treating with trimethylsilyl chloride (Me_3_SiCl), rinsing with water, and dried in a 150 °C oven for 12 hours. Failing to silylate glassware will lead to significantly reduced yields, and in some cases, irreproducible results.

The organolithium and organosodium complexes in this work are highly reactive towards silicone grease. We would suggest excluding grease as much as possible for all chemicals used herein, including in the solvent distillation apparatus. Otherwise, irreproducible by-products or side products may appear. For this purpose, we used J. Young tap solvent flasks and vacuum transfer apparatus to dry our solvents. [Li(CH_2_SiMe_3_)(Me_6_Tren)] (**1**Li), [Na(CH_2_SiMe_3_)(Me_6_Tren)] (**1**Na), [Li(CHPSiMe_3_)(Me_6_Tren)] (**2**Li) and [Na(CHPhSiMe_3_)(Me_6_Tren)] (**2**Na) and [NaCH_2_SiMe_3_] were prepared as previously reported.^[[1]](#endnote-1)^

^1^H were recorded on a Bruker Avance III 300 or 400 spectrometer operating at 400 or 300 MHz. ^7^Li and ^23^Na NMR spectra were recorded on a Bruker Avance III (or NEO console) 400 spectrometer at 155 and 106 MHz, respectively. HSQC spectra were recorded on a Bruker Avance III HD 700 or NEO console 400 spectrometer operating at 700 or 400 MHz. ^13^C and ^13^C dept NMR spectra were recorded on a Bruker Avance III HD 700 or NEO console 400 spectrometer operating at 176 or 101 MHz respectively. Chemical shifts are quoted in ppm and are relative to benzene (^1^H and ^13^C).

- 1. NMR scale and scale-up reactions

**NMR scale reaction between CO and 2**Li **at room temperature**

**2**Li (0.0120 g, 0.03 mmol) was dissolved in C_6_D_6_ (0.4887 g, 0.5 mL). Then the yellow solution was transferred into an J. Young NMR tube and sealed. The NMR tube was connected to a Schlenk line, degassed by the freeze-thaw-vacuum protocol for three times to remove the argon, and charged with CO (1 atm). The colour of the solution changed from yellow to orange upon contact with the CO at room temperature. The reaction was monitored by ^1^H and ^7^Li NMR spectroscopies (Figure S1 and S2, respectively), indicating a >95% conversion into the product **3** within 22 hours. **3** was further monitored for its thermostability, indicating it is stable in C_6_D_6_ for at least 4 days.

**Figure S1:** ^1^H NMR (C_6_D_6_, 25 ˚C, 400 MHz) of the NMR scale reaction between **2**Li and CO at room temperature.

**Figure S2:** ^7^Li NMR (C_6_D_6_, 25 ˚C, 155 MHz) of the NMR scale reaction between **2**Li and CO at room temperature.

**Scale-up reaction between CO and 2**Li **at room temperature**

At room temperature, **2**Li (0.4006 g, 1 mmol) was dissolved in benzene (3 mL). The yellow solution was transferred into a 50 mL J. Young tap ampoule, degassed by the free-thaw-vacuum protocol for three times, and charged with CO (1 atm). The mixture was stirred for 26 h at room temperature and then removed all the volatiles *in vacuo*. The crude product was obtained as a brownish yellow solid, which was monitored by ^1^H NMR, indicating that the major component is the product **3**. The crude product was washed with *n*-hexane (1 mL × 3), after removing all volatiles, **3** was obtained as a yellow solid in 77% yield (0.3308 g). Single crystals (yellow plates) suitable for the SCXRD study were obtained using a 0.5 mmol crop, dissolved in a mixture of *n*-hexane (0.5 mL, poor solvent) and benzene (0.5 mL, good solvent), after standing at room temperature for a few hours.

**Figure S3:** ^1^H NMR (C_6_D_6_, 25 ˚C, 400 MHz) of **3**.

^1^H NMR (400 MHz, C_6_D_6_, 25 ˚C) δ(ppm) 8.20 (d, ^3^*J*_HH_ = 7.7 Hz, 2H, Ar-*H*), 7.34 (t, ^3^*J*_HH_ = 7.7 Hz, 2H, Ar-*H*), 7.06 (t, ^3^*J*_HH_ = 7.2 Hz, 1H, Ar-*H*), 5.83 (s, 1H, PhC*H*=C), 2.23 (t, ^3^*J*_HH_ = 5.6 Hz, 6H, NC*H_2_*C*H_2_*N), 2.04 (t, ^3^*J*_HH_ = 5.6 Hz, 6H, NC*H_2_*C*H_2_*N), 2.00 (s, 18H, N(C*H_3_*)_2_), 0.46 (s, 9H, Si(C*H_3_*)_3_).

**Figure S4:** ^7^Li NMR (C_6_D_6_, 25 ˚C, 155 MHz) of **3**.

^7^Li NMR (155 MHz, C_6_D_6_, 25 ˚C) δ(ppm) 0.86.

**Figure S5:** ^13^C{^1^H} NMR (176 MHz, C_6_D_6_, 25 ˚C) of **3**.

^13^C{^1^H} NMR (176 MHz, C_6_D_6_, 25 ˚C) δ(ppm) 180.76 (Si*C*(O)=CH), 143.85(Ar*C*), 127.78 125.58, 120.26 (Ar*C*H), 105.46 (Ph*C*H=C), 57.09 (N*C*H_2_*C*H_2_N), 51.39 (N*C*H_2_*C*H_2_N), 45.46 (N(*C*H_3_)_2_), -0.64 (Si(*C*H_3_)_3_).

**Figure S6:** ^13^C DEPT 135 NMR (176 MHz, C_6_D_6_, 25 ˚C) of **3**.

**Figure S7:** HSQC (C_6_D_6_, 25 ˚C, 700/176 MHz) of **3**. Red: (CH, CH_3_) Blue: (CH_2_).

**Figure S8:** UV/Vis spectrum of **3** (0.01 mM in hexane, scan internal: 0.5 nm, scan range: 200-900 nm, Shimadzu UV-1800).

**NMR scale reaction between CO and 2**Li **at 60 ^o^C:**

**2**Li (0.0160 g, 0.04 mmol) was dissolved in C_6_D_6_ (0.4802 g, 0.5 mL). Then the yellow solution was transferred into an J. Young NMR tube and sealed. The NMR tube was connected to a Schlenk line, degassed by the freeze-thaw-vacuum protocol for three times to remove the argon, and charged with CO (1 atm). The colour of the solution changed from yellow to light dark orange once contacted with CO. The reaction was heated to 60 ^o^C monitored by ^1^H and ^7^Li NMR spectroscopies (Figure S9 and S10, respectively), indicating a >95% conversion into the product **3** within 19 hours.

**Figure S9:** ^1^H NMR (C_6_D_6_, 25 ˚C, 400 MHz) of the NMR scale reaction between **2**Li and CO at 60 ^o^C.

**Figure S10:** ^7^Li NMR (C_6_D_6_, 25 ˚C, 155 MHz) of the NMR scale reaction between **2**Li and CO at 60 ^o^C.

**NMR scale reaction between CO and 2**Na **at room temperature**

**2**Na (0.0126 g, 0.03 mmol) was dissolved in C_6_D_6_ (0.4876 g, 0.5 mL) and loaded into a J. young tap NMR tube. The NMR tube was degassed by the freeze-thaw-vacuum protocol three times, then charged with CO. The reaction was monitored by ^1^H NMR spectroscopy (Figure S11). (The ratio of products **4** and **5** was about 0.75:1).

**Figure S11:** ^1^H NMR (C_6_D_6_, 25 ˚C, 400 MHz) of NMR reaction between **2**Na and CO at room temperature.

**Scale-up reaction between CO and 2**Na **at room temperature**

At room temperature, **2**Na (0.3417 g, 0.816 mmol) was dissolved in benzene (2 mL). Then the orange solution was transferred into a 25 mL J. Young tap ampoule. The solution was degassed by the freeze-thaw-vacuum protocol three times then charged with CO (1 atm). The solution changed from orange to dark orange at room temperature for 91 hours. All volatiles were removed *in vacuo*, to form a dark orange oil as the crude product (for ^1^H NMR, see Figure S12). Fraction crystallisation from the crude product allowed us to isolate the product **4**, in a low but reproducible yield. The crystallising details are as below: The crude product was dissolved in ether (15 drops) and placed at −35 ˚C for 18 days to afford a large amount of yellow powder. Then more ether (0.5 mL + 10 drops) was added to fully dissolve the solid and the vial was kept in a −35 ^o^C freezer. After 3 days, yellow crystal (0.0040 g, 0.9 %) of **4** was obtained and monitored by the SCXRD and ^1^H NMR spectrum.

Attempts to isolate other products were not successful.

**Figure S12:** ^1^H NMR (C_6_D_6_, 25 ˚C, 400 MHz) of isolated crystalline **4** and crude product of scale-up reaction between **2**Na and CO at room temperature.

^1^H NMR (400 MHz, C_6_D_6_) δ(ppm) 8.25 (d, ^3^*J*_HH_ = 7.7 Hz, 2H, Ar-*H*), 8.09 (d, ^3^*J*_HH_ = 7.6 Hz, 2H, Ar-*H*), 7.35 (t, ^3^*J*_HH_ = 7.6 Hz, 2H, Ar-*H*), 7.09 (t, ^3^*J*_HH_ = 7.4 Hz, 2H, Ar-*H*), 7.05–6.99 (m, 2H, Ar-*H*), 6.60 (s, 1H, PhC*H*=C(O)C), 5.52 (s, 1H, PhC*H*=C(Si)C), 1.95 (s, 18H, N(C*H_3_*)_2_), 1.64 (s, 12H, NC*H_2_*C*H_2_*N), 0.50 (s, 9H, Si(C*H_3_*)_3_).

**NMR scale reaction between CO and 2**Na **at 60 ^O^C:**

**2**Na (0.0164 g, 0.04 mmol) was dissolved in C_6_D_6_ (0.4809 g, 0.5 mL) and transferred into a J. young tap NMR tube. The NMR tube was degassed by the freeze-thaw-vacuum protocol three times, then charged with CO (1 atm). The colour of mixture changed from orange to brown after reacting at 60 ^o^C for about 26 h and monitored by ^1^H NMR (Figure S13). The reaction is clean (the ratio of products **4** and **5** was about 1: 5).

**Figure S13:** ^1^H NMR (C_6_D_6_, 25 ˚C, 400 MHz) of NMR scale reaction between **2**Na and CO at 60 ^o^C.

**Scale-up reaction between CO and 2**Na **at 60 ^o^C**

**2**Na (0.2849 g, 0.680 mmol) was dissolved in benzene (2 mL). Then the orange solution was transferred into a 25 mL J. Young tap ampoule. The solution was degassed by the freeze-thaw-vacuum protocol three times then charged with CO (1 atm). The solution changed from orange to dark orange after mixing with CO at room temperature. The mixture was stirring at 60 ^o^C for 25 hours and the colour of solution changed from dark orange to brown. All volatiles were removed *in vacuo*, to form a brown oil as the crude product (for ^1^H NMR, see Figure S14). The brown oil was dissolved in toluene (0.5 mL) and placed at −35 ^o^C for 3 days. The orange block crystal of **5** containing toluene molecule (0.0683 g, 20 %) was obtained and characterised by the SCXRD and ^1^H NMR spectrum. The compound **5** which possessed the similar structure to **3** was the main product in this reaction. The ratio of toluene and **5** from NMR was about 0.5 to 1, which was in line with the structure from SCXRD.

**Figure S14:** ^1^H NMR (C_6_D_6_, 25 ˚C, 400 MHz) of isolated crystalline **5** (containing toluene) and crude product of scale-up reaction between **2**Na and CO at 60 ^o^C.

^1^H NMR (400 MHz, C_6_D_6_, 25 ˚C) δ(ppm) 8.26 (d, ^3^*J*_HH_ = 7.4 Hz, 2H, Ar-*H*), 7.31 (t, ^3^*J*_HH_ = 7.6 Hz, 2H, Ar-*H*), 7.00-7.02 (m, overlap with toluene, 1H, Ar-*H*), 5.75 (s, 1H, PhC*H*=C), 2.02 (s, 18H, N(C*H_3_*)_2_), 1.79 (S, 12H, NC*H_2_*C*H_2_*N), 0.47 (s, 9H, Si(C*H_3_*)_3_).

**Figure S15:** ^13^C{^1^H} NMR (176 MHz, C_6_D_6_, 25 ˚C) of **5** (containing toluene).

^13^C{^1^H} NMR (176 MHz, C_6_D_6_, 25 ˚C) δ(ppm) 183.59 (Si*C*(O)=CH), 144.35(Ar*C*), 125.33, 119.31 (Ar*C*H), 104.70 (Ph*C*H=C), 57.52 51.56 (N*C*H_2_*C*H_2_N), 45.48 (N(*C*H_3_)_2_), -0.20 (Si(*C*H_3_)_3_).

**Figure S16:** ^13^C DEPT 135 NMR (176 MHz, C_6_D_6_, 25 ˚C) of **5** (containing toluene)

**Figure S17:** HSQC (C_6_D_6_, 25 ˚C, 700/176 MHz) of **5** (containing toluene). Red: (CH, CH_3_) Blue: (CH_2_)

**Figure S18:** UV/Vis spectrum of **5** (containing toluene) (0.25 mM in hexane, scan internal: 0.5 nm, scan range: 200-800 nm, Shimadzu UV-1800).

**Scale-up reaction between CO and 2**Na **at −20 ^o^C**

**2**Na (0.1250 g, 0.3 mmol) was dissolved in the mixture of methylcyclohexane (2 mL) and benzene (3 mL). Then the orange solution was transferred into a 50 mL Schlenk flask. The solution was degassed by the freeze-thaw-vacuum protocol three times then charged with CO (1 atm) at -20 ^o^C. The solution changed from orange to brownish orange, along with small amount of orange solid. The mixture was reacted at −20 ^o^C for 46 hours. The colour of the solution turned dark brown and a small amount of dark brown oil formed. All volatiles were removed *in vacuo*, to form a dark brown oil as the crude product (for ^1^H NMR, see Figure S19).

**Figure S19:** ^1^H NMR (C_6_D_6_, 25 ˚C, 400 or 300 MHz) of crude product of scale-up reaction between **2**Na and CO at −20 ^o^C, as well as NMR scale reaction between **2**Na and CO at room temperature for 141 hours.

**NMR scale reaction between CO_2_ and 2**Li **at 60 ^o^C**

**2**Li (0.0200 g, 0.05 mmol) was dissolved in C_6_D_6_ (0.4859 g, 0.5 mL) and loaded into a J. young tap NMR tube. The NMR tube was degassed by the freeze-thaw-vacuum protocol three times, then charged with CO_2_ (1 atm). The colour of solution changed from yellow to colourless once mixing with CO_2_. The reaction was heated to 60 ^o^C and monitored by ^1^H/^7^Li NMR spectroscopies (Figure S20 and S21). A small amount of white solids formed after 2h 21min and persisted to the end of monitoring.

**Figure S20:** ^1^H NMR (C_6_D_6_, 25 ˚C, 400 MHz) of NMR scale reaction between **2**Li and CO_2_ at 60 ^o^C.

**Figure S21:** ^7^Li NMR (C_6_D_6_, 25 ˚C, 155 MHz) of NMR scale reaction between **2**Li and CO_2_ at 60 ^o^C.

**Scale-up reaction between CO_2_ and 2**Li **at 60 ^o^C**

**2**Li (0.3203 g, 0.8 mmol) was dissolved in benzene (2 mL). Then the yellow solution was transferred into a 25 mL J. Young tap ampoule. The solution was degassed by the freeze-thaw-vacuum protocol three times then charged with CO_2_ (1 atm). The colour of solution changed from yellow to colourless after mixing with CO_2_ at room temperature. The mixture was stirred at 60 ^o^C for 1.5 hours and a large amount of white solids formed at the bottom of the ampoule. All volatiles were removed *in vacuo*, to form white solids covered by colourless viscous oil as the crude product (for ^1^H NMR, see Figure S22). The crude product was extracted by benzene (0.5 mL × 3) and the extract was filtrated through a glass wool-celite plug. All the volatiles were removed *in vacuo*, resulting white solid. The white solid was fully dissolved in *n*-hexane (0.5 mL) and placed at −35 ^o^C for 3 days. The white block crystals of **6** (0.0308 g, 14 %) were obtained and characterised by SCXRD and ^1^H/^7^Li/^13^C/HSQC NMR spectra.

**Figure S22:** ^1^H NMR (C_6_D_6_, 25 ˚C, 400 MHz) of isolated crystalline **6** and crude product of scale-up reaction between **2**Li and CO_2_ at 60 ^o^C.

^1^H NMR (400 MHz, C_6_D_6_, 25 ˚C) δ(ppm) 7.71 (d, ^3^*J*_HH_ = 7.3 Hz, 2H, Ar-*H*), 7.35 (t, ^3^*J*_HH_ = 7.6 Hz, 2H, Ar-*H*), 7.10-7.18 (m, overlap with C_6_D_6_, 1H, Ar-*H*), 2.50 (t, ^3^*J*_HH_ = 5.7 Hz, 6H, NC*H_2_*C*H_2_*N), 2.06-2.16 (m, 24H, N(C*H_3_*)_2_ and NC*H_2_*C*H_2_*N), 0.30 (s, 18H, Si(C*H_3_*)_3_).

**Figure S23:** ^7^Li NMR (C_6_D_6_, 25 ˚C, 155 MHz) of isolated crystalline **6** and crude product of scale-up reaction between **2**Li and CO_2_ at 60 ^o^C. Inset: Zoom-in view of the ^7^Li signals to display the presence of the minor ^7^Li signals.

^7^Li NMR (155 MHz, C_6_D_6_, 25 ˚C) δ(ppm) 0.82.

**Figure S24:** ^13^C{^1^H} NMR (101 MHz, C_6_D_6_, 25 ˚C) of **6**.

^13^C{^1^H} NMR (101 MHz, C_6_D_6_, 25 ˚C) δ(ppm) 171.21 (O*C*(=O)-C), 142.74 (Ar*C*), 133.51, 126.62, 123.49 (Ar*C*H), 84.21 (Ph*C*C=O), 56.01 49.86 (N*C*H_2_*C*H_2_N), 45.46 (N(*C*H_3_)_2_), 0.99 (Si(*C*H_3_)_3_).

**Figure S25:** HSQC (C_6_D_6_, 25 ˚C, 400/101 MHz) of **6**. Red: (CH, CH_3_) Blue: (CH_2_).

**Figure S26:** UV/Vis spectrum of **6** (0.125 mM in hexane, scan internal: 1.0 nm, scan range: 200-900 nm, Shimadzu UV-1800).

**NMR scale reaction between** **phenyl isothiocyanate and 2**Li **at room temperature**

Phenyl isothiocyanate (0.0056 g, 0.04 mmol) was dissolved in C_6_D_6_ (0.4830 g, 0.5 mL). Then the colourless solution was added into **2**Li (0.0160 g, 0.04 mmol) in a one-portion manner and mixed using a pipette. The yellow solution was transferred into a J. Young tap NMR tube. The reaction was monitored by ^1^H NMR spectroscopy (Figure S27).

**Figure S27:** ^1^H NMR (C_6_D_6_, 25 ˚C, 400 or 300 MHz) of the NMR scale reaction between **2**Li and phenyl isothiocyanate at room temperature.

**Scale-up reaction between phenyl isothiocyanate and 2**Li **at room temperature**

At room temperature, phenyl isothiocyanate (0.0541 g, 0.4 mmol) was dissolved in benzene (2 mL). Then the colourless solution was added into **2**Li (0.1601 g, 0.4 mmol) in a one-portion manner. The golden solution was stirred at room temperature for 1h 15 minutes. All volatiles were removed *in vacuo*, to form a pale orange solid as the crude product (for ^1^H NMR, see Figure S28). The crude product was dissolved in toluene (2 mL) and filtered through a glass wool-celite plug. The filtrate was placed at −40 ^o^C for 1 day. The block crystals of **7** (0.1139 g, 69 %) were obtained and characterised by SCXRD and ^1^H/^7^Li/^13^C/^13^C DEPT 135/HSQC NMR spectra. An impurity with the ^7^Li chemical shift at 0.96 ppm persists despite multiple attempts of recrystallization due to its similar solubility with **7** in ethereal, aliphatic and aromatic solvents. and aromatic solvents.

**Figure S28:** ^1^H NMR (C_6_D_6_, 25 ˚C, 400 MHz) of the isolated crystalline **7** and crude product of scale-up reaction between **2**Li and phenyl isothiocyanate at room temperature.

^1^H NMR (400 MHz, C_6_D_6_, 25 ˚C) δ(ppm) δ 8.78 (d, ^3^*J*_HH_ = 7.9 Hz, 2H, Ar-*H*), 7.42 (d, ^3^*J*_HH_ = 8.2 Hz, 2H, Ar-*H*), 7.36 (t, ^3^*J*_HH_ = 7.6 Hz, 2H, Ar-*H*), 7.23 (t, ^3^*J*_HH_ = 7.6 Hz, 2H, Ar-*H*), 7.11 (t, ^3^*J*_HH_ = 7.3 Hz, 1H, Ar-*H*), 6.82 (t, ^3^*J*_HH_ = 7.2 Hz, 1H, Ar-*H*), 6.69 (s, 1H, C=C*H*(Ph)), 1.96 (s, 18H, N(C*H_3_*)_2_), 1.80-1.72 (m, 6H, NC*H_2_*C*H_2_*N), 1.72-1.65 (m, 6H, NC*H_2_*C*H_2_*N), 0.72 (s, 9H, Si(C*H_3_*)_3_).

**Figure S29:** ^7^Li NMR (C_6_D_6_, 25 ˚C, 155 MHz) of isolated crystalline **7**. Inset: Zoom-in view of the ^7^Li signals to display the presence of the minor ^7^Li signals.

^7^Li NMR (155 MHz, C_6_D_6_, 25 ˚C) δ(ppm) 0.17.

**Figure S30:** ^13^C{^1^H} NMR (101 MHz, C_6_D_6_, 25 ˚C) of **7**. Zoom-in view of the ^13^C signals to display the presence of the minor ^13^C signals near the *C*_6_D_6_ signals.

^13^C{^1^H} NMR (101 MHz, C_6_D_6_, 25 ˚C) δ(ppm) 158.05 (S(N)*C*=C), 150.61, 142.84 (Ar*C*), 128.58, 128.47, 127.42, 123.24, 119.61, 117.42 (Ar*C*H), 122.70 (C=*C*H(Ph)), 56.87, 50.39 (N*C*H_2_*C*H_2_N), 45.51 (N(*C*H_3_)_2_), 2.31 (Si(*C*H_3_)_3_).

**Figure S31:** ^13^C DEPT 135 NMR (101 MHz, C_6_D_6_, 25 ˚C) of **7**.

**Figure S32:** HSQC (C_6_D_6_, 25 ˚C, 400/101 MHz) of **7**.

**NMR scale reaction between phenyl isothiocyanate and 2**Na **at room temperature**

Phenyl isothiocyanate (0.0055 g, 0.04 mmol) was dissolved in C_6_D_6_ (0.4887 g, 0.5 mL). Then the colourless solution was added into **2**Na (0.0166 g, 0.04 mmol) in a one-portion manner and mixed using a pipette. The yellow solution was transferred into a J. Young tap NMR tube. The reaction was monitored by ^1^H NMR spectroscopy (Figure S33).

**Figure S33:** ^1^H NMR (C_6_D_6_, 25 ˚C, 300 MHz) of NMR scale reaction between **2**Na and phenyl isothiocyanate at room temperature.

**Scale-up reaction between phenyl isothiocyanate and 2**Na **at room temperature**

At room temperature, phenyl isothiocyanate (0.0542 g, 0.4 mmol) was dissolved in benzene (2 mL). Then the colourless solution was added into **2**Na (0.1664 g, 0.4 mmol) in a one-portion manner. The pale orange solution was stirred at room temperature for 35 minutes. All volatiles were removed *in vacuo*, to form an orange solid as the crude product (for ^1^H NMR, see Figure S34). The crude product was dissolved in toluene (1 mL) and filtered through a glass wool-celite plug. The filtrate was placed at −40 ^o^C for 5 days. The pale-orange block crystals of **8** (0.1012 g, 46 %) were obtained and characterised by SCXRD and ^1^H/^23^Na/^13^C/^13^C DEPT 135/HSQC NMR spectra.

**Figure S34:** ^1^H NMR (C_6_D_6_, 25 ˚C, 400 MHz) of isolated crystalline **8** and crude product of scale-up reaction between **2**Na and phenyl isothiocyanate at room temperature.

^1^H NMR (400 MHz, C_6_D_6_, 25 ˚C) δ(ppm) δ 8.79 (d, ^3^*J*_HH_ = 7.6 Hz, 2H, Ar-*H*), 7.43 (d, ^3^*J*_HH_ = 7.8 Hz, 2H, Ar-*H*), 7.32 (t, ^3^*J*_HH_ = 7.7 Hz, 2H, Ar-*H*), 7.21 (t, ^3^*J*_HH_ = 7.8 Hz, 2H, Ar-*H*), 7.07 (t, ^3^*J*_HH_ = 7.3 Hz, 1H, Ar-*H*), 6.81 (t, ^3^*J*_HH_ = 7.2 Hz, 1H, Ar-*H*), 6.65 (s, 1H, C=C*H*(Ph)), 1.88 (s, 18H, N(C*H_3_*)_2_), 1.62 (s, 12H, NC*H_2_*C*H_2_*N), 0.74 (s, 9H, Si(C*H_3_*)_3_).

**Figure S35:** ^23^Na NMR (C_6_D_6_, 25 ˚C, 106 MHz) of isolated crystalline **8**.

^23^Na NMR (106 MHz, C_6_D_6_, 25 ˚C) δ(ppm) 11.16 (br).

**Figure S36:** ^13^C{^1^H} NMR (101 MHz, C_6_D_6_, 25 ˚C) of **8**. Zoom-in view of the ^13^C signals to display the presence of the minor ^13^C signals near the *C*_6_D_6_ signals.

^13^C{^1^H} NMR (101 MHz, C_6_D_6_, 25 ˚C) δ(ppm) 159.21 (S(N)*C*=C), 150.84, 143.16 (Ar*C*), 128.66, 127.53, 122.92, 119.38, 117.19 (Ar*C*H), 121.30 (C=*C*H(Ph)), 56.92, 50.83 (N*C*H_2_*C*H_2_N), 45.08 (N(*C*H_3_)_2_), 2.47 (Si(*C*H_3_)_3_).

**Figure S37:** ^13^C DEPT 135 NMR (101 MHz, C_6_D_6_, 25 ˚C) of **8**.

**Figure S38:** HSQC (C_6_D_6_, 25 ˚C, 400/101 MHz) of **8**.

**NMR scale reaction between *tert*-butyl isothiocyanate and 2**Na **at room temperature**

*tert*-Butyl isothiocyanate (0.0046 g, 0.04 mmol) was dissolved in C_6_D_6_ (0.4892 g, 0.5 mL). Then the colourless solution was added into **2**Na (0.0166 g, 0.04 mmol) in a one-portion manner and mixed using a pipette. The pale pink solution was transferred into a J. Young tap NMR tube. The reaction was monitored by ^1^H NMR spectroscopy (Figure S39). The colour of solution turned pale yellow after 10 minutes.

**Figure S39:** ^1^H NMR (C_6_D_6_, 25 ˚C, 300 MHz) of NMR scale reaction between **2**Na and *tert*-butyl isothiocyanate at room temperature.

**Scale-up reaction between *tert*-butyl isothiocyanate and 2**Na **at room temperature**

At room temperature, a colourless solution of *tert*-butyl isothiocyanate in benzene (0.0462 g, 0.4 mmol, 2 mL) was added to an orange solution of **2**Na in benzene (0.1668 g, 0.4 mmol, 2 mL) in a one-portion manner. Upon mixing, the colour changed from orange to pale pink immediately. The reaction was stirred at room temperature for 10 minutes. All volatiles were removed *in vacuo*, to form a mixture of white solid and a small amount of pink oil as the crude product (for ^1^H NMR, see Figure S40). The crude product was dissolved in a mixture of *n*-hexane (16 drops) and toluene (23 drops) and placed at −40 ^o^C for 5 days. The white block crystal of **9** (0.0382 g, 18 %) was obtained and characterised by SCXRD and ^1^H/^23^Na/^13^C/^13^C DEPT 135/HSQC NMR spectra.

**Figure S40:** ^1^H NMR (C_6_D_6_, 25 ˚C, 400 MHz) of isolated crystalline **9** and crude product of scale-up reaction between **2**Na and *tert*-butyl isothiocyanate at room temperature.

^1^H NMR (400 MHz, C_6_D_6_, 25 ˚C) δ(ppm) δ 7.83 (d, ^3^*J*_HH_ = 7.5 Hz, 2H, Ar-*H*), 7.27 (t, ^3^*J*_HH_ = 7.5 Hz, 2H, Ar-*H*), 7.07 (t, ^3^*J*_HH_ = 7.2 Hz, 1H, Ar-*H*), 3.92 (s, 1H, C*H*(SiMe_3_)(Ph)), 2.08 (s, 9H, C(CH_3_)_3_) 1.90 (s, 18H, N(C*H_3_*)_2_), 1.60 (s, 12H, NC*H_2_*C*H_2_*N), 0.46 (s, 9H, Si(C*H_3_*)_3_).

**Figure S41:** ^23^Na NMR (C_6_D_6_, 25 ˚C, 106 MHz) of isolated crystalline **9**.

^23^Na NMR (106 MHz, C_6_D_6_, 25 ˚C) δ(ppm) 12.82 (br).

**Figure S42:** ^13^C{^1^H} NMR (101 MHz, C_6_D_6_, 25 ˚C) of **9**. Inset: a zoom-in view of the ^13^C signals to display the presence of the ^13^C signals in aromatic region.

^13^C{^1^H} NMR (101 MHz, C_6_D_6_, 25 ˚C) δ(ppm) 177.04 (S(C)*C*=N), 145.40 (Ar*C*), 127.55, 123.75 (Ar*C*H), 65.63 (*C*H(SiMe_3_)(Ph)), 55.59 (*C*(CH_3_)_3_), 56.75, 50.69 (N*C*H_2_*C*H_2_N), 45.04 (N(*C*H_3_)_2_), 29.64 (C(*C*H_3_)_3_), -0.48 (Si(*C*H_3_)_3_).

**Figure S43:** ^13^C DEPT 135 NMR (101 MHz, C_6_D_6_, 25 ˚C) of **9**.

**Figure S44:** HSQC (C_6_D_6_, 25 ˚C, 400/101 MHz) of **9**.

**NMR scale reaction between *tert*-butyl isothiocyanate and 2**Na **at 60 ^o^C**

*tert*-Butyl isothiocyanate (0.0047 g, 0.04 mmol) was dissolved in C_6_D_6_ (0.4892 g, 0.5 mL). Then the colourless solution was added into **2**Na (0.0166 g, 0.04 mmol) in a one-portion manner and mixed using a pipette. The pale pink solution was transferred into a J. Young tap NMR tube. The reaction was heated to 60 ^o^C and monitored by ^1^H NMR spectroscopy (Figure S45). The colour of solution turned pale yellow after 30 minutes, along with a large amount of white solid. The signals of the ketene imine product match with those reported,^[[2]](#endnote-2)^ though CDCl_3_ was used in the literature.

**Figure S45:** ^1^H NMR (C_6_D_6_, 25 ˚C, 300 MHz) of NMR scale reaction between **2**Na and *tert*-butyl isothiocyanate at 60 ^o^C.

**Scale-up reaction between *tert*-butyl isothiocyanate and 2Na at 60 ^o^C**

At room temperature, a colourless solution of *tert*-butyl isothiocyanate in benzene (0.0462 g, 0.4 mmol, 2 mL) was added to an orange solution of **2**Na in benzene (0.1668 g, 0.4 mmol, 2 mL) in a one-portion manner. Upon mixing, the colour changed from orange to pale pink immediately. The reaction was heated at 60 ^o^C and stirred for 41 minutes. All volatiles were removed *in vacuo*, to form a yellow oil and a small amount of colourless solid as the crude product (for ^1^H NMR, see Figure S46). The crude product was dissolved in a mixture of *n*-hexane (5 drops) and toluene (5 drops) and placed at −40 ^o^C for 3 days. The colourless block crystal of **10** (0.0491 g, 34 %) was obtained and characterised by SCXRD and ^1^H/^23^Na/^13^C/^13^C DEPT 135/HSQC NMR spectra.

**Figure S46:** ^1^H NMR (C_6_D_6_, 25 ˚C, 400 or 300 MHz) of isolated crystalline **10** and crude product of scale-up reaction between **2**Na and *tert*-butyl isothiocyanate at 60 ^o^C.

^1^H NMR (400 MHz, C_6_D_6_, 25 ˚C) δ(ppm) 2.08 (s, 18H, N(C*H_3_*)_2_), 1.80 (s, 12H, NC*H_2_*C*H_2_*N), 0.79 (s, 9H, Si(C*H_3_*)_3_).

**Figure S47:** ^23^Na NMR (C_6_D_6_, 25 ˚C, 106 MHz) of isolated crystalline **10**.

^23^Na NMR (106 MHz, C_6_D_6_, 25 ˚C) δ(ppm) 17.66 (br).

**Figure S48:** ^13^C{^1^H} NMR (101 MHz, C_6_D_6_, 25 ˚C) of **10**.

^13^C{^1^H} NMR (101 MHz, C_6_D_6_, 25 ˚C) δ(ppm) 56.91, 50.07 (N*C*H_2_*C*H_2_N), 45.32 (N(*C*H_3_)_2_), 9.58 (Si(*C*H_3_)_3_).

**Figure S49:** ^13^C DEPT 135 NMR (101 MHz, C_6_D_6_, 25 ˚C) of **10**.

**Figure S50:** HSQC (C_6_D_6_, 25 ˚C, 400/101 MHz) of **10**.

**NMR scale reaction between bis(*tert*-butyl)carbodiimide and 2**Li **at room temperature, then 60 ^o^C**

Bis(*tert*-butyl)carbodiimide (0.0062 g, 0.04 mmol) was dissolved in C_6_D_6_ (0.4876 g, 0.5 mL). Then the colourless solution was added into **2**Li (0.0160 g, 0.04 mmol) in a one-portion manner and mixed using a pipette. The yellow solution was transferred into a J. Young tap NMR tube. The reaction was monitored by ^1^H NMR spectroscopy (Figure S51). No reaction took place for 21 hours at room temperature. Upon heating to 60 ^o^C, **2**Li decomposed within 24 hours.

**Figure S51:** ^1^H NMR (C_6_D_6_, 25 ˚C, 400 or 300 MHz) of NMR scale reaction between **2**Li and bis(tert-butyl)carbodiimide at room temperature, then 60 ^o^C.

**NMR scale reaction between bis(*tert*-butyl)carbodiimide and 2**Na **at room temperature, then 60 ^o^C**

Bis(*tert*-butyl)carbodiimide (0.0062 g, 0.04 mmol) was dissolved in C_6_D_6_ (0.4890 g, 0.5 mL). Then the colourless solution was added into **2**Na (0.0166 g, 0.04 mmol) in a one-portion manner and mixed using a pipette. The yellow solution was transferred into a J. Young tap NMR tube. The reaction was monitored by ^1^H NMR spectroscopy (Figure S52). No reactions took place at room temperature for 4 days. Upon heating to 60 ^o^C, **2**Na decomposed within 18 hours.

**Figure S52:** ^1^H NMR (C_6_D_6_, 25 ˚C, 400 or 300 MHz) of NMR scale reaction between **2**Na and bis(tert-butyl)carbodiimide at room temperature, then 60 ^o^C.

**Reaction between bis(trimethylsilyl)carbodiimide and 2**Li **at room temperature**

Bis(trimethylsilyl)carbodiimide (0.0075 g, 0.04 mmol) was dissolved in C_6_D_6_ (0.4903 g, 0.5 mL). Then the colourless solution was added into **2**Li (0.0160 g, 0.04 mmol) in a one-portion manner and mixed using a pipette. The colour of the solution changed from yellow to pale pink within 1 minute. The pale pink solution was transferred into a J. Young tap NMR tube. The reaction was monitored by ^1^H NMR spectroscopy (Figure S53).

**Figure S53:** ^1^H NMR (C_6_D_6_, 25 ˚C, 400 or 300 MHz) of NMR scale reaction between **2**Li and bis(trimethylsilyl)carbodiimide at room temperature.

**Scale-up reaction between bis(trimethylsilyl)carbodiimide and 2**Li **at room temperature**

At room temperature, bis(trimethylsilyl)carbodiimide (0.0746 g, 0.4 mmol) was dissolved in benzene (2 mL). Then the colourless solution was added into **2**Li (0.1604 g, 0.4 mmol) in a one-portion manner. Upon mixing with a pipette, the colour of solution changed from orange to magenta within 2 minutes., After stirring at room for 1 hour, the pink solution was exposed to vacuum and all volatiles were removed, to form a pink crystalline crude product (for ^1^H NMR, see Figure S54). The crude product was dissolved in toluene (18 drops) and placed at −40 ^o^C for 1 day. Pink block crystals of **11** (0.0802 g, 57 %) were obtained and characterised by SCXRD and ^1^H/^7^Li/^13^C/^13^C DEPT 135/HSQC NMR spectra.

**Figure S54:** ^1^H NMR (C_6_D_6_, 25 ˚C, 400 MHz) of isolated crystalline **11** and crude product of scale-up reaction between **2**Li and bis(trimethylsilyl)carbodiimide at room temperature. In the crude product, bis(trimethylsilyl)phenylmethane (dark square) was presented in a 1:1 ratio to **11**.

^1^H NMR (400 MHz, C_6_D_6_, 25 ˚C) δ(ppm) 2.08 (m, 18H, N(C*H_3_*)_2_), 1.76 (br, 12H, NC*H_2_*C*H_2_*N), 0.54 (s, 9H, Si(C*H_3_*)_3_).

**Figure S55:** ^7^Li NMR (C_6_D_6_, 25 ˚C, 155 MHz) of isolated crystalline **11**.

^7^Li NMR (155 MHz, C_6_D_6_, 25 ˚C) δ(ppm) -0.26.

**Figure S56:** ^13^C{^1^H} NMR (101 MHz, C_6_D_6_, 25 ˚C) of **11**.

^13^C{^1^H} NMR (101 MHz, C_6_D_6_, 25 ˚C) δ(ppm) 56.59, 50.58 (N*C*H_2_*C*H_2_N), 45.44 (N(*C*H_3_)_2_), 3.20 (Si(*C*H_3_)_3_). The signal of the tertiary carbon, N=*C*=N, is missing due to the solubility limit in *d*_6_-benzene.

**Figure S57:** ^13^C DEPT 135 NMR (101 MHz, C_6_D_6_, 25 ˚C) of **11**.

**Figure S58:** HSQC (C_6_D_6_, 25 ˚C, 400/101 MHz) of **11**.

**NMR scale reaction between bis(trimethylsilyl)carbodiimide and 2**Na **at room temperature**

Bis(trimethylsilyl)carbodiimide (0.0076 g, 0.04 mmol) was dissolved in C_6_D_6_ (0.4890 g, 0.5 mL). Then the colourless solution was added into **2**Na (0.0165 g, 0.04 mmol) in a one-portion manner and mixed using a pipette. A large amount of colourless gel and pale orange solution resulted. The pale orange solution was transferred into a J. Young tap NMR tube. The reaction was monitored by ^1^H NMR spectroscopies (Figure S59). The signals of PhCH_2_SiMe_3_ match with those reported.^[[3]](#endnote-3)^

**Figure S59:** ^1^H NMR (C_6_D_6_, 25 ˚C, 300 MHz) of NMR scale reaction between **2**Na and bis(trimethylsilyl)carbodiimide at room temperature.

1.3 Single-crystal X-ray diffraction (SCXRD) Details

Crystal structure data **3**, **4**, **5** and **6** were collected on a Rigaku XtaLAB Synergy-S, Single source at home/near, HyPix-Arc 100 diffractometer. Crystal structure data for **7**, **8** and **11** were measured on a Rigaku XtaLAB Synergy, Dualflex, using a HyPix detector while the dataset for **9** and **10** was measured on an Agilent SuperNova diffractometer using an Atlas detector. Intensities were corrected for absorption using a multifaceted crystal model.^[[4]](#endnote-4)^ All diffractometers were equipped with a fine-focus sealed X-ray tube and an Oxford Cryosystems CryostreamPlus open-flow N_2_ cooling device. Cell refinement, data collection and data reduction were undertaken via software CrysAlisPro.^[[5]](#endnote-5)^

Using Olex2,^[[6]](#endnote-6)^ the structures of **3**, **4**, **5**, **6**, **8**, **9**, **10** and **11** was solved using SHELXT^[[7]](#endnote-7)^ while **7** was solved using olex2.solve^[[8]](#endnote-8)^ and all structures were refined by SHELXL.^[[9]](#endnote-9)^ All non-hydrogen atoms were refined anisotropically and hydrogen atoms were positioned with idealised geometry. The displacement parameters of the hydrogen atoms were constrained using a riding model with U_(H)_ set to be an appropriate multiple of the U_eq_ value of the parent atom.

**5**: The carbons in Me_6_Tren (C12A – C23A / C12B – C23B) are disordered over two positions at a percentage occupancy ratio of 58.52 : 41.48.

**6**: The group in Me_6_Tren (C20A – C27A, N1A, N3A – N4A / C20B – C27B, N1B, N3B – N4B) are disordered over two positions at a percentage occupancy ratio of 66.89 : 33.11.

**7:** The whole Me_6_Tren ligand (C1A – C12A, N1A – N4A / C1B – C12B, N1B – N4B), phenyl group (C15A – C20A / C15B – C20B) and toluene molecular (C30A – C36A / C30B – C36B) are disordered over two positions at a refined percentage occupancy ratio of 67.3 (2) : 32.7 (2).

**11**: The structure contains two crystallographically-independent Li(Me_6_Tren)(NCNSiMe_3_) molecules per asymmetric unit.

**Table S1**. Crystal Structure Refinement Details for Complexes **3**, **4** and **5**.

| **Compound** | **3** | **4** | **5** |
| --- | --- | --- | --- |
| Empirical formula | C_23_H_45_LiN_4_OSi | C_31_H_51_N_4_NaOSi | C_53_H_98_N_8_Na_2_O_2_Si_2_ |
| Formula weight | 428.66 | 546.83 | 981.55 |
| Temperature/K | 150.0(2) | 150.0(2) | 150.0(2) |
| Crystal system | monoclinic | monoclinic | monoclinic |
| Space group | P2_1_/c | P2_1_/n | P2_1_/n |
| a/Å | 9.1081(3) | 10.5547(3) | 8.9493(3) |
| b/Å | 14.9189(6) | 18.8179(6) | 17.1024(5) |
| c/Å | 19.8648(6) | 16.9275(7) | 20.0919(6) |
| α/° | 90 | 90 | 90 |
| β/° | 97.634(3) | 98.233(4) | 90.422(3) |
| γ/° | 90 | 90 | 90 |
| Volume/Å^3^ | 2675.36(16) | 3327.4(2) | 3075.07(16) |
| Z | 4 | 4 | 2 |
| ρ_calc_g/cm^3^ | 1.064 | 1.092 | 1.060 |
| μ/mm^‑1^ | 0.907 | 0.953 | 0.978 |
| F(000) | 944.0 | 1192.0 | 1076.0 |
| Crystal size/mm^3^ | 0.33 × 0.11 × 0.02 | 0.12 × 0.07 × 0.05 | 0.18 × 0.1 × 0.06 |
| Radiation | CuKα (λ = 1.54184) | CuKα (λ = 1.54184) | CuKα (λ = 1.54184) |
| 2θ range for data collection/° | 7.434 to 153.744 | 7.064 to 154.922 | 6.788 to 154.878 |
| Index ranges | -11 ≤ h ≤ 11, -10 ≤ k ≤ 18, -24 ≤ l ≤ 24 | -11 ≤ h ≤ 13, -23 ≤ k ≤ 23, -21 ≤ l ≤ 21 | -10 ≤ h ≤ 9, -21 ≤ k ≤ 21, -20 ≤ l ≤ 25 |
| Reflections collected | 20736 | 40885 | 30069 |
| Independent reflections | 5323 [R_int_ = 0.0297, R_sigma_ = 0.0286] | 6773 [R_int_ = 0.0325, R_sigma_ = 0.0207] | 5942 [R_int_ = 0.0314, R_sigma_ = 0.0246] |
| Data/restraints/parameters | 5323/0/281 | 6773/0/352 | 5942/887/458 |
| Goodness-of-fit on F^2^ | 1.051 | 1.112 | 1.059 |
| Final R indexes [I>=2σ (I)] | R_1_ = 0.0374, wR_2_ = 0.0961 | R_1_ = 0.0747, wR_2_ = 0.1650 | R_1_ = 0.0384, wR_2_ = 0.1031 |
| Final R indexes [all data] | R_1_ = 0.0453, wR_2_ = 0.1006 | R_1_ = 0.0869, wR_2_ = 0.1717 | R_1_ = 0.0497, wR_2_ = 0.1096 |
| Largest diff. peak/hole / e Å^-3^ | 0.30/-0.22 | 0.33/-0.33 | 0.21/-0.22 |
|  |  |  |  |

**Table S2**. Crystal Structure Refinement Details for Complexes **6**, **7** and **8**.

| **Compound** | **6** | **7** | **8** |
| --- | --- | --- | --- |
| Empirical formula | C_27_H_53_LiN_4_O_4_Si_2_ | C_36_H_58_LiN_5_SSi | C_29_H_50_N_5_NaSiS |
| Formula weight | 560.85 | 627.96 | 551.88 |
| Temperature/K | 150.0(2) | 100.00(10) | 100.00(10) |
| Crystal system | orthorhombic | monoclinic | monoclinic |
| Space group | P2_1_2_1_2_1_ | P2_1_/c | P2_1_ |
| a/Å | 11.6161(2) | 19.5097(2) | 9.53941(4) |
| b/Å | 13.2126(2) | 11.21320(10) | 11.08438(4) |
| c/Å | 22.2524(3) | 17.2329(2) | 15.39311(5) |
| α/° | 90 | 90 | 90 |
| β/° | 90 | 97.3760(10) | 99.9903(3) |
| γ/° | 90 | 90 | 90 |
| Volume/Å^3^ | 3415.27(9) | 3738.78(7) | 1602.964(9) |
| Z | 4 | 4 | 2 |
| ρ_calc_g/cm^3^ | 1.091 | 1.116 | 1.143 |
| μ/mm^‑1^ | 1.208 | 1.293 | 1.570 |
| F(000) | 1224.0 | 1368.0 | 600.0 |
| Crystal size/mm^3^ | 0.2 × 0.07 × 0.04 | 0.12 × 0.1 × 0.06 | 0.18 × 0.13 × 0.06 |
| Radiation | CuKα (λ = 1.54184) | Cu Kα (λ = 1.54184) | Cu Kα (λ = 1.54184) |
| 2θ range for data collection/° | 7.782 to 154.386 | 9.116 to 159.01 | 9.414 to 158.658 |
| Index ranges | -12 ≤ h ≤ 14, -16 ≤ k ≤ 16, -25 ≤ l ≤ 28 | -24 ≤ h ≤ 23, -14 ≤ k ≤ 8, -21 ≤ l ≤ 21 | -12 ≤ h ≤ 11, -13 ≤ k ≤ 14, -19 ≤ l ≤ 19 |
| Reflections collected | 37598 | 27328 | 121113 |
| Independent reflections | 6809 [R_int_ = 0.0357, R_sigma_ = 0.0264] | 7797 [R_int_ = 0.0327, R_sigma_ = 0.0334] | 6583 [R_int_ = 0.0450, R_sigma_ = 0.0139] |
| Data/restraints/parameters | 6809/791/458 | 7797/264/676 | 6583/1/343 |
| Goodness-of-fit on F^2^ | 1.048 | 1.050 | 1.061 |
| Final R indexes [I>=2σ (I)] | R_1_ = 0.0301, wR_2_ = 0.0732 | R_1_ = 0.0366, wR_2_ = 0.0939 | R_1_ = 0.0232, wR_2_ = 0.0605 |
| Final R indexes [all data] | R_1_ = 0.0351, wR_2_ = 0.0757 | R_1_ = 0.0433, wR_2_ = 0.0980 | R_1_ = 0.0234, wR_2_ = 0.0606 |
| Largest diff. peak/hole / e Å^-3^ | 0.16/-0.20 | 0.24/-0.26 | 0.18/-0.18 |
| Flack parameter | -0.013(9) | - | 0.004(5) |

**Table S3**. Crystal Structure Refinement Details for Complexes **9**, **10** and **11**.

| **Compound** | **9** | **10** | **11** |
| --- | --- | --- | --- |
| Empirical formula | C_27_H_54_N_5_NaSSi | C_15_H_39_N_4_NaSSi | C_16_H_39_LiN_6_Si |
| Formula weight | 531.89 | 358.64 | 350.56 |
| Temperature/K | 100.02(12) | 100.00(10) | 100.00(10) |
| Crystal system | orthorhombic | monoclinic | orthorhombic |
| Space group | P2_1_2_1_2_1_ | P2_1_/n | P2_1_2_1_2_1_ |
| a/Å | 11.6578(4) | 9.7589(3) | 13.14800(10) |
| b/Å | 16.1438(5) | 18.6713(6) | 17.82580(10) |
| c/Å | 17.7776(6) | 12.3973(4) | 18.96880(10) |
| α/° | 90 | 90 | 90 |
| β/° | 90 | 90.891(3) | 90 |
| γ/° | 90 | 90 | 90 |
| Volume/Å^3^ | 3345.77(19) | 2258.66(12) | 4445.79(5) |
| Z | 4 | 4 | 8 |
| ρ_calc_g/cm^3^ | 1.056 | 1.055 | 1.047 |
| μ/mm^‑1^ | 1.483 | 1.974 | 0.986 |
| F(000) | 1168.0 | 792.0 | 1552.0 |
| Crystal size/mm^3^ | 0.15 × 0.13 × 0.1 | 0.11 × 0.09 × 0.07 | 0.47 × 0.25 × 0.16 |
| Radiation | Cu Kα (λ = 1.54184) | Cu Kα (λ = 1.54184) | Cu Kα (λ = 1.54184) |
| 2θ range for data collection/° | 7.396 to 136.502 | 8.562 to 154.406 | 6.804 to 158.56 |
| Index ranges | -14 ≤ h ≤ 14, -19 ≤ k ≤ 18, -21 ≤ l ≤ 17 | -12 ≤ h ≤ 12, -23 ≤ k ≤ 8, -15 ≤ l ≤ 14 | -16 ≤ h ≤ 16, -20 ≤ k ≤ 22, -22 ≤ l ≤ 24 |
| Reflections collected | 31968 | 12407 | 44676 |
| Independent reflections | 6116 [R_int_ = 0.1033, R_sigma_ = 0.0686] | 4675 [R_int_ = 0.0415, R_sigma_ = 0.0529] | 9343 [R_int_ = 0.0369, R_sigma_ = 0.0247] |
| Data/restraints/parameters | 6116/0/328 | 4675/0/208 | 9343/0/452 |
| Goodness-of-fit on F^2^ | 1.051 | 1.033 | 1.073 |
| Final R indexes [I>=2σ (I)] | R_1_ = 0.0570, wR_2_ = 0.1334 | R_1_ = 0.0391, wR_2_ = 0.0970 | R_1_ = 0.0332, wR_2_ = 0.0903 |
| Final R indexes [all data] | R_1_ = 0.0730, wR_2_ = 0.1468 | R_1_ = 0.0564, wR_2_ = 0.1046 | R_1_ = 0.0346, wR_2_ = 0.0912 |
| Largest diff. peak/hole / e Å^-3^ | 0.35/-0.24 | 0.43/-0.24 | 0.21/-0.22 |
| Flack parameter | -0.020(18) |  | 0.16(2) |

**Table S4**. Key bond lengths and angles for complex **3**.

| Key bond lengths | | |  | Key bond angles | | | |
| --- | --- | --- | --- | --- | --- | --- | --- |
| **Atom** | **Atom** | **Length/Å** |  | **Atom** | **Atom** | **Atom** | **Angle/** **˚** |
| Li1 | N1 | 2.111(3) |  | O1 | Li1 | N1 | 111.66(11) |
| Li1 | N2 | 2.277(2) |  | O1 | Li1 | N2 | 140.85(13) |
| Li1 | N3 | 2.086(2) |  | O1 | Li1 | N3 | 112.36(12) |
| Li1 | O1 | 1.791(2) |  | N1 | Li1 | N2 | 86.16(9) |
| C13 | O1 | 1.3084(15) |  | N3 | Li1 | N1 | 121.66(12) |
| C13 | C14 | 1.3733(18) |  | N3 | Li1 | N2 | 83.05(9) |
| C13 | Si1 | 1.8952(13) |  | C13 | O1 | Li1 | 154.23(11) |
| C14 | C15 | 1.4580(18) |  | O1 | C13 | Si1 | 114.08(9) |
|  |  |  |  | O1 | C13 | C14 | 125.92(11) |
|  |  |  |  | C14 | C13 | Si1 | 119.95(9) |
|  |  |  |  | C13 | C14 | C15 | 129.09(11) |

**Table S5**. Key bond lengths and angles for complex **4**.

| Key bond lengths | | |  | Key bond angles | | | |
| --- | --- | --- | --- | --- | --- | --- | --- |
| **Atom** | **Atom** | **Length/Å** |  | **Atom** | **Atom** | **Atom** | **Angle/ ˚** |
| Na1 | N1 | 2.549(3) |  | O1 | Na1 | N1 | 173.29(10) |
| Na1 | N2 | 2.511(3) |  | O1 | Na1 | N2 | 102.97(9) |
| Na1 | N3 | 2.447(3) |  | O1 | Na1 | N3 | 106.02(10) |
| Na1 | N4 | 2.491(3) |  | O1 | Na1 | N4 | 114.21(10) |
| Na1 | O1 | 2.1696(19) |  | N2 | Na1 | N1 | 71.78(9) |
| C13 | O1 | 1.274(3) |  | N3 | Na1 | N1 | 72.92(11) |
| C13 | C14 | 1.511(3) |  | N3 | Na1 | N2 | 112.21(10) |
| C13 | C22 | 1.383(3) |  | N3 | Na1 | N4 | 108.44(10) |
| C22 | C23 | 1.440(3) |  | N4 | Na1 | N1 | 72.14(11) |
| C14 | Si1 | 1.876(3) |  | N4 | Na1 | N2 | 112.79(11) |
| C14 | C15 | 1.346(4) |  | C13 | O1 | Na1 | 162.55(17) |
| C15 | C16 | 1.472(4) |  | O1 | C13 | C14 | 115.1(2) |
|  |  |  |  | O1 | C13 | C22 | 126.9(2) |
|  |  |  |  | C22 | C13 | C14 | 117.9(2) |
|  |  |  |  | C13 | C22 | C23 | 127.1(2) |
|  |  |  |  | C13 | C14 | Si1 | 114.95(17) |
|  |  |  |  | C15 | C14 | Si1 | 121.2(2) |
|  |  |  |  | C15 | C14 | C13 | 123.6(2) |
|  |  |  |  | C14 | C15 | C16 | 130.2(2) |

**Table S6**. Key bond lengths and angles for complex **5**.

| Key bond lengths | | |  | Key bond angles | | | |
| --- | --- | --- | --- | --- | --- | --- | --- |
| **Atom** | **Atom** | **Length/Å** |  | **Atom** | **Atom** | **Atom** | **Angle/** **˚** |
| Na1 | N1 | 2.5189(13) |  | O1 | Na1 | N1 | 162.19(4) |
| Na1 | N2 | 2.6293(13) |  | O1 | Na1 | N2 | 125.97(4) |
| Na1 | N3 | 2.5027(13) |  | O1 | Na1 | N3 | 96.49(5) |
| Na1 | N4 | 2.4590(13) |  | O1 | Na1 | N4 | 99.57(5) |
| Na1 | O1 | 2.1690(11) |  | N1 | Na1 | N2 | 71.79(4) |
| C1 | O1 | 1.2968(16) |  | N3 | Na1 | N1 | 72.62(5) |
| C1 | C2 | 1.3728(18) |  | N3 | Na1 | N2 | 112.85(5) |
| C1 | Si1 | 1.8847(14) |  | N4 | Na1 | N1 | 73.17(5) |
| C2 | C3 | 1.4506(18) |  | N4 | Na1 | N2 | 107.78(5) |
|  |  |  |  | N4 | Na1 | N3 | 113.51(5) |
|  |  |  |  | C1 | O1 | Na1 | 157.72(9) |
|  |  |  |  | O1 | C1 | Si1 | 112.09(9) |
|  |  |  |  | O1 | C1 | C2 | 126.39(12) |
|  |  |  |  | C2 | C1 | Si1 | 121.51(10) |
|  |  |  |  | C1 | C2 | C3 | 128.96(12) |

**Table S7**. Key bond lengths and angles for complex **6**.

| Key bond lengths | | |  | Key bond angles | | | |
| --- | --- | --- | --- | --- | --- | --- | --- |
| **Atom** | **Atom** | **Length/Å** |  | **Atom** | **Atom** | **Atom** | **Angle/** **˚** |
| Li1 | N1A | 2.342(8) |  | O1 | Li1 | O2 | 90.90(15) |
| Li1 | N2 | 2.160(4) |  | O1 | Li1 | N2 | 118.29(19) |
| Li1 | N3A | 2.204(9) |  | O1 | Li1 | N1A | 99.4(2) |
| Li1 | O1 | 1.912(4) |  | O1 | Li1 | N3A | 111.9(3) |
| Li1 | O2 | 1.961(4) |  | O2 | Li1 | N2 | 94.84(16) |
| C1 | O1 | 1.249(2) |  | O2 | Li1 | N1A | 169.7(2) |
| C1 | O3 | 1.359(2) |  | O2 | Li1 | N3A | 96.8(3) |
| C1 | C2 | 1.418(3) |  | N2 | Li1 | N1A | 80.34(19) |
| C3 | O2 | 1.241(2) |  | N2 | Li1 | N3A | 128.2(3) |
| C3 | O4 | 1.367(2) |  | N3A | Li1 | N1A | 79.5(3) |
| C3 | C2 | 1.418(3) |  | O1 | C1 | O3 | 117.06(17) |
| C2 | C4 | 1.496(3) |  | O1 | C1 | C2 | 127.77(18) |
|  |  |  |  | O3 | C1 | C2 | 115.17(16) |
|  |  |  |  | C1 | C2 | C3 | 118.80(17) |
|  |  |  |  | C1 | C2 | C4 | 120.44(17) |
|  |  |  |  | C3 | C2 | C4 | 120.74(18) |
|  |  |  |  | O2 | C3 | O4 | 117.34(18) |
|  |  |  |  | O2 | C3 | C2 | 128.05(19) |
|  |  |  |  | O4 | C3 | C2 | 114.60(17) |
|  | | | | | | | |
| Li1 | N1B | 2.337(14) |  | O1 | Li1 | N1B | 107.8(4) |
| Li1 | N3B | 2.222(16) |  | O1 | Li1 | N3B | 105.1(5) |
|  |  |  |  | O2 | Li1 | N1B | 161.0(4) |
|  |  |  |  | O2 | Li1 | N3B | 95.5(4) |
|  |  |  |  | N2 | Li1 | N1B | 79.1(4) |
|  |  |  |  | N2 | Li1 | N3B | 135.1(4) |
|  |  |  |  | N3B | Li1 | N1B | 77.2(5) |

**Table S8**. Key bond lengths and angles for complex **7**.

| Key bond lengths | | |  | Key bond angles | | | |
| --- | --- | --- | --- | --- | --- | --- | --- |
| **Atom** | **Atom** | **Length/Å** |  | **Atom** | **Atom** | **Atom** | **Angle/** **˚** |
| Li1 | N1A | 2.213(6) |  | N1A | Li1 | N2A | 83.6(2) |
| Li1 | N2A | 2.218(6) |  | N1A | Li1 | N3A | 81.09(18) |
| Li1 | N3A | 2.227(5) |  | N2A | Li1 | N3A | 111.9(2) |
| Li1 | N4A | 2.201(6) |  | N4A | Li1 | N1A | 81.5(2) |
| Li1 | S1 | 2.606(2) |  | N4A | Li1 | N2A | 123.0(2) |
| C13 | C14 | 1.3537(18) |  | N4A | Li1 | N3A | 119.37(19) |
| C13 | N5 | 1.4608(15) |  | C13 | S1 | Li1 | 138.41(7) |
| C13 | S1 | 1.7310(12) |  | C14 | C13 | N5 | 115.08(11) |
| N5 | Si1 | 1.7600(11) |  | C14 | C13 | S1 | 129.11(10) |
| N5 | C21 | 1.4025(16) |  | N5 | C13 | S1 | 115.77(9) |
|  |  |  |  | C13 | C14 | C15A | 127.6(3) |
|  |  |  |  | C13 | N5 | Si1 | 117.23(8) |
|  |  |  |  | C21 | N5 | C13 | 116.76(10) |
|  |  |  |  | C21 | N5 | Si1 | 125.29(8) |
|  | | | | | | | |
| Li1 | N1B | 2.201(12) |  | N1B | Li1 | N2B | 78.5(4) |
| Li1 | N2B | 2.265(11) |  | N1B | Li1 | N4B | 81.1(4) |
| Li1 | N3B | 2.117(11) |  | N3B | Li1 | N1B | 84.4(4) |
| Li1 | N4B | 2.203(10) |  | N3B | Li1 | N2B | 125.5(4) |
|  |  |  |  | N3B | Li1 | N4B | 119.8(3) |
|  |  |  |  | N4B | Li1 | N2B | 108.1(4) |
|  |  |  |  | C13 | C14 | C15B | 139.1(6) |

**Table S9**. Key bond lengths and angles for complex **8**.

| Key bond lengths | | |  | Key bond angles | | | |
| --- | --- | --- | --- | --- | --- | --- | --- |
| **Atom** | **Atom** | **Length/Å** |  | **Atom** | **Atom** | **Atom** | **Angle/** **˚** |
| Na1 | N1 | 2.5569(17) |  | N2 | Na1 | N1 | 72.80(6) |
| Na1 | N2 | 2.4594(18) |  | N2 | Na1 | N3 | 110.77(7) |
| Na1 | N3 | 2.5531(17) |  | N2 | Na1 | N4 | 105.12(6) |
| Na1 | N4 | 2.4826(16) |  | N3 | Na1 | N1 | 70.47(5) |
| Na1 | S1 | 2.7939(8) |  | N4 | Na1 | N1 | 72.93(5) |
| S1 | C13 | 1.7345(17) |  | N4 | Na1 | N3 | 116.66(6) |
| C13 | C14 | 1.358(2) |  | C13 | S1 | Na1 | 124.34(6) |
| C13 | N5 | 1.465(2) |  | C14 | C13 | N5 | 113.87(15) |
| N5 | C21 | 1.397(2) |  | C14 | C13 | S1 | 128.53(13) |
| Si1 | N5 | 1.7533(14) |  | N5 | C13 | S1 | 117.53(12) |
|  |  |  |  | C13 | N5 | Si1 | 115.85(11) |
|  |  |  |  | C21 | N5 | C13 | 117.90(13) |
|  |  |  |  | C21 | N5 | Si1 | 125.63(11 |
|  |  |  |  | C13 | C14 | C15 | 132.29(16) |

**Table S10**. Key bond lengths and angles for complex **9**.

| Key bond lengths | | |  | Key bond angles | | | |
| --- | --- | --- | --- | --- | --- | --- | --- |
| **Atom** | **Atom** | **Length/Å** |  | **Atom** | **Atom** | **Atom** | **Angle/** **˚** |
| Na1 | N1 | 2.470(5) |  | N1 | Na1 | N2 | 114.06(18) |
| Na1 | N2 | 2.490(5) |  | N1 | Na1 | N4 | 73.44(16) |
| Na1 | N3 | 2.449(5) |  | N2 | Na1 | C4 | 27.06(17) |
| Na1 | N4 | 2.495(5) |  | N2 | Na1 | N4 | 73.71(16) |
| Na1 | S1 | 2.717(2) |  | N3 | Na1 | N1 | 108.64(17) |
| C13 | S1 | 1.761(5) |  | N3 | Na1 | N2 | 113.78(16) |
| C13 | C14 | 1.527(7) |  | N3 | Na1 | N4 | 72.99(17) |
| C13 | N5 | 1.269(7) |  | C13 | S1 | Na1 | 124.07(17) |
|  |  |  |  | N4 | Na1 | N2 | 107.78(5) |
|  |  |  |  | N4 | Na1 | N3 | 113.51(5) |
|  |  |  |  | C14 | C13 | S1 | 114.8(4) |
|  |  |  |  | N5 | C13 | C14 | 114.9(4) |
|  |  |  |  | N5 | C13 | S1 | 130.3(4) |

**Table S11**. Key bond lengths and angles for complex **10**.

| Key bond lengths | | |  | Key bond angles | | | |
| --- | --- | --- | --- | --- | --- | --- | --- |
| **Atom** | **Atom** | **Length/Å** |  | **Atom** | **Atom** | **Atom** | **Angle/** **˚** |
| Na1 | N1 | 2.5168(18) |  | N1 | Na1 | N3 | 109.03(6) |
| Na1 | N2 | 2.5077(16) |  | N1 | Na1 | N4 | 71.88(5) |
| Na1 | N3 | 2.5371(17) |  | N1 | Na1 | S1 | 100.67(4) |
| Na1 | N4 | 2.5483(16) |  | N2 | Na1 | N1 | 113.37(6) |
| Na1 | S1 | 2.7922(8) |  | N2 | Na1 | N3 | 109.96(6) |
| Si1 | S1 | 2.0709(7) |  | N2 | Na1 | N4 | 71.12(5) |
| C13 | C14 | 1.527(7) |  | N2 | Na1 | S1 | 96.27(4) |
| C13 | N5 | 1.269(7) |  | N3 | Na1 | N4 | 72.71(5) |
|  |  |  |  | N3 | Na1 | S1 | 127.01(5) |
|  |  |  |  | N4 | Na1 | S1 | 160.04(5) |
|  |  |  |  | Si1 | S1 | Na1 | 114.57(3) |

**Table S12**. Key bond lengths and angles for complex **11**.

| Key bond lengths | | |  | Key bond angles | | | |
| --- | --- | --- | --- | --- | --- | --- | --- |
| **Atom** | **Atom** | **Length/Å** |  | **Atom** | **Atom** | **Atom** | **Angle/** **˚** |
| Li1A | N1A | 2.301(4) |  | N2A | Li1A | N1A | 78.75(12) |
| Li1A | N2A | 2.225(4) |  | N3A | Li1A | N1A | 79.44(13) |
| Li1A | N3A | 2.185(4) |  | N3A | Li1A | N2A | 118.20(16) |
| Li1A | N4A | 2.172(4) |  | N4A | Li1A | N1A | 81.08(13) |
| Li1A | N5A | 1.998(4) |  | N4A | Li1A | N2A | 117.81(16) |
| C13A | N5A | 1.175(3) |  | N4A | Li1A | N3A | 114.68(16) |
| C13A | N6A | 1.278(3) |  | C13A | N5A | Li1A | 158.14(18) |
|  |  |  |  | N5A | C13A | N6A | 176.4(2) |
|  |  |  |  | N2A | Li1A | N1A | 78.75(12) |
|  |  |  |  |  |  |  |  |
| Li1B | N1B | 2.283(4) |  | N2B | Li1B | N1B | 79.00(12) |
| Li1B | N2B | 2.238(3) |  | N3B | Li1B | N1B | 81.29(13) |
| Li1B | N3B | 2.188(4) |  | N3B | Li1B | N2B | 117.03(15) |
| Li1B | N4B | 2.230(4) |  | N3B | Li1B | N4B | 114.39(15) |
| Li1B | N5B | 1.995(4) |  | N4B | Li1B | N1B | 78.99(12) |
| C13B | N5B | 1.173(3) |  | N4B | Li1B | N2B | 119.26(15) |
| C13B | N6B | 1.270(3) |  | C13B | N5B | Li1B | 154.94(18) |
|  |  |  |  | N5B | C13B | N6B | 176.3(2) |

**Section 2**

**Computational Methods**

- 1. General computational procedure

All calculations in this work were completed using the B3LYP hybrid functional.^[[10]](#endnote-10),^^[[11]](#endnote-11)^ Initial AFIR (artificial force induced reaction) pathways were calculated using the GRRM (global reaction route mapping) V.23 software.^[[12]](#endnote-12)^ The calculated AFIR paths were used to explore the force-modulated potential energy surface (PES) using a force parameter, α, of 150 kJmol^-1^. The force-modulated PESs of interest were minimised using the locally updated planes (LUP) methodology, yielding estimate structures of minima and first order maxima. The estimate geometries identified (potential minima and first order maxima) were further optimised using the MIN and SADDLE options within GRRM respectively and confirmed to be either minima or 1^st^ order maxima (transition states) through frequency calculations. All vibrational frequencies of minima presented as real numbers, and first order maxima presented one imaginary (negative) frequency with the remainder being real. Intrinsic reaction coordinate (IRC) calculations were also completed for each of the first order saddle points to ensure that they were linked to their respective minima. All geometry optimisation and vibrational frequency calculations described above were completed using a 6-31G(d,p) basis set with both, corrections for the effects of benzene molecules (included using the IEFPCM solvent model), and dispersion corrections from Grimme’s D3 parameter with Becke-Johnson damping, as implemented in Gaussian V16 (Rev C.01).^[[13]](#endnote-13)^ This optimisation basis set will be henceforth referred to as basis set A (BS-A).

Further single point energy calculations were completed on these optimised geometries using a more robust basis set namely, 6-311++G(d,p), with the same solvent and dispersion corrections applied as the optimisations. This will henceforth be referred to as basis set B (BS-B). The correction to the free energy term obtained from the vibrational frequency calculation (BS-A) was added to the SCF energy of this calculation (BS-B) in an effort to improve the accuracy of the energy calculation without dramatically increasing the computational cost.

Molecular orbital diagrams were rendered based on the BS-B calculation using ChemCraft with a contour value of 0.03.^[[14]](#endnote-14)^ The topographic steric maps of **C**-Li and **C**–Na shown in Figure 8 were generated using the SambVca 2.1 web application.^[[15]](#endnote-15)^ The carbene atom defines the center of the xyz coordinate system. The nitrogen atoms of Me₆Tren coordinating to the metal define the xy plane, with the axial nitrogen atoms omitted in the case of **C**-Na. The z-axis passes through the carbene center, and the centroid of the nitrogen atoms used in the xy-plane definition. The oxo-carbene moiety was omitted; therefore, the plots reflect the steric map of the ligand portion. The blue and green zones indicate the less-hindered and more-hindered regions in the complexes, respectively.

- 1. Exploration into the formation of the Z-isomer of **3**

Experimentally, only the E isomer of **3** is observed, while the carbene intermediate (**C**-Li) could in theory lead to a mixture of both E/Z products through rotation about the C-C bond. In an attempt to understand why, a computational exploration into the formation of the Z product was undertaken.

**Figure S60:** Reaction pathway for the reaction of **2**Li with CO forming **3** through a 1,2-insertion reaction mechanism.

It is clear that observed product (**3**) is the more thermodynamically favored product when compared to the unobserved product (**3’**) likely due to increased steric clash. Moreover, the barrier to the 1,2-silyl migration and enolization step is much lower for the formation of **3** compared to that of **3’** (ΔΔ*G*^‡^ = 7.96). Despite this, the energies of prior carbene structures follow the opposite trend with **C**-Li being 1.01 kcalmol^-1^ higher in energy than **C’**-Li. A scan about the dihedral angle that allows interconversion is shown in Figure S61, highlighting that a small barrier (Δ*G*^‡^ = 2.64 kcalmol^-1^) exists between the two structures suggesting rapid interconversion between the two. As rapid conversion exists, the lower 1,2-silyl migration barrier will be followed, yielding product **3**.


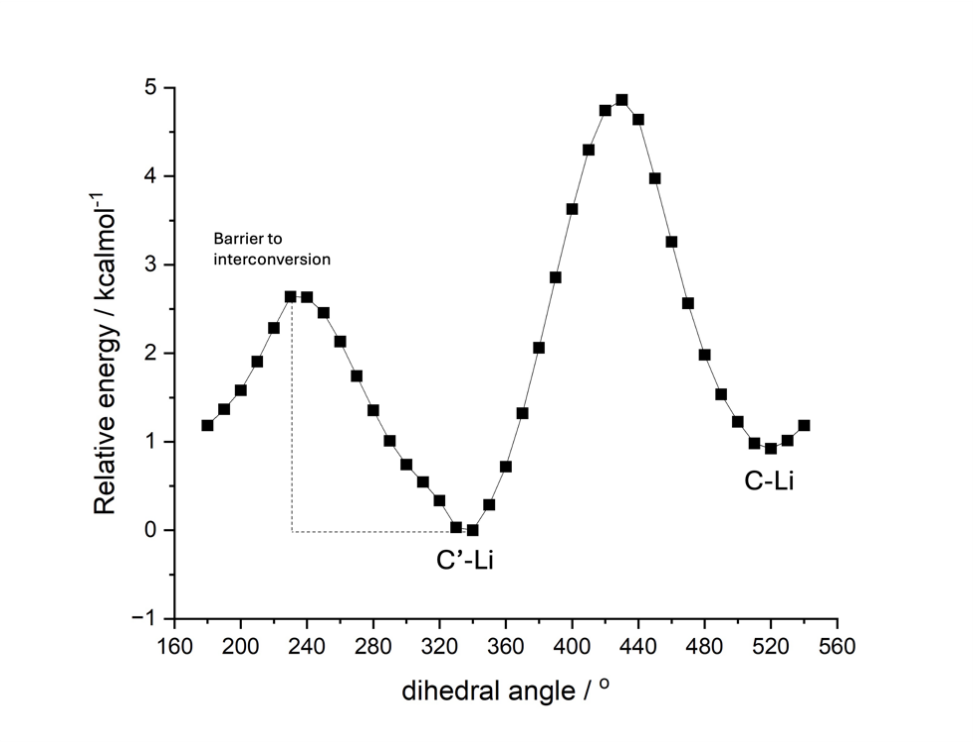


**Figure S61:** Relaxed dihedral scan of O-C-C-H highlighted in red (calculated using B3LYP/6-31G(d,p)) with the minima of both **C**-Li and **C’**-Li labelled.

- 1. 1,1 vs. 1,2-insertion of CO into **2**Li

**Figure S62:** Reaction pathway for the 1,2-insertion reaction between CO and **2**Li forming product **3**.

The barrier to 1,2-insertion (24.55 kcalmol^-1^) is much larger than the barrier to 1,1-insertion (17.15 kcalmol^-1^ – shown in Figure 6 in the main text), with both reactions resulting in the same product (**3**) suggesting that the reaction is more likely to proceed via a 1,1-insertion mechanism.

2.4 Summary of energies for the reaction pathways to each of the products **3**, **4** and **5**

A summary of the relevant energies for each of the reactions studied in this work are outlined below. The energies of all the structures in the reaction pathway diagrams (represented as relative to the initial starting material) are shown in the Tables below, all values given in the units of kcalmol^-1^. The values used to generate the reaction pathway diagrams shown in the main text are given in bold.

**Table S13:** Energies of the various structures in the reaction between **2**Li and CO to form **3** (both 1,1 and 1,2-insertion routes), with all energies being presented in kcalmol^-1^ relative to **2**Li.

|  | SCF E (BS-A) | G (BS-A) | SCF E (BS-B) | **E (BS-B) + ΔG_corr_(BS-A)** |
| --- | --- | --- | --- | --- |
| **2**Li + CO | 0.00 | 0.00 | 0.00 | **0.00** |
| **A**-Li | -1.62 | 7.80 | 1.86 | **11.29** |
| TS(**A**-**B**)-Li | 1.73 | 14.00 | 4.88 | **17.15** |
| **B**-Li | -5.08 | 5.93 | -0.52 | **10.48** |
| TS(**B-C**)-Li | -5.09 | 6.48 | 0.54 | **12.11** |
| **C**-Li | -5.88 | 4.86 | 0.11 | **10.85** |
| TS(**C**-**3**)-Li | -4.46 | 9.72 | 2.37 | **16.54** |
| **3** | -47.17 | -29.92 | -41.69 | **-24.43** |
| **A’**-Li | -0.61 | 13.10 | 1.95 | **15.63** |
| TS(**A’**-**C**)-Li | 7.05 | 20.90 | 10.73 | **24.54** |

**Table S14:** Energies of the various structures in the reaction between **2**Na and CO to form **5**, with all energies being presented in kcalmol^-1^ relative to **2**Na.

|  | SCF E (BS-A) | G (BS-A) | SCF E (BS-B) | **E (BS-B) + ΔG_corr_(BS-A)** |
| --- | --- | --- | --- | --- |
| **2**Na + CO | 0.00 | 0.00 | 0.00 | **0.00** |
| **A**-Na | -2.50 | 5.67 | 0.67 | **8.85** |
| TS(**A**-**B**)-Na | -2.64 | 6.72 | 0.86 | **10.22** |
| **B**-Na | -8.58 | 0.33 | -3.28 | **5.62** |
| TS(**B**-**C**)-Na | -7.62 | 5.68 | -2.47 | **10.84** |
| **C**-Na | -7.78 | 2.35 | -2.65 | **7.47** |
| TS(**C**-**5**)-Na | 2.78 | 12.47 | 8.33 | **18.02** |
| **5** | -42.18 | -31.00 | -37.73 | **-26.55** |

**Table S15:** Energies of the various structures in the reaction between **2**Na and CO to form **4**, starting from **C**-Na as the initial steps are shared with the data in Table S13. All energies are presented in kcalmol^-1^ relative to **2**Na.

|  | SCF E (BS-A) | G (BS-A) | SCF E (BS-B) | **E (BS-B) + ΔG_corr_(BS-A)** |
| --- | --- | --- | --- | --- |
| TS(**C**-**D**)-Na | -15.30 | 4.97 | -7.02 | **13.25** |
| **D**-Na | -36.15 | -13.07 | -28.89 | **-5.82** |
| TS(**D**-**E**)-Na | -36.66 | 4.11 | -24.33 | **16.44** |
| **E**-Na | -84.43 | -39.23 | -69.88 | **-24.67** |
| TS(**E**-**F**)-Na | -78.71 | -35.15 | -62.85 | **-19.29** |
| **F**-Na | -88.64 | -42.30 | -75.84 | **-29.50** |
| TS(**F**-**G**)-Na | -85.92 | -36.52 | -71.94 | **-22.55** |
| **G**-Na | -91.04 | -64.99 | -82.68 | **-56.63** |
| TS(**G**-**4**)-Na | -68.38 | -39.70 | -58.40 | **-29.72** |
| **4** | -112.53 | -84.40 | -103.14 | **-75.01** |

**Table S16:** Energies of the Li containing structures used to compare to the reaction of **C**-Na and CO, energies given relative to the energy of **C**-Li as is shown in Figure 7.

|  | SCF E (BS-A) | G (BS-A) | SCF E (BS-B) | **E (BS-B) + ΔG_corr_(BS-A)** |
| --- | --- | --- | --- | --- |
| TS(**C**-**D**)-Li | -5.46 | 8.16 | -3.72 | **9.90** |
| **D**-Li | -29.84 | -15.84 | -29.20 | **-15.20** |

**Table S17:** Energies of the structures presented in Figure S20 for the formation of the hypothetical Z isomer of **3** (**3’**).

|  | SCF E (BS-A) | G (BS-A) | SCF E (BS-B) | **E (BS-B) + ΔG_corr_(BS-A)** |
| --- | --- | --- | --- | --- |
| **C’**-Li | -8.04 | 4.02 | -2.20 | **9.84** |
| TS(**C’**-**3’**)-Li | 7.90 | 19.13 | 13.30 | **24.50** |
| **3’** | -35.01 | -22.42 | -28.05 | **-17.68** |

**Table S18:** Energies of reaction pathway steps for the formation of product **7**, from the reaction between **2**Li and PhNCS. All energies are presented in kcalmol^-1^ relative to **2**Li.

|  | SCF E (BS-A) | G (BS-A) | SCF E (BS-B) | **E (BS-B) + ΔG_corr_(BS-A)** |
| --- | --- | --- | --- | --- |
| **2**Li + PhNCS | 0.00 | 0.00 | 0.00 | **0.00** |
| TS(**2**-**E**)-Li | 9.31 | 17.14 | 11.32 | **28.46** |
| **E**-Li | -47.70 | 24.22 | -45.34 | **-21.13** |
| TS(**E**-**7**)-Li | -34.06 | 18.94 | -30.60 | **-11.66** |
| **7** | -56.62 | 19.66 | -53.19 | **-33.53** |

**Table S19:** Energies of reaction pathway steps for the formation of product **8**, from the reaction between **2**Na and PhNCS. All energies are presented in kcalmol^-1^ relative to **2**Na.

|  | SCF E (BS-A) | G (BS-A) | SCF E (BS-B) | **E (BS-B) + ΔG_corr_(BS-A)** |
| --- | --- | --- | --- | --- |
| **2**Na + PhNCS | 0.00 | 0.00 | 0.00 | **0.00** |
| TS(**2**-**H)**-Na | 1.51 | 16.44 | 1.84 | **18.28** |
| **H**-Na | -44.18 | 23.04 | -41.01 | **-17.97** |
| TS(**H**-**8**)-Na | -33.45 | 20.54 | -29.97 | **-9.43** |
| **8** | -59.15 | 18.56 | -56.20 | **-37.64** |

**Table S20:** Energies of reaction pathway steps for the formation of product **9**, from the reaction between **2**Na and *t*BuNCS. All energies are presented in kcalmol^-1^ relative to **2**Na.

|  | SCF E (BS-A) | G (BS-A) | SCF E (BS-B) | **E (BS-B) + ΔG_corr_(BS-A)** |
| --- | --- | --- | --- | --- |
| **2**Na +  *t*BuNCS | 0.00 | 0.00 | 0.00 | **0.00** |
| TS(**2**-**9)**-Na | 6.65 | 15.24 | 6.88 | **22.12** |
| **9** | -41.16 | 24.52 | -38.11 | **-13.59** |

2.5 Coordinates of optimised structures:

The following pages summarise the cartesian atomic coordinates of the optimised structures (both minima and transition states) rounded to 5 decimal places, alongside SCF E calculated using BS-A, given in Hartrees, as well as the two lowest energy frequencies (denoted ω_1_ and ω_2_ respectively, given in cm^-1^).

**2**Li + CO → **3** (including 1,2-insertion)

**2**Li

SCF E (BS-A) = -1381.734909462160

$\omega$1 = 22.20734873

$\omega$2 = 27.33026264

Si -0.55196 2.74712 0.04342

N 1.40483 -1.44112 -0.24736

N -0.42373 -2.02668 1.98442

N -1.21674 -1.81488 -1.44283

N 4.37325 0.46332 -1.66215

C 1.86996 -2.04383 1.01166

H 2.21384 -1.22978 1.65619

H 2.73340 -2.71010 0.85557

C 0.77856 -2.83587 1.72587

H 0.48700 -3.69159 1.11275

H 1.19255 -3.24955 2.66135

C 1.21031 -2.41593 -1.34168

H 1.86280 -3.29311 -1.22151

H 1.51226 -1.93755 -2.27619

C -0.23709 -2.90578 -1.48344

H -0.31354 -3.48492 -2.42165

H -0.48713 -3.59345 -0.67207

C 2.24338 -0.29866 -0.65039

H 2.14621 0.46004 0.12875

H 1.81620 0.12922 -1.55997

C 3.72076 -0.61284 -0.92109

H 4.24479 -0.82879 0.03136

H 3.78859 -1.52121 -1.53094

C -0.20059 -1.10833 3.11077

H 0.07583 -1.65236 4.02918

H -1.11135 -0.53919 3.30002

H 0.58976 -0.39421 2.87208

C -1.57458 -2.88977 2.27575

H -1.72460 -3.59255 1.45306

H -2.47282 -2.27690 2.36746

H -1.43781 -3.46856 3.20405

C -2.58360 -2.34836 -1.36985

H -2.83531 -2.94969 -2.25958

H -3.29153 -1.52427 -1.28293

H -2.68364 -2.98167 -0.48594

C -1.09743 -0.92262 -2.60311

H -0.12282 -0.43295 -2.61474

H -1.86180 -0.14892 -2.52459

H -1.22999 -1.46683 -3.55333

C 4.39963 1.71689 -0.91442

H 3.38695 2.08152 -0.72998

H 4.92147 2.47642 -1.50296

H 4.91670 1.62504 0.06040

C 5.71907 0.07235 -2.06104

H 6.38905 -0.11931 -1.20101

H 6.16810 0.86426 -2.66774

H 5.67663 -0.83862 -2.66610

C -1.39750 1.25057 0.68621

H -1.19293 1.11544 1.75433

C -2.75387 0.88673 0.33939

C -3.36251 1.22364 -0.90760

H -2.80694 1.83660 -1.61144

C -4.64312 0.80245 -1.25070

H -5.05291 1.09384 -2.21561

C -5.40794 0.01693 -0.38041

H -6.40541 -0.31221 -0.65320

C -4.85322 -0.31028 0.86287

H -5.43069 -0.89645 1.57510

C -3.57721 0.11497 1.21355

H -3.19069 -0.12750 2.20118

C 0.02977 2.65401 -1.76975

H 0.74247 1.84082 -1.93278

H 0.52071 3.58870 -2.06623

H -0.80920 2.49501 -2.45595

C 0.98433 3.07333 1.11898

H 0.68769 3.32317 2.14416

H 1.57649 3.91094 0.73339

H 1.64299 2.19981 1.17509

C -1.61805 4.32567 0.12404

H -2.56006 4.18106 -0.41641

H -1.11254 5.19708 -0.30936

H -1.87417 4.56439 1.16245

Li -0.59917 -0.72605 0.23426

CO

SCF E (BS-A) = -113.310492290618

$\omega$1 = 2207.09440831

$\omega$2 = N/A

C -0.92357 1.87191 0.01274

O 0.19183 1.87191 0.01274

**A**-Li

SCF E (BS-A) = -1495.047983799757

$\omega$1 = 10.56916875

$\omega$2 = 23.51692885

Si 1.88508 -2.00561 -1.71565

N -1.93287 0.93269 0.11066

N 0.20330 2.55109 1.40249

N -0.27317 1.93310 -2.09086

N -4.48270 -1.88280 -0.06917

C -2.05959 1.50425 1.46193

H -1.73784 0.73305 2.16792

H -3.10593 1.75008 1.70612

C -1.22747 2.76547 1.66227

H -1.58729 3.54916 0.99047

H -1.39649 3.13931 2.68683

C -2.50274 1.78571 -0.95148

H -3.30002 2.43776 -0.56494

H -2.97777 1.13592 -1.69005

C -1.47367 2.66642 -1.66756

H -1.97522 3.14081 -2.53089

H -1.14178 3.47537 -1.01436

C -2.47247 -0.43867 0.06388

H -1.89203 -1.03056 0.77142

H -2.27539 -0.85162 -0.92956

C -3.97343 -0.58074 0.35521

H -4.17185 -0.39811 1.42972

H -4.52602 0.18486 -0.20118

C 0.84737 1.88667 2.54608

H 0.81559 2.52035 3.44841

H 1.88728 1.66157 2.30467

H 0.36492 0.93500 2.76025

C 0.86962 3.83168 1.15504

H 0.43214 4.32542 0.28323

H 1.92987 3.65993 0.95707

H 0.78932 4.51661 2.01594

C 0.70514 2.86839 -2.65578

H 0.31471 3.39253 -3.54376

H 1.60514 2.32298 -2.94759

H 0.98042 3.61441 -1.90662

C -0.58998 0.89549 -3.07973

H -1.21650 0.12240 -2.63519

H 0.33490 0.41959 -3.40548

H -1.10672 1.31097 -3.96130

C -3.88021 -2.98262 0.67830

H -2.80302 -3.02654 0.50255

H -4.30904 -3.92903 0.33746

H -4.04952 -2.90339 1.76956

C -5.93551 -1.92674 0.03500

H -6.30100 -1.80636 1.07292

H -6.30315 -2.88569 -0.34217

H -6.37517 -1.12974 -0.57276

C 0.97978 -1.21841 -0.31415

H -0.07105 -1.52059 -0.37698

C 1.46232 -1.26458 1.04973

C 2.81940 -0.99984 1.40214

H 3.53833 -0.81834 0.60898

C 3.25743 -0.98580 2.72261

H 4.30530 -0.77839 2.92886

C 2.37792 -1.23629 3.78119

H 2.72319 -1.22279 4.81000

C 1.04618 -1.53572 3.47100

H 0.34427 -1.76953 4.26903

C 0.60405 -1.55166 2.15302

H -0.42793 -1.82570 1.94803

C 3.04318 -0.90898 -2.75991

H 2.54554 -0.00499 -3.12827

H 3.40139 -1.46882 -3.63213

H 3.92328 -0.58852 -2.19398

C 0.59290 -2.70778 -2.92363

H -0.02055 -3.46837 -2.42764

H 1.07147 -3.17884 -3.78974

H -0.08502 -1.93542 -3.30053

C 2.97018 -3.46835 -1.16110

H 3.68846 -3.16238 -0.39353

H 3.53330 -3.90046 -1.99670

H 2.35002 -4.25944 -0.72508

Li 0.29613 0.94466 -0.26097

O 3.64368 1.56766 -0.74154

C 2.55386 1.47144 -0.41943

TS(**A**-**B**)-Li

SCF E (BS-A) = -1495.042650794133

$\omega$1 = -201.22848294

$\omega$2 = 21.44905504

Si 2.23223 0.79427 2.16364

N -2.32146 -0.02652 -0.44620

N -1.10027 -2.36314 -1.73204

N -2.06271 -2.01986 1.65074

N -2.50365 3.75884 -0.09705

C -2.51303 -0.31906 -1.87937

H -1.75952 0.24921 -2.43068

H -3.49479 0.02488 -2.24013

C -2.37056 -1.80324 -2.21542

H -3.18573 -2.36718 -1.75396

H -2.48098 -1.92981 -3.30603

C -3.48033 -0.38894 0.39856

H -4.42218 -0.34090 -0.16665

H -3.56408 0.35718 1.19202

C -3.37557 -1.77928 1.04096

H -4.19422 -1.88280 1.77515

H -3.52811 -2.55801 0.28936

C -1.88543 1.36662 -0.22943

H -0.94790 1.49296 -0.77325

H -1.65083 1.49533 0.83145

C -2.88431 2.45671 -0.63739

H -2.98512 2.48434 -1.74073

H -3.87418 2.21015 -0.23689

C 0.03947 -1.91027 -2.54674

H -0.06537 -2.22339 -3.59829

H 0.96149 -2.32677 -2.13928

H 0.14047 -0.82581 -2.51003

C -1.14020 -3.82860 -1.71115

H -1.98359 -4.16837 -1.10416

H -0.21689 -4.20646 -1.26600

H -1.24649 -4.25793 -2.72065

C -1.90800 -3.42111 2.05327

H -2.61398 -3.70650 2.84996

H -0.88776 -3.57988 2.40969

H -2.07386 -4.07317 1.19262

C -1.80144 -1.13754 2.79306

H -1.84177 -0.09151 2.48535

H -0.79640 -1.33511 3.17196

H -2.52658 -1.28974 3.60923

C -1.22968 4.23247 -0.63123

H -0.41400 3.56488 -0.34411

H -1.00905 5.21897 -0.21477

H -1.23261 4.31998 -1.73481

C -3.55586 4.74031 -0.32808

H -3.74627 4.92924 -1.40193

H -3.28097 5.69121 0.13766

H -4.49031 4.39562 0.12556

C 1.61460 0.23281 0.49379

H 0.62764 0.67483 0.32884

C 2.45451 0.36598 -0.69204

C 3.76095 -0.18167 -0.74253

H 4.15431 -0.67561 0.13913

C 4.52927 -0.12815 -1.90206

H 5.52575 -0.56358 -1.89986

C 4.03830 0.47373 -3.06459

H 4.64413 0.51827 -3.96450

C 2.75333 1.02485 -3.04102

H 2.35306 1.50856 -3.92926

C 1.98315 0.97144 -1.88325

H 0.99501 1.42795 -1.88055

C 2.63185 -0.63666 3.34313

H 1.74170 -1.24032 3.55270

H 3.01861 -0.26290 4.29799

H 3.37591 -1.30908 2.90646

C 0.85678 1.82631 2.97254

H 0.61565 2.70600 2.36528

H 1.14564 2.17618 3.97010

H -0.06230 1.24106 3.08416

C 3.77981 1.88176 2.03875

H 4.59743 1.35534 1.53765

H 4.13041 2.18862 3.03091

H 3.57196 2.78587 1.45633

Li -0.77393 -1.48632 0.13944

O 2.23198 -2.37764 0.67956

C 1.22680 -1.74801 0.61531

**B**-Li

SCF E (BS-A) = -1495.053490904517

$\omega$1 = 10.67547316

$\omega$2 = 17.53122055

Si 3.37700 -0.26728 -1.60698

N -2.39981 -0.43426 0.16814

N -1.84366 2.13208 1.46471

N -2.47564 1.53527 -1.96130

N -1.45493 -4.10807 -0.05952

C -2.79316 -0.16718 1.56050

H -1.99282 -0.53952 2.20498

H -3.70690 -0.71325 1.84399

C -3.01113 1.32013 1.84019

H -3.87118 1.68120 1.27025

H -3.26488 1.44807 2.90700

C -3.51561 -0.41710 -0.79719

H -4.46373 -0.71812 -0.32723

H -3.30612 -1.16371 -1.56682

C -3.72749 0.94503 -1.47519

H -4.46363 0.81538 -2.28922

H -4.16616 1.65361 -0.76760

C -1.54448 -1.62707 0.04285

H -0.65435 -1.44219 0.64783

H -1.20839 -1.69682 -0.99583

C -2.20438 -2.95592 0.43495

H -2.33866 -2.99825 1.53472

H -3.20692 -3.00340 -0.00585

C -0.73248 1.94670 2.41102

H -1.02475 2.22985 3.43631

H 0.11370 2.55789 2.09897

H -0.38797 0.91258 2.41285

C -2.19687 3.55250 1.39153

H -3.02167 3.69407 0.68869

H -1.33541 4.12205 1.03401

H -2.50296 3.95945 2.36968

C -2.66847 2.92771 -2.37372

H -3.35059 3.01909 -3.23553

H -1.70164 3.35677 -2.64680

H -3.08276 3.50668 -1.54467

C -1.88183 0.76820 -3.06464

H -1.66395 -0.25194 -2.74578

H -0.93416 1.23065 -3.34305

H -2.54953 0.73068 -3.94186

C -0.10617 -4.17489 0.49271

H 0.48720 -3.31697 0.16787

H 0.39143 -5.07609 0.12423

H -0.09317 -4.20601 1.59966

C -2.18170 -5.34396 0.20082

H -2.32442 -5.54535 1.27989

H -1.63840 -6.18963 -0.23110

H -3.16979 -5.29787 -0.26751

C 1.83409 -0.06594 -0.51671

H 1.28318 -1.01525 -0.60131

C 2.13440 0.15915 0.93151

C 2.62231 1.40627 1.36824

H 2.81179 2.17890 0.63106

C 2.84584 1.66276 2.71964

H 3.22356 2.63460 3.02602

C 2.57759 0.68485 3.68078

H 2.74900 0.88635 4.73384

C 2.09142 -0.55755 3.26816

H 1.88344 -1.33164 4.00213

C 1.87321 -0.81273 1.91383

H 1.50468 -1.78728 1.60607

C 4.54517 1.21368 -1.52635

H 4.02078 2.11304 -1.85911

H 5.41983 1.05804 -2.16835

H 4.90475 1.38166 -0.50631

C 2.82024 -0.56535 -3.38997

H 2.13519 -1.41933 -3.44707

H 3.66946 -0.77536 -4.04985

H 2.29456 0.31650 -3.76455

C 4.31660 -1.80461 -1.00782

H 4.62160 -1.69005 0.03802

H 5.21878 -1.98736 -1.60322

H 3.68915 -2.70126 -1.07201

Li -1.17689 1.25917 -0.35292

O 1.28226 1.89314 -1.81277

C 0.80716 1.07765 -1.00425

TS(**B**-**C**)-Li

SCF E (BS-A) = -1495.053505874089

$\omega$1 = -100.19198478

$\omega$2 = 11.65395235

Si 3.49419 1.26931 -1.26749

N -2.52226 -0.29083 -0.20011

N -1.68972 1.02774 2.23763

N -2.54527 2.56864 -0.83258

N -1.44790 -3.68496 -1.55499

C -3.17187 -0.61468 1.08152

H -2.70575 -1.51869 1.48082

H -4.23747 -0.85374 0.94793

C -3.05560 0.50583 2.12616

H -3.70050 1.34430 1.84869

H -3.43016 0.12436 3.09355

C -3.41813 0.26141 -1.22216

H -4.36734 -0.29498 -1.29356

H -2.91915 0.15336 -2.18886

C -3.74967 1.73712 -0.98720

H -4.38392 2.09718 -1.81613

H -4.34354 1.83769 -0.07470

C -1.65648 -1.36735 -0.71051

H -0.93290 -1.60460 0.07217

H -1.06538 -0.96180 -1.53521

C -2.38912 -2.63123 -1.17845

H -3.09967 -2.97824 -0.40213

H -2.99060 -2.38441 -2.05983

C -0.75547 0.04393 2.79803

H -1.05188 -0.26950 3.81366

H 0.24254 0.47913 2.83071

H -0.69537 -0.83944 2.16178

C -1.65041 2.25859 3.03047

H -2.32922 2.99812 2.59831

H -0.63723 2.66532 3.00237

H -1.94060 2.09189 4.08152

C -2.87949 3.87117 -0.25069

H -3.53839 4.46689 -0.90412

H -1.96092 4.43662 -0.07564

H -3.38586 3.72869 0.70734

C -1.84625 2.75720 -2.11247

H -1.51571 1.79644 -2.50818

H -0.95285 3.36169 -1.94577

H -2.48790 3.25781 -2.85732

C -0.87939 -4.34963 -0.38742

H -0.36591 -3.63282 0.25531

H -0.14012 -5.08843 -0.71147

H -1.64462 -4.87406 0.21757

C -2.06371 -4.66023 -2.44548

H -2.91136 -5.20004 -1.98078

H -1.31946 -5.40360 -2.74721

H -2.43164 -4.16163 -3.34719

C 1.98892 0.26800 -0.6808

H 1.64611 -0.29114 -1.56279

C 2.28155 -0.73252 0.39959

C 2.66656 -0.32890 1.69291

H 2.78024 0.72914 1.89489

C 2.86511 -1.26073 2.70988

H 3.16168 -0.91894 3.69805

C 2.68301 -2.62511 2.46992

H 2.84327 -3.35028 3.26212

C 2.29026 -3.04226 1.19676

H 2.13896 -4.09870 0.99212

C 2.08647 -2.10799 0.18037

H 1.76460 -2.44363 -0.80195

C 4.38769 2.16746 0.13053

H 3.67227 2.78568 0.67954

H 5.18099 2.81164 -0.26585

H 4.84410 1.46492 0.83414

C 2.88373 2.54324 -2.52803

H 2.31919 2.06179 -3.33434

H 3.71725 3.09020 -2.98262

H 2.22334 3.26965 -2.04430

C 4.70159 0.08406 -2.11947

H 5.02802 -0.69711 -1.42452

H 5.59398 0.60751 -2.48138

H 4.23310 -0.41080 -2.97751

Li -1.18603 1.36211 0.23682

O 1.04181 2.09581 0.56488

C 0.76571 1.16796 -0.22996

**C**-Li

SCF E (BS-A) = -1495.054773535292

$\omega$1 = 12.08335347

$\omega$2 = 23.01700837

Si 3.56892 1.22826 -1.20349

N -2.59551 -0.16496 -0.13280

N -1.64622 1.12435 2.26744

N -2.31998 2.68626 -0.83733

N -1.91230 -3.61483 -1.60944

C -3.28491 -0.38473 1.14815

H -2.92174 -1.32309 1.57390

H -4.36932 -0.51291 1.01021

C -3.05639 0.74049 2.16933

H -3.61485 1.63387 1.87664

H -3.46498 0.41711 3.14447

C -3.41474 0.46284 -1.17353

H -4.41711 0.00886 -1.25063

H -2.91740 0.28841 -2.13121

C -3.59810 1.96810 -0.96587

H -4.20243 2.37060 -1.79776

H -4.17298 2.14310 -0.05264

C -1.84986 -1.33984 -0.61250

H -1.14568 -1.61759 0.17507

H -1.23226 -1.02232 -1.45568

C -2.71055 -2.54063 -1.02212

H -3.30160 -2.90192 -0.15544

H -3.43378 -2.21695 -1.77925

C -0.81020 0.06101 2.83462

H -1.12510 -0.20894 3.85741

H 0.22676 0.39495 2.85231

H -0.84649 -0.83257 2.21079

C -1.47652 2.35506 3.04036

H -2.08409 3.15242 2.60533

H -0.42835 2.65945 2.99644

H -1.76836 2.23405 4.09752

C -2.52499 4.01681 -0.25938

H -3.14106 4.66541 -0.90458

H -1.55624 4.49933 -0.10729

H -3.02229 3.92701 0.70959

C -1.63465 2.80819 -2.13239

H -1.41290 1.82083 -2.53753

H -0.68056 3.31804 -1.98465

H -2.23893 3.37717 -2.85940

C -1.00617 -4.22844 -0.64362

H -0.27565 -3.50578 -0.27527

H -0.45212 -5.03559 -1.13189

H -1.53717 -4.65978 0.22776

C -2.77069 -4.62298 -2.21662

H -3.43004 -5.13069 -1.48578

H -2.15647 -5.38767 -2.70188

H -3.40522 -4.16079 -2.97922

C 2.09587 0.10015 -0.77532

H 1.88762 -0.45804 -1.69822

C 2.37933 -0.90822 0.30394

C 2.60436 -0.52297 1.63898

H 2.60307 0.53204 1.88272

C 2.79535 -1.47426 2.63990

H 2.96729 -1.14755 3.66211

C 2.76626 -2.83828 2.34007

H 2.91907 -3.57720 3.12092

C 2.53655 -3.23822 1.02239

H 2.50581 -4.29485 0.77043

C 2.34163 -2.28522 0.02224

H 2.15631 -2.60724 -0.99901

C 4.26892 2.14464 0.28943

H 3.47839 2.71842 0.78062

H 5.06261 2.83440 -0.01996

H 4.69249 1.45126 1.02199

C 2.98298 2.48739 -2.48950

H 2.53690 1.98681 -3.35585

H 3.81283 3.10560 -2.84975

H 2.22576 3.15211 -2.06214

C 4.92665 0.15157 -1.96605

H 5.24929 -0.62102 -1.26008

H 5.80545 0.74660 -2.23950

H 4.57019 -0.35351 -2.87060

Li -1.04480 1.38703 0.25147

O 0.88984 1.75284 0.47354

C 0.78098 0.88430 -0.43374

TS(**C**-**3**)-Li

SCF E (BS-A) = -1495.052504169044

$\omega$1 = -232.00488464

$\omega$2 = 17.41405922

Si -3.03237 2.07883 -0.71356

N 3.07665 -0.19116 -0.37252

N 1.25867 -1.32981 1.60870

N 1.28328 -1.89469 -2.00887

N 2.55409 2.61005 0.98656

C 3.50118 -0.41718 1.02169

H 3.32877 0.51493 1.55805

H 4.57856 -0.64771 1.08486

C 2.71084 -1.54213 1.68767

H 2.92827 -2.50148 1.20642

H 3.04674 -1.63694 2.73567

C 3.57195 -1.24592 -1.27665

H 3.67298 -2.17535 -0.71250

H 4.57829 -1.00870 -1.65755

C 2.62097 -1.46200 -2.45088

H 2.49566 -0.51842 -2.99023

H 3.06074 -2.18194 -3.16225

C 3.48514 1.14300 -0.85969

H 3.45337 1.11733 -1.95253

H 4.53673 1.33945 -0.59107

C 2.56350 2.28972 -0.44364

H 2.85517 3.17792 -1.04048

H 1.54483 2.01284 -0.72839

C 0.83602 -0.19869 2.45113

H 1.09914 -0.36759 3.50958

H -0.24484 -0.08353 2.36967

H 1.30687 0.72498 2.10958

C 0.53837 -2.54484 2.00302

H 0.81925 -3.37105 1.34494

H -0.53415 -2.37806 1.91010

H 0.76164 -2.83799 3.04298

C 0.29348 -1.63899 -3.06296

H -0.67954 -2.01706 -2.74511

H 0.56209 -2.13365 -4.01081

H 0.20398 -0.56311 -3.22594

C 1.26405 -3.31139 -1.63220

H 1.99877 -3.50807 -0.84896

H 1.48637 -3.97200 -2.48705

H 0.27719 -3.56490 -1.23770

C 3.80898 3.20562 1.42807

H 4.64344 2.51646 1.27097

H 3.75375 3.42449 2.49861

H 4.04104 4.14820 0.89684

C 1.43188 3.49691 1.29487

H 1.53808 4.48768 0.81346

H 1.37693 3.65377 2.37721

H 0.49501 3.04733 0.94836

C -2.19239 0.80580 0.62990

H -2.51641 1.41303 1.48280

C -2.48171 -0.64085 0.77318

C -2.25495 -1.52640 -0.29731

H -1.86432 -1.12358 -1.22208

C -2.53185 -2.88631 -0.17547

H -2.35941 -3.54888 -1.01989

C -3.04310 -3.40046 1.01975

H -3.25948 -4.46025 1.11374

C -3.28343 -2.53384 2.08765

H -3.69110 -2.91821 3.01845

C -3.00366 -1.17197 1.96355

H -3.19284 -0.50412 2.80039

C -3.16186 3.86079 -0.09257

H -3.19718 3.88464 1.0025

H -4.07437 4.34525 -0.46136

H -2.29498 4.44741 -0.40701

C -4.82721 1.39858 -0.66291

H -5.24309 1.44024 0.35052

H -4.85835 0.35109 -0.98326

H -5.49006 1.97252 -1.32231

C -2.57788 1.93322 -2.54943

H -1.67959 2.50642 -2.78936

H -3.40641 2.27852 -3.17897

H -2.38073 0.88603 -2.80304

Li 0.94096 -0.60759 -0.35429

O -0.24921 0.69694 -0.7271

C -1.08536 1.43107 -0.0874

**3**

SCF E (BS-A) = -1495.120577118284

$\omega$1 = 26.33626775

$\omega$2 = 30.91379821

Si 0.60445 3.40533 0.99570

O 0.10493 0.78045 0.58870

N 1.14018 -0.06997 -2.38828

N 1.16240 -2.33759 -0.45950

N 3.33296 -0.59472 0.38608

N -1.75471 -2.76570 1.90216

C 0.48905 -1.36637 -2.64789

H 0.44103 -1.58432 -3.72875

H -0.54142 -1.28785 -2.28911

C 1.19207 -2.51459 -1.92675

H 0.72702 -3.46938 -2.21656

H 2.23249 -2.57202 -2.25363

C 2.36273 -2.89248 0.18502

H 2.60253 -3.90042 -0.19290

H 2.15382 -3.00520 1.24955

C 3.58201 -1.99249 -0.00266

H 4.42833 -2.41921 0.56377

H 3.88430 -1.98689 -1.05420

C -0.07648 -2.90824 0.10268

H -0.02630 -4.01012 0.09328

H -0.90251 -2.62235 -0.55056

C -0.40746 -2.37052 1.49766

H 0.34287 -2.69782 2.24354

H -0.36058 -1.27908 1.45171

C 0.23792 1.03780 -2.73508

H -0.00983 1.04138 -3.80947

H 0.71954 1.98494 -2.48421

H -0.67872 0.96285 -2.15124

C 2.40372 0.07083 -3.11507

H 3.09779 -0.72757 -2.84621

H 2.86604 1.02485 -2.84979

H 2.25859 0.04598 -4.20826

C 4.46234 0.23987 -0.03848

H 5.40981 -0.07647 0.42843

H 4.27405 1.27918 0.23685

H 4.57609 0.18520 -1.12331

C 3.13374 -0.46184 1.83679

H 2.23229 -0.99057 2.15030

H 2.99592 0.59267 2.08042

H 3.99338 -0.85125 2.40717

C -1.80552 -4.13182 2.40219

H -1.43275 -4.83042 1.64814

H -2.84117 -4.40433 2.62651

H -1.20705 -4.27481 3.32358

C -2.31818 -1.82988 2.86780

H -1.74839 -1.79137 3.81606

H -3.34495 -2.12545 3.10430

H -2.34344 -0.82748 2.43491

C -0.49924 1.94656 0.49872

C -1.78739 2.17099 0.06873

H -2.11507 3.20799 0.05298

C -2.77220 1.20748 -0.39175

C -2.55917 -0.19054 -0.36996

H -1.61661 -0.54685 0.01157

C -3.53900 -1.08093 -0.80236

H -3.34910 -2.14827 -0.72599

C -4.76081 -0.61493 -1.29264

H -5.52466 -1.30993 -1.62869

C -4.98978 0.76476 -1.33172

H -5.93600 1.14664 -1.70689

C -4.01879 1.65571 -0.88679

H -4.21602 2.72486 -0.91610

C 1.18792 3.15030 2.77746

H 1.55609 2.12868 2.91102

H 0.35992 3.29548 3.47954

H 1.98938 3.84476 3.05265

C 2.11194 3.37093 -0.15348

H 1.85359 3.73950 -1.15213

H 2.45647 2.33824 -0.25656

H 2.94437 3.97443 0.22439

C -0.24134 5.09224 0.85227

H 0.44362 5.89955 1.13547

H -1.11762 5.15402 1.50583

H -0.57605 5.28295 -0.17271

Li 1.32491 -0.18347 -0.30211

**A’**-Li

SCF E (BS-A) = -1495.046366297088

$\omega$1 = 27.10919021

$\omega$2 = 35.46264319

Si 1.73252 -1.76791 -2.04932

N -1.88308 0.95637 0.27437

N 0.44897 2.17849 1.70029

N -0.20486 2.23031 -1.75430

N -4.72215 -1.51134 -0.27870

C -1.88308 1.26402 1.71543

H -1.57578 0.35249 2.23679

H -2.89116 1.52219 2.07821

C -0.95336 2.41177 2.08704

H -1.29772 3.32865 1.60213

H -1.03652 2.59157 3.17256

C -2.41061 2.04498 -0.57395

H -3.13534 2.67019 -0.03161

H -2.96330 1.58990 -1.39901

C -1.33064 2.96244 -1.15782

H -1.81171 3.62453 -1.90052

H -0.91768 3.60934 -0.38291

C -2.56254 -0.32604 0.00820

H -2.02225 -1.09194 0.56495

H -2.44237 -0.56334 -1.05292

C -4.05997 -0.36911 0.34657

H -4.20343 -0.36841 1.44502

H -4.54360 0.53765 -0.03379

C 1.10594 1.29038 2.67451

H 1.13313 1.74964 3.67705

H 2.12558 1.07926 2.35284

H 0.59119 0.33334 2.73472

C 1.16348 3.46003 1.67648

H 0.72221 4.12839 0.93301

H 2.20762 3.29496 1.41307

H 1.13031 3.96653 2.65576

C 0.84806 3.17327 -2.14816

H 0.49535 3.89183 -2.90654

H 1.69521 2.62191 -2.55949

H 1.19427 3.72838 -1.27494

C -0.62681 1.45132 -2.92539

H -1.32009 0.66243 -2.63166

H 0.24706 0.97396 -3.36899

H -1.11078 2.08536 -3.68727

C -4.23432 -2.78472 0.24222

H -3.17076 -2.90912 0.02664

H -4.76889 -3.60422 -0.24607

H -4.37920 -2.88437 1.33529

C -6.16834 -1.41501 -0.12732

H -6.49791 -1.44087 0.92911

H -6.64981 -2.24655 -0.65047

H -6.52606 -0.48022 -0.57016

C 0.79409 -1.29253 -0.53725

H -0.24868 -1.61010 -0.65190

C 1.29892 -1.59420 0.78695

C 2.67141 -1.43649 1.14219

H 3.37549 -1.12828 0.37469

C 3.13933 -1.66467 2.43112

H 4.19748 -1.52372 2.64110

C 2.27516 -2.07094 3.45482

H 2.64323 -2.24794 4.46034

C 0.92750 -2.27046 3.13446

H 0.23522 -2.61577 3.89990

C 0.45506 -2.04514 1.84543

H -0.59147 -2.24368 1.62470

C 2.97978 -0.48185 -2.70123

H 2.53156 0.51241 -2.79652

H 3.36707 -0.77440 -3.68447

H 3.84048 -0.38981 -2.02954

C 0.49122 -2.10998 -3.45050

H -0.13974 -2.96793 -3.19185

H 1.00484 -2.34805 -4.38892

H -0.17314 -1.26211 -3.64127

C 2.74731 -3.36551 -1.83424

H 3.42246 -3.29039 -0.97562

H 3.35135 -3.59457 -2.72023

H 2.08228 -4.21662 -1.64939

Li 0.24038 0.81620 -0.19928

C 4.12069 1.37325 0.05539

O 3.27986 2.01531 -0.36625

TS(**A’**-**C**)-Li

SCF E (BS-A) = -1495.034162485947

$\omega$1 = -127.76962136

$\omega$2 = 26.30265741

Si -1.98910 -0.36374 2.30329

N 2.07713 0.00445 -0.54224

N 0.66726 2.10718 -2.07735

N 1.89381 2.27830 1.27388

N 2.75206 -3.62813 0.38181

C 2.04591 0.03244 -2.01614

H 1.21063 -0.59718 -2.33489

H 2.95928 -0.40139 -2.45346

C 1.87383 1.43779 -2.58650

H 2.74154 2.04808 -2.32255

H 1.86475 1.37357 -3.68741

C 3.29437 0.61485 0.03403

H 4.13754 0.57537 -0.67074

H 3.59608 0.01917 0.89854

C 3.11260 2.07029 0.48152

H 4.01482 2.37245 1.04294

H 3.04826 2.73087 -0.38594

C 1.84885 -1.35969 -0.02539

H 0.85926 -1.66990 -0.36065

H 1.80197 -1.31320 1.06612

C 2.89176 -2.41417 -0.41856

H 2.82196 -2.63157 -1.50263

H 3.89726 -2.01579 -0.24123

C -0.54124 1.61457 -2.75970

H -0.51312 1.84269 -3.83758

H -1.42260 2.08476 -2.32121

H -0.65171 0.53887 -2.62952

C 0.75677 3.56192 -2.24753

H 1.64914 3.94371 -1.74437

H -0.12111 4.02770 -1.79587

H 0.80871 3.85327 -3.30911

C 1.68021 3.70969 1.52105

H 2.49965 4.15285 2.10993

H 0.74042 3.84589 2.05819

H 1.60435 4.24104 0.57021

C 1.94302 1.55882 2.55150

H 2.00708 0.48343 2.38554

H 1.02676 1.75527 3.11060

H 2.80343 1.86960 3.16710

C 1.47962 -4.30459 0.14564

H 0.64075 -3.67265 0.44568

H 1.43813 -5.21564 0.74874

H 1.33489 -4.58875 -0.91446

C 3.86745 -4.53490 0.14157

H 3.91932 -4.89282 -0.90455

H 3.77754 -5.40956 0.79252

H 4.81121 -4.03192 0.37419

C -1.55656 0.01089 0.52531

H -0.49772 -0.25950 0.39020

C -2.36536 -0.56777 -0.55248

C -3.74427 -0.26656 -0.67574

H -4.19994 0.38780 0.05749

C -4.51288 -0.77149 -1.72134

H -5.56815 -0.51482 -1.77625

C -3.94764 -1.59463 -2.69884

H -4.55160 -1.98863 -3.51050

C -2.58829 -1.90432 -2.60469

H -2.12410 -2.54996 -3.34661

C -1.81986 -1.40187 -1.55768

H -0.77042 -1.67805 -1.49895

C -2.06660 1.19399 3.38733

H -1.14840 1.78666 3.31739

H -2.21845 0.93037 4.44013

H -2.89091 1.84355 3.07709

C -0.64381 -1.48705 3.04153

H -0.58431 -2.43712 2.49897

H -0.83971 -1.71467 4.09583

H 0.34367 -1.01595 2.98722

C -3.64771 -1.25712 2.49089

H -4.49016 -0.61502 2.21778

H -3.79212 -1.57435 3.53010

H -3.69315 -2.14476 1.85222

Li 0.42789 1.51873 -0.04078

C -2.05041 2.03319 0.37488

O -1.06357 2.71568 0.35300

**2**Na + CO → **5**

**2**Na

SCF E (BS-A) = -1536.496946409388

$\omega$1 = 23.08573575

$\omega$2 = 43.73562253

Si -4.33573 -0.41499 -0.10256

Na 0.93013 0.16366 -0.19370

N 1.11606 -0.40987 2.21788

N 3.07008 1.23334 -1.01093

N 0.26616 -1.77680 -1.54973

N 2.80475 -1.58309 0.02640

C 3.13909 -1.68883 1.45246

H 3.81602 -0.87150 1.70776

H 3.69191 -2.61998 1.66847

C 1.91556 -1.63162 2.36581

H 1.25612 -2.47858 2.15303

H 2.25803 -1.76077 3.40902

C 3.94789 -1.11288 -0.76484

H 3.75882 -1.35061 -1.81323

H 4.87431 -1.64980 -0.49380

C 4.20668 0.38583 -0.62689

H 4.45092 0.62375 0.41290

H 5.10598 0.63493 -1.21969

C 2.27120 -2.84712 -0.50021

H 1.66617 -3.31430 0.27876

H 3.08204 -3.56118 -0.72770

C 1.41563 -2.66164 -1.75248

H 2.02361 -2.23508 -2.55727

H 1.09533 -3.66033 -2.10273

C -0.16876 -0.56935 2.90772

H -0.76709 0.32936 2.76165

H -0.03970 -0.74988 3.98891

H -0.71685 -1.41056 2.47663

C 1.81619 0.76999 2.73682

H 2.75893 0.91925 2.20583

H 2.03700 0.68301 3.81507

H 1.19699 1.65358 2.56925

C 3.32856 2.61365 -0.59129

H 2.47675 3.24030 -0.85203

H 4.23605 3.02853 -1.06300

H 3.45321 2.65052 0.49408

C 2.82455 1.19172 -2.45691

H 2.58731 0.17476 -2.77650

H 3.69412 1.54342 -3.03872

H 1.96462 1.82242 -2.69245

C -0.33543 -1.41426 -2.83697

H -1.20049 -0.77680 -2.65024

H -0.66706 -2.30088 -3.40514

H 0.39169 -0.86731 -3.44482

C -0.75596 -2.38112 -0.68517

H -1.12074 -3.34312 -1.08702

H -1.59381 -1.68116 -0.60607

H -0.35592 -2.55979 0.31597

C -2.04207 1.31688 -0.56024

C -1.92630 1.70618 0.82638

H -2.60760 1.26161 1.54562

C -1.02581 2.66301 1.26768

H -1.02047 2.92712 2.32435

C -0.13980 3.31288 0.38929

H 0.54349 4.08008 0.73692

C -0.21204 2.95360 -0.96887

H 0.42725 3.45652 -1.69305

C -1.09925 1.98641 -1.42931

H -1.13640 1.76302 -2.49388

C -2.98157 0.38178 -1.03001

H -3.01069 0.25426 -2.11445

C -5.61652 0.80048 0.61729

H -5.13125 1.54458 1.25884

H -6.38385 0.29211 1.21412

H -6.12050 1.34728 -0.18741

C -3.77139 -1.47698 1.38404

H -3.15488 -2.32073 1.05455

H -4.63178 -1.88537 1.92781

H -3.17751 -0.89477 2.09640

C -5.26118 -1.58705 -1.27770

H -5.66176 -1.04456 -2.14131

H -6.10109 -2.08021 -0.77554

H -4.59432 -2.36877 -1.65903

**A**-Na

SCF E (BS-A) = -1649.811427240421

$\omega$1 = 19.45447275

$\omega$2 = 31.70318452

Si 3.96506 0.89291 -0.48555

Na -0.81431 0.05597 -0.03328

N -1.81808 -1.46868 1.67509

N -2.37914 -0.31001 -2.20455

N -0.64539 2.61091 0.29676

N -3.20178 0.94036 0.42053

C -3.83115 0.00532 1.36896

H -4.34761 -0.76805 0.79800

H -4.61077 0.51345 1.96177

C -2.85072 -0.65982 2.33246

H -2.33686 0.10382 2.92685

H -3.44071 -1.26830 3.04386

C -3.99003 1.03877 -0.81613

H -3.72909 1.96765 -1.32425

H -5.06974 1.10954 -0.59752

C -3.77091 -0.13086 -1.77062

H -4.08461 -1.06213 -1.29030

H -4.44091 0.00748 -2.63962

C -3.00767 2.26263 1.03462

H -2.64500 2.11192 2.05448

H -3.96650 2.80439 1.12252

C -2.01409 3.13925 0.27372

H -2.32434 3.22921 -0.77051

H -2.05463 4.16058 0.69213

C -0.86911 -1.97387 2.67389

H -0.04707 -2.48261 2.16673

H -1.35212 -2.67577 3.37650

H -0.45803 -1.14093 3.24672

C -2.38160 -2.60781 0.94406

H -2.99962 -2.26897 0.11183

H -2.99779 -3.25328 1.59535

H -1.56095 -3.19778 0.53222

C -2.26704 -1.58736 -2.91586

H -1.24972 -1.71875 -3.28532

H -2.95618 -1.64257 -3.77639

H -2.49188 -2.41085 -2.23473

C -1.94485 0.77672 -3.08599

H -2.00754 1.73591 -2.57037

H -2.55294 0.84045 -4.00552

H -0.90168 0.61757 -3.37148

C 0.15743 3.16522 -0.79943

H 1.13817 2.68864 -0.78881

H 0.28490 4.25846 -0.71862

H -0.31924 2.94454 -1.75762

C 0.00329 2.92511 1.57412

H -0.00358 4.01036 1.77657

H 1.03759 2.58729 1.54586

H -0.49990 2.41626 2.39969

C 1.95540 -1.27578 -0.63669

C 2.47310 -2.15773 0.37365

H 3.27169 -1.80679 1.01423

C 2.00349 -3.45293 0.54450

H 2.44942 -4.07028 1.32223

C 0.97798 -3.98081 -0.25317

H 0.62330 -4.99721 -0.11696

C 0.44729 -3.15297 -1.25121

H -0.33110 -3.53444 -1.90813

C 0.91200 -1.85625 -1.43653

H 0.51141 -1.25461 -2.25037

C 2.35560 0.07740 -0.81643

H 1.80437 0.57646 -1.62450

C 4.82636 1.33297 -2.12900

H 5.07675 0.42710 -2.69201

H 5.75155 1.90067 -1.96973

H 4.17104 1.94119 -2.76446

C 5.19449 -0.14473 0.52231

H 4.85667 -0.28660 1.55390

H 6.16406 0.36585 0.55993

H 5.35246 -1.13302 0.07925

C 3.82254 2.55711 0.43742

H 3.17809 3.26755 -0.09199

H 4.80886 3.02667 0.53750

H 3.41724 2.42181 1.44491

C 1.42357 0.12244 1.89435

O 2.30744 0.29372 2.60717

TS(**A**-**B**)-Na

SCF E (BS-A) = -1649.811652125217

$\omega$1 = -85.74342308

$\omega$2 = 26.97013308

Si -4.02817 1.00443 0.41128

Na 0.85486 0.08449 0.00571

N 1.71022 -1.48510 -1.71584

N 2.34695 -0.50217 2.18636

N 0.91708 2.68852 -0.19569

N 3.30068 0.76729 -0.39025

C 3.82999 -0.17453 -1.39326

H 4.29181 -1.01245 -0.86814

H 4.63737 0.29367 -1.98115

C 2.77718 -0.71261 -2.36123

H 2.30098 0.12111 -2.88938

H 3.30026 -1.31465 -3.12834

C 4.09555 0.71806 0.84505

H 3.94113 1.64527 1.39781

H 5.17617 0.67821 0.62392

C 3.74756 -0.46385 1.74418

H 3.94717 -1.39994 1.21473

H 4.43417 -0.44775 2.61113

C 3.23967 2.13549 -0.92756

H 2.86761 2.07923 -1.95363

H 4.24775 2.58361 -0.98594

C 2.33308 3.06126 -0.11551

H 2.63045 3.04067 0.93613

H 2.49432 4.09933 -0.45694

C 0.67108 -1.82924 -2.69359

H -0.16443 -2.31046 -2.18066

H 1.05435 -2.50914 -3.47485

H 0.29953 -0.92246 -3.17316

C 2.20183 -2.71562 -1.09168

H 2.89231 -2.49065 -0.27764

H 2.71996 -3.37054 -1.81510

H 1.35246 -3.25640 -0.66993

C 2.09497 -1.79078 2.83917

H 1.07008 -1.82577 3.21048

H 2.77566 -1.96261 3.69099

H 2.22593 -2.60065 2.11855

C 2.04736 0.58343 3.12351

H 2.21069 1.55342 2.65202

H 2.66940 0.53555 4.03469

H 0.99664 0.52686 3.42028

C 0.15095 3.22651 0.93291

H -0.87750 2.86814 0.86370

H 0.13995 4.33014 0.95300

H 0.57539 2.87051 1.87483

C 0.33455 3.16828 -1.45278

H 0.45213 4.26045 -1.56328

H -0.72767 2.93065 -1.47983

H 0.80707 2.67993 -2.30799

C -2.12240 -1.25323 0.53798

C -2.72722 -2.11535 -0.43173

H -3.51175 -1.71814 -1.06418

C -2.33041 -3.43412 -0.60052

H -2.83049 -4.04061 -1.35292

C -1.30232 -3.99745 0.17097

H -1.00446 -5.03271 0.03956

C -0.68557 -3.18343 1.12603

H 0.10115 -3.59302 1.75470

C -1.07704 -1.85863 1.30240

H -0.60675 -1.25792 2.07857

C -2.42759 0.13909 0.66058

H -1.80189 0.64902 1.40580

C -5.16359 0.93228 1.94085

H -5.42523 -0.10481 2.17938

H -6.09721 1.48760 1.78493

H -4.66689 1.35496 2.82204

C -5.06530 0.37326 -1.04684

H -4.47960 0.34395 -1.96969

H -5.92255 1.03756 -1.20859

H -5.46051 -0.63059 -0.86014

C -3.69187 2.85100 0.09985

H -3.11361 3.29757 0.91701

H -4.62700 3.41681 0.01617

H -3.12888 2.99652 -0.82818

C -1.22773 0.47070 -1.45067

O -1.97093 0.61703 -2.33057

**B**-Na

SCF E (BS-A) = -1649.821111227362

$\omega$1 = 19.86351647

$\omega$2 = 32.56029330

Si -4.02015 1.48117 0.1667

Na 1.17283 0.25065 0.02313

N 1.54759 -1.76417 -1.36045

N 1.78966 -0.0668 2.37927

N 2.23129 2.25814 -0.88821

N 3.68167 -0.1752 0.0407

C 3.88795 -1.52996 -0.48671

H 3.70461 -2.24168 0.32091

H 4.93585 -1.6871 -0.79875

C 2.97588 -1.86849 -1.66839

H 3.18242 -1.18406 -2.4969

H 3.24054 -2.8797 -2.02988

C 4.1144 -0.05884 1.43821

H 4.2452 1.00063 1.6672

H 5.10136 -0.52869 1.59763

C 3.12438 -0.66913 2.43512

H 3.00905 -1.73775 2.22927

H 3.56232 -0.5944 3.44769

C 4.29577 0.84775 -0.81705

H 4.18551 0.52791 -1.85509

H 5.38259 0.92744 -0.63598

C 3.67345 2.23918 -0.6578

H 3.84867 2.60541 0.35925

H 4.21156 2.93253 -1.33186

C 0.74866 -1.72799 -2.58757

H -0.3027 -1.60273 -2.32595

H 0.85712 -2.64995 -3.18597

H 1.05349 -0.87682 -3.20194

C 1.08163 -2.84783 -0.49205

H 1.63569 -2.84742 0.44996

H 1.20526 -3.84027 -0.96122

H 0.02429 -2.70188 -0.26299

C 0.79582 -0.90531 3.04999

H -0.19153 -0.45198 2.93562

H 1.00309 -1.03193 4.12719

H 0.76537 -1.89239 2.58147

C 1.76439 1.28767 2.93232

H 2.4642 1.93237 2.39708

H 2.02522 1.30799 4.00527

H 0.76342 1.70842 2.80536

C 1.60673 3.47522 -0.37085

H 0.52556 3.39193 -0.49768

H 1.96127 4.38508 -0.88753

H 1.82516 3.57873 0.69609

C 1.86385 2.0722 -2.29413

H 2.24093 2.8897 -2.93507

H 0.77249 2.02966 -2.35925

H 2.27006 1.13131 -2.6742

C -2.53989 -0.9651 0.24663

C -2.66157 -1.4754 -1.06201

H -2.70638 -0.77714 -1.89048

C -2.71894 -2.84735 -1.30342

H -2.81744 -3.20785 -2.32387

C -2.64028 -3.75774 -0.2467

H -2.68497 -4.82607 -0.435

C -2.50116 -3.27274 1.05611

H -2.43768 -3.96618 1.8906

C -2.44704 -1.89926 1.29452

H -2.34754 -1.53491 2.31345

C -2.42082 0.50606 0.48038

H -2.17292 0.65537 1.54306

C -5.3321 0.90193 1.41076

H -5.54046 -0.1663 1.28642

H -6.27621 1.44514 1.28537

H -4.99538 1.05469 2.44256

C -4.71712 1.2517 -1.57367

H -3.97842 1.57093 -2.31335

H -5.6281 1.8466 -1.70772

H -4.97004 0.20486 -1.76849

C -3.66742 3.31353 0.48039

H -3.2827 3.46966 1.49496

H -4.57015 3.92452 0.36866

H -2.9137 3.67225 -0.22527

C -1.12498 1.04032 -0.33933

O -1.39066 1.68435 -1.36645

TS(**B**-**C**)-Na

SCF E (BS-A) = -1649.819585730150

$\omega$1 = -26.39993360

$\omega$2 = 18.08878026

Si 3.74062 -1.89778 0.12413

C 2.52040 -0.52966 0.61583

H 2.40620 -0.62428 1.70448

C 2.98430 0.86081 0.31903

C 3.15202 1.32352 -1.00321

H 2.97233 0.63668 -1.81973

C 3.49986 2.64764 -1.26519

H 3.62050 2.97330 -2.29528

C 3.69171 3.55595 -0.22117

H 3.96894 4.58513 -0.42893

C 3.51935 3.11958 1.09415

H 3.66006 3.81170 1.92036

C 3.16720 1.79577 1.35644

H 3.03588 1.46822 2.38481

C 4.19549 -1.85277 -1.70654

H 3.28413 -1.84669 -2.31068

H 4.79431 -2.72939 -1.97907

H 4.77412 -0.95819 -1.95500

C 2.93505 -3.56698 0.51267

H 2.63881 -3.62798 1.56567

H 3.61877 -4.39837 0.30737

H 2.03514 -3.70835 -0.09428

C 5.31435 -1.71720 1.16265

H 5.77471 -0.73758 0.99577

H 6.05428 -2.48562 0.91042

H 5.09487 -1.80256 2.23273

O 1.02190 -0.82002 -1.24738

C 1.06057 -0.79069 -0.00133

Na -1.29329 -0.24047 0.05488

N -2.21174 -0.68340 2.30278

N -2.31082 -1.59818 -1.68256

N -1.36660 2.15319 -0.52940

N -3.77528 0.39945 -0.00872

C -4.39404 -0.13229 1.21135

H -4.62533 -1.18626 1.04237

H -5.35928 0.36060 1.42491

C -3.50147 -0.00705 2.44934

H -3.29671 1.04976 2.64689

H -4.06562 -0.38165 3.32371

C -4.33705 -0.21278 -1.22026

H -4.12642 0.44634 -2.06465

H -5.43698 -0.28979 -1.16151

C -3.76370 -1.60253 -1.51667

H -3.99899 -2.27857 -0.68803

H -4.28068 -2.00968 -2.40567

C -3.81770 1.86633 -0.05545

H -3.69983 2.24294 0.96277

H -4.80067 2.23162 -0.40265

C -2.73361 2.47989 -0.94528

H -2.85759 2.11994 -1.97118

H -2.89497 3.57358 -0.98013

C -1.24143 -0.22555 3.29764

H -0.27202 -0.68281 3.08398

H -1.53983 -0.47870 4.33032

H -1.12432 0.85921 3.22597

C -2.33175 -2.14003 2.35089

H -3.01964 -2.49455 1.57999

H -2.69658 -2.49990 3.32942

H -1.35226 -2.58422 2.15472

C -1.73310 -2.93714 -1.55879

H -0.64527 -2.84731 -1.59425

H -2.06729 -3.61982 -2.36010

H -2.01275 -3.37266 -0.59513

C -1.87974 -0.98514 -2.94014

H -2.27171 0.03102 -3.02487

H -2.22030 -1.55791 -3.82132

H -0.78941 -0.92829 -2.93569

C -0.40990 2.45234 -1.59980

H 0.59383 2.15597 -1.29196

H -0.39808 3.52536 -1.86010

H -0.67041 1.87927 -2.49323

C -0.98011 2.85468 0.69645

H -1.00071 3.95215 0.57365

H 0.03147 2.55522 0.97916

H -1.65138 2.59103 1.51742

**C**-Na

SCF E (BS-A) = -1649.819829725313

$\omega$1 = 19.71070043

$\omega$2 = 29.59180621

Si 3.70805 -1.93497 0.08313

C 2.57457 -0.53374 0.67952

H 2.56094 -0.63695 1.77304

C 3.02922 0.85001 0.34504

C 3.16059 1.29812 -0.98615

H 2.97248 0.60322 -1.79291

C 3.48909 2.62300 -1.27144

H 3.58321 2.93716 -2.30786

C 3.69555 3.54566 -0.24367

H 3.95682 4.57504 -0.47029

C 3.55939 3.12346 1.08089

H 3.71260 3.82679 1.89531

C 3.22831 1.79953 1.36649

H 3.12459 1.48305 2.40146

C 3.92841 -1.95268 -1.79188

H 2.95245 -1.89754 -2.28245

H 4.43013 -2.87308 -2.11180

H 4.52948 -1.10565 -2.13530

C 2.92930 -3.57616 0.61601

H 2.76229 -3.60217 1.69826

H 3.57002 -4.42557 0.35354

H 1.95928 -3.71714 0.12844

C 5.40071 -1.75203 0.91070

H 5.85198 -0.78786 0.65401

H 6.09081 -2.54335 0.59597

H 5.31374 -1.79763 2.00186

O 0.87781 -0.64517 -1.02194

C 1.06581 -0.78625 0.20545

Na -1.27065 -0.24940 0.04211

N -2.23276 -0.72415 2.27846

N -2.29682 -1.56504 -1.72205

N -1.33139 2.16338 -0.48358

N -3.75780 0.41510 -0.02754

C -4.39635 -0.14068 1.17132

H -4.62806 -1.19015 0.97674

H -5.36326 0.35095 1.38079

C -3.52059 -0.04489 2.42348

H -3.31437 1.00658 2.64586

H -4.09814 -0.43505 3.28228

C -4.31042 -0.16239 -1.25961

H -4.07900 0.51194 -2.08645

H -5.41217 -0.22593 -1.21837

C -3.75196 -1.55358 -1.57835

H -4.00859 -2.24423 -0.76850

H -4.26109 -1.93381 -2.48364

C -3.78837 1.88296 -0.03691

H -3.67590 2.23200 0.99165

H -4.76571 2.26487 -0.38253

C -2.69221 2.51168 -0.90050

H -2.81048 2.18063 -1.93683

H -2.84565 3.60718 -0.90720

C -1.26714 -0.28564 3.28707

H -0.29365 -0.72420 3.05487

H -1.56471 -0.57025 4.31193

H -1.16200 0.80196 3.24608

C -2.36328 -2.18029 2.30854

H -3.03426 -2.52285 1.51724

H -2.75579 -2.54629 3.27428

H -1.38246 -2.63020 2.13509

C -1.73517 -2.91135 -1.61356

H -0.64542 -2.83381 -1.61995

H -2.05467 -3.57462 -2.43690

H -2.04117 -3.36445 -0.66625

C -1.83965 -0.93112 -2.95903

H -2.22218 0.08952 -3.02992

H -2.16824 -1.48375 -3.85740

H -0.74951 -0.87943 -2.93825

C -0.36459 2.48028 -1.53911

H 0.63121 2.15755 -1.23366

H -0.33585 3.56046 -1.76715

H -0.62823 1.93817 -2.45087

C -0.94393 2.83057 0.76085

H -0.96775 3.93125 0.66928

H 0.07067 2.52716 1.03053

H -1.61173 2.54165 1.57618

TS(**C**-**5**)-Na

SCF E (BS-A) = -1649.803014143818

$\omega$1 = -250.32018777

$\omega$2 = 17.21987238

Si 3.82698 1.63436 0.08038

C 2.56344 0.22508 -0.67002

H 2.89940 0.40003 -1.69855

C 2.47503 -1.20635 -0.29649

C 2.31528 -1.58861 1.04962

H 2.23104 -0.81455 1.80326

C 2.27639 -2.93387 1.41326

H 2.17247 -3.20447 2.46066

C 2.37206 -3.93515 0.44264

H 2.34018 -4.98208 0.72874

C 2.51601 -3.57293 -0.89798

H 2.60014 -4.34014 -1.66291

C 2.56777 -2.22547 -1.25849

H 2.69587 -1.95312 -2.30328

C 3.59745 2.35194 1.82458

H 2.99064 3.26093 1.79765

H 4.56474 2.57657 2.28966

H 3.07358 1.62978 2.45925

C 4.32388 2.92857 -1.20924

H 4.27367 2.50735 -2.21991

H 5.35376 3.27066 -1.04841

H 3.65372 3.79102 -1.17574

C 5.38803 0.52986 0.28469

H 5.20393 -0.28039 0.99938

H 6.24475 1.11052 0.64939

H 5.67658 0.06362 -0.66470

O 0.84146 1.09656 0.71590

C 1.73344 1.32752 -0.16871

Na -1.09525 0.21883 0.18580

N -1.25370 0.34543 -2.26366

N -2.23220 2.09355 1.28273

N -1.81296 -1.97284 1.01534

N -3.57716 0.02537 -0.38149

C -3.71707 0.43172 -1.78520

H -3.73368 1.52264 -1.82294

H -4.68052 0.09705 -2.20953

C -2.59030 -0.08288 -2.68429

H -2.59568 -1.17757 -2.68962

H -2.80773 0.23124 -3.72209

C -4.30487 0.92152 0.52695

H -4.42894 0.40696 1.48225

H -5.32505 1.12981 0.15820

C -3.59027 2.25527 0.76681

H -3.51909 2.80739 -0.17532

H -4.22040 2.86899 1.43748

C -3.94315 -1.38079 -0.17423

H -3.65181 -1.94517 -1.06216

H -5.03648 -1.50557 -0.07996

C -3.27864 -2.00326 1.05564

H -3.59224 -1.46135 1.95340

H -3.65666 -3.03616 1.16891

C -0.21971 -0.44199 -2.93869

H 0.76035 -0.10988 -2.59520

H -0.26455 -0.34008 -4.03748

H -0.32940 -1.49893 -2.68190

C -1.02319 1.77450 -2.49736

H -1.76920 2.37154 -1.96852

H -1.07142 2.03290 -3.56996

H -0.03900 2.04590 -2.10467

C -1.41817 3.29949 1.10741

H -0.38214 3.04879 1.34381

H -1.76728 4.13841 1.73483

H -1.45221 3.61332 0.06088

C -2.20642 1.67155 2.68194

H -2.74701 0.73044 2.81240

H -2.65745 2.42166 3.35630

H -1.16858 1.51069 2.98502

C -1.25273 -2.24856 2.34044

H -0.16514 -2.18368 2.28984

H -1.52720 -3.25099 2.71321

H -1.61259 -1.50309 3.05513

C -1.26409 -2.91123 0.03152

H -1.53045 -3.95783 0.26235

H -0.17641 -2.82570 0.01314

H -1.63906 -2.67746 -0.96723

**5**

SCF E (BS-A) = -1649.874661833105

$\omega$1 = 20.12005288

$\omega$2 = 25.76400128

Si -3.21401 -2.27658 0.10585

C -2.90370 0.52905 -0.15703

H -3.79907 0.38945 -0.75785

C -2.52887 1.91069 0.07398

C -1.66213 2.31714 1.11829

H -1.23783 1.54759 1.75203

C -1.39596 3.66613 1.34791

H -0.75218 3.94955 2.17754

C -1.94451 4.66075 0.53388

H -1.72437 5.70852 0.71436

C -2.79478 4.27912 -0.50917

H -3.24042 5.03565 -1.15033

C -3.08633 2.93708 -0.72646

H -3.76347 2.65808 -1.53062

C -3.26839 -3.13941 1.78972

H -2.26174 -3.36280 2.15452

H -3.82591 -4.08132 1.73575

H -3.75530 -2.50076 2.53421

C -2.36274 -3.44238 -1.12836

H -2.47064 -3.08049 -2.15632

H -2.81035 -4.44176 -1.07990

H -1.29532 -3.54306 -0.91837

C -4.99366 -2.03725 -0.50241

H -5.55845 -1.38503 0.17101

H -5.51691 -2.99879 -0.55996

H -5.02030 -1.58650 -1.50031

O -1.17674 -0.66586 1.00499

C -2.29542 -0.61418 0.33718

Na 0.84943 -0.12221 0.26215

N 0.67210 0.07297 -2.18281

N 1.81076 -2.24218 1.16194

N 2.25469 1.82120 1.00054

N 3.26029 -0.43651 -0.71605

C 3.05379 -0.72268 -2.14070

H 2.74881 -1.76761 -2.23362

H 3.99380 -0.62621 -2.71383

C 1.99712 0.16803 -2.79867

H 2.31088 1.21479 -2.74330

H 1.95953 -0.08481 -3.87479

C 3.90293 -1.55905 -0.02038

H 4.35822 -1.17596 0.89543

H 4.73124 -1.98163 -0.61607

C 2.93467 -2.68654 0.33699

H 2.51691 -3.11445 -0.57986

H 3.50971 -3.49616 0.82463

C 3.99278 0.81601 -0.49667

H 3.74113 1.51286 -1.29742

H 5.08421 0.66307 -0.56078

C 3.66887 1.46016 0.85151

H 3.92028 0.76409 1.65771

H 4.32263 2.34164 0.98629

C -0.19031 1.17797 -2.61383

H -1.15709 1.09022 -2.11528

H -0.34758 1.18303 -3.70700

H 0.25725 2.13193 -2.32411

C 0.01901 -1.20501 -2.47143

H 0.59279 -2.03638 -2.05442

H -0.10116 -1.37765 -3.55601

H -0.96478 -1.20924 -2.00213

C 0.74266 -3.24275 1.20014

H -0.14648 -2.78696 1.63828

H 1.03185 -4.14708 1.76415

H 0.49259 -3.54216 0.18013

C 2.21447 -1.90177 2.52549

H 2.95076 -1.09529 2.52347

H 2.65081 -2.76375 3.06153

H 1.33886 -1.55753 3.08195

C 1.94406 2.11269 2.40267

H 0.88071 2.33611 2.49917

H 2.51814 2.97121 2.79300

H 2.16760 1.23837 3.02008

C 1.90333 2.96986 0.15923

H 2.48654 3.86930 0.42547

H 0.84170 3.19222 0.27115

H 2.08938 2.74233 -0.89161

**2**Na + CO → **4**

TS(**C**-**D**)-Na

SCF E (BS-A) = -1763.142319615574

$\omega$1 = -57.05838701

$\omega$2 = 22.40984233

O -0.50611 2.40946 2.88410

C -1.03493 2.58609 1.86232

Si -3.84161 1.48639 -0.68301

C -2.67259 0.53368 0.48789

H -2.97006 0.83285 1.50257

C -2.82863 -0.95723 0.38599

C -2.37924 -1.67061 -0.74124

H -1.93985 -1.11734 -1.56183

C -2.47666 -3.05961 -0.80062

H -2.12541 -3.58424 -1.68551

C -3.02276 -3.77990 0.26432

H -3.09934 -4.86186 0.21561

C -3.47473 -3.08813 1.38971

H -3.90257 -3.63179 2.22748

C -3.37585 -1.69811 1.44797

H -3.72115 -1.17014 2.33280

C -3.77254 0.84927 -2.45930

H -2.75351 0.94274 -2.84574

H -4.44268 1.43172 -3.10196

H -4.06902 -0.20148 -2.52563

C -3.37966 3.31620 -0.66572

H -3.26766 3.68178 0.35885

H -4.13540 3.92339 -1.17623

H -2.41929 3.46507 -1.16880

C -5.60138 1.27096 -0.01572

H -5.86842 0.21004 0.03852

H -6.33857 1.77168 -0.65350

H -5.69447 1.68906 0.99279

O -0.74388 0.97141 -0.86100

C -1.16078 0.92248 0.31904

Na 1.28292 0.23341 -0.11556

N 2.64856 2.28777 -0.09194

N 1.74981 -1.13840 -2.12306

N 1.42373 -1.32257 1.78162

N 3.73244 -0.51689 0.03719

C 4.56779 0.67509 -0.15845

H 4.70724 0.82140 -1.23168

H 5.57894 0.53184 0.26143

C 3.96808 1.94481 0.44752

H 3.85402 1.81706 1.52881

H 4.69007 2.77055 0.30572

C 3.95913 -1.51587 -1.01521

H 3.64075 -2.49011 -0.64102

H 5.03256 -1.61804 -1.25113

C 3.20180 -1.20303 -2.30821

H 3.52894 -0.23365 -2.69712

H 3.48329 -1.95416 -3.06950

C 3.88976 -1.07833 1.38513

H 3.97386 -0.24637 2.08795

H 4.82935 -1.65240 1.47694

C 2.73019 -1.98140 1.81474

H 2.67597 -2.85104 1.15251

H 2.96123 -2.37639 2.82177

C 2.00515 3.31602 0.72942

H 0.99854 3.50460 0.35046

H 2.56893 4.26543 0.72865

H 1.91315 2.96394 1.75853

C 2.71417 2.73687 -1.48367

H 3.13402 1.95716 -2.12210

H 3.32896 3.64686 -1.60117

H 1.70259 2.94992 -1.83751

C 1.09051 -0.47777 -3.25171

H 0.03114 -0.34688 -3.02179

H 1.19146 -1.04693 -4.19241

H 1.52250 0.51545 -3.39911

C 1.16599 -2.46210 -1.90597

H 1.62330 -2.94600 -1.04130

H 1.29666 -3.12390 -2.78100

H 0.10024 -2.35983 -1.69798

C 0.33664 -2.30119 1.86038

H -0.62148 -1.78421 1.79422

H 0.36213 -2.87918 2.80108

H 0.39897 -2.99945 1.02252

C 1.26905 -0.32770 2.84679

H 1.32741 -0.78138 3.85224

H 0.30675 0.17129 2.72599

H 2.04595 0.43800 2.77532

**D**-Na

SCF E (BS-A) = -1763.175534796216

$\omega$1 = 18.57206230

$\omega$2 = 27.21527431

O -0.65638 -1.85244 -3.06012

C -1.01967 -1.39413 -2.00748

Si -3.70087 -1.61105 0.76841

C -2.88381 -0.61230 -0.65447

H -3.43673 -0.90868 -1.55790

C -3.05442 0.87492 -0.46360

C -2.23235 1.58664 0.42698

H -1.45945 1.02933 0.94424

C -2.39689 2.95833 0.60868

H -1.75052 3.49442 1.29916

C -3.39254 3.65193 -0.08400

H -3.52255 4.71991 0.06342

C -4.22034 2.95542 -0.96501

H -4.99759 3.48112 -1.51266

C -4.05099 1.58253 -1.15081

H -4.69605 1.04898 -1.84430

C -3.08968 -1.05182 2.46137

H -2.00917 -1.20806 2.50129

H -3.57090 -1.61526 3.26862

H -3.28966 0.01229 2.62081

C -3.29651 -3.43330 0.49144

H -3.60887 -3.76004 -0.50609

H -3.79190 -4.07505 1.22821

H -2.21571 -3.58229 0.57021

C -5.56957 -1.32940 0.64955

H -5.80377 -0.26147 0.71384

H -6.10320 -1.83981 1.45901

H -5.96920 -1.70111 -0.30052

O -0.62909 -1.07207 0.28540

C -1.43413 -1.06944 -0.78096

Na 1.35403 -0.16676 0.11731

N 2.67333 -2.20051 -0.10931

N 1.67307 0.98929 2.26617

N 1.64816 1.27553 -1.84580

N 3.79885 0.55939 0.17071

C 4.60467 -0.66372 0.28171

H 4.65898 -0.94068 1.33700

H 5.64638 -0.49261 -0.04153

C 4.03118 -1.83858 -0.51629

H 3.99548 -1.57419 -1.57772

H 4.72931 -2.69216 -0.43144

C 3.95454 1.43283 1.34022

H 3.65881 2.44452 1.05372

H 5.00995 1.50292 1.65635

C 3.11409 0.98296 2.53751

H 3.39324 -0.03865 2.81291

H 3.36566 1.62012 3.40522

C 4.03660 1.27478 -1.09021

H 4.28623 0.54076 -1.85894

H 4.91027 1.94509 -1.01422

C 2.82803 2.09428 -1.55076

H 2.55061 2.80402 -0.76452

H 3.12807 2.70004 -2.42592

C 1.99126 -3.00897 -1.12200

H 0.95708 -3.16373 -0.80845

H 2.47424 -3.98994 -1.27609

H 1.97363 -2.47469 -2.07449

C 2.62986 -2.87781 1.18522

H 3.08478 -2.25801 1.96169

H 3.15733 -3.84843 1.17000

H 1.58590 -3.04968 1.45919

C 0.93756 0.18955 3.24951

H -0.11557 0.14577 2.96395

H 1.01547 0.59930 4.27159

H 1.32438 -0.83274 3.25458

C 1.13169 2.34716 2.21767

H 1.60845 2.92694 1.42430

H 1.27026 2.88717 3.17112

H 0.06409 2.30033 1.99961

C 0.43330 2.09487 -1.91092

H -0.42937 1.44147 -2.05953

H 0.46966 2.83732 -2.72731

H 0.28993 2.62518 -0.96611

C 1.80728 0.52043 -3.09209

H 1.96319 1.18784 -3.95854

H 0.92060 -0.08951 -3.27131

H 2.66547 -0.15242 -3.02592

TS(**D**-**E**)-Na

SCF E (BS-A) = -3299.673302290916

$\omega$1 = -196.18770783

$\omega$2 = 14.99474621

Si 1.64334 -4.13463 -0.04519

C 2.34776 -2.50783 0.70909

H 1.96153 -2.53176 1.73699

C 3.83802 -2.69616 0.76354

C 4.66291 -2.46172 -0.35418

H 4.20257 -1.99415 -1.21635

C 6.00980 -2.82228 -0.33783

H 6.62328 -2.64074 -1.21711

C 6.57370 -3.43647 0.78425

H 7.61899 -3.73112 0.78487

C 5.77260 -3.66717 1.90379

H 6.19401 -4.13550 2.78923

C 4.42905 -3.28887 1.89531

H 3.81335 -3.47702 2.77101

C 2.40216 -4.53545 -1.72903

H 2.23237 -3.73235 -2.44899

H 1.95884 -5.45306 -2.13377

H 3.48117 -4.69207 -1.63932

C -0.23789 -4.06021 -0.16438

H -0.66693 -3.86561 0.82140

H -0.64438 -5.00357 -0.54742

H -0.54982 -3.24512 -0.81902

C 2.06993 -5.57272 1.12111

H 3.15153 -5.70564 1.21720

H 1.64557 -6.51160 0.74635

H 1.66390 -5.40287 2.12494

O 2.46258 -0.60878 -0.94147

C 1.77812 -1.21503 0.02590

Na 3.72314 1.00502 0.05384

N 5.38774 0.97388 1.97235

N 2.01504 2.69546 0.81328

N 5.14937 1.50449 -1.90164

N 4.83401 3.44561 0.36323

C 5.47382 3.46978 1.68040

H 4.70266 3.64216 2.43207

H 6.18351 4.31254 1.77354

C 6.21541 2.18073 2.02171

H 7.04408 2.03320 1.32199

H 6.67615 2.30650 3.02050

C 3.74301 4.42077 0.27652

H 3.45338 4.50912 -0.77295

H 4.07405 5.42864 0.59034

C 2.51558 4.03514 1.10524

H 2.75325 4.07930 2.17340

H 1.73816 4.80462 0.93281

C 5.80265 3.61512 -0.72529

H 6.75728 3.19184 -0.40679

H 5.99895 4.68313 -0.92817

C 5.36959 2.94584 -2.03176

H 4.42920 3.38964 -2.37413

H 6.12613 3.17709 -2.80599

C 6.24243 -0.20900 2.07437

H 5.62914 -1.10579 2.04453

H 6.83180 -0.21638 3.00952

H 6.93311 -0.24316 1.22938

C 4.37501 0.95004 3.03118

H 3.68594 1.78818 2.91907

H 4.82252 0.98957 4.04077

H 3.79075 0.02963 2.94522

C 0.97983 2.30546 1.77647

H 0.64258 1.29120 1.56256

H 0.10439 2.97491 1.74425

H 1.39778 2.32919 2.78873

C 1.48623 2.60853 -0.54995

H 2.28092 2.78373 -1.27975

H 0.68347 3.33998 -0.72963

H 1.09728 1.60353 -0.71380

C 4.45132 0.95628 -3.06723

H 4.09243 -0.04889 -2.83451

H 5.09426 0.92940 -3.96522

H 3.57622 1.56823 -3.28932

C 6.39027 0.76689 -1.67469

H 7.09949 0.87371 -2.51603

H 6.15966 -0.29181 -1.54456

H 6.88442 1.10865 -0.76394

O -0.23478 -1.17850 1.45066

C 0.51847 -1.00177 0.48693

Si -0.48110 -0.17366 -2.83670

Na -4.81853 0.27121 0.41131

N -5.84871 1.37722 -1.49631

N -6.09275 0.89208 2.51796

N -4.44085 -2.13981 0.11960

N -7.11418 -0.74825 0.20598

C -7.86477 0.07977 -0.74855

H -8.27688 0.93562 -0.21098

H -8.73088 -0.46669 -1.16047

C -7.01067 0.58349 -1.91236

H -6.63203 -0.26841 -2.48533

H -7.66230 1.15417 -2.59901

C -7.73424 -0.74581 1.53628

H -7.32275 -1.58083 2.10663

H -8.82236 -0.92524 1.47549

C -7.50701 0.55239 2.30859

H -7.96395 1.38669 1.76838

H -8.04378 0.47810 3.27189

C -6.91647 -2.11648 -0.29477

H -6.77064 -2.06640 -1.37539

H -7.81726 -2.73477 -0.13607

C -5.72019 -2.82365 0.34297

H -5.86986 -2.89620 1.42484

H -5.68976 -3.86207 -0.03397

C -4.91339 1.53480 -2.61468

H -4.02830 2.07428 -2.27298

H -5.36503 2.07971 -3.46191

H -4.59300 0.55252 -2.96574

C -6.22903 2.69241 -0.97267

H -6.89121 2.58479 -0.11063

H -6.74542 3.30842 -1.72955

H -5.32918 3.21733 -0.64197

C -5.99146 2.27516 2.99494

H -4.94454 2.54021 3.14073

H -6.52859 2.42771 3.94676

H -6.40994 2.95345 2.24712

C -5.45528 -0.01639 3.47742

H -5.48575 -1.04436 3.11009

H -5.94304 0.01141 4.46701

H -4.40550 0.26051 3.59968

C -3.41550 -2.67578 1.02641

H -2.46483 -2.15628 0.88951

H -3.24582 -3.75314 0.86514

H -3.73609 -2.52938 2.06257

C -3.99557 -2.27320 -1.27358

H -3.83265 -3.32742 -1.55577

H -3.06310 -1.72355 -1.40830

H -4.73619 -1.84693 -1.95474

C -1.64747 1.01661 -0.42392

C -1.76965 2.31164 -1.02169

H -1.39851 2.45353 -2.03093

C -2.32734 3.39664 -0.35950

H -2.38944 4.35385 -0.87338

C -2.79653 3.28395 0.95952

H -3.20328 4.14108 1.48704

C -2.69948 2.02970 1.57512

H -3.00974 1.91011 2.61079

C -2.19406 0.91891 0.90048

H -2.07984 -0.02147 1.42553

C -0.98945 -0.08841 -1.05866

H -1.31608 -1.05489 -0.69066

C 0.70260 1.18539 -3.42805

H 0.32766 2.20591 -3.30612

H 0.93622 1.03589 -4.48925

H 1.62030 1.07372 -2.85070

C -1.97557 -0.09041 -4.03244

H -2.72134 -0.85937 -3.79845

H -1.65630 -0.24333 -5.07095

H -2.47291 0.88382 -3.98037

C 0.42145 -1.79351 -3.17291

H 0.67202 -1.88771 -4.23661

H -0.15250 -2.67899 -2.88437

H 1.34605 -1.75326 -2.58753

**E**-Na

SCF E (BS-A) = -3299.749433934019

$\omega$1 = 23.51609698

$\omega$2 = 26.72674839

Si 0.12651 -2.40661 -3.11524

C 0.55908 -1.89259 -1.30955

H -0.28564 -2.28254 -0.74551

C 1.76567 -2.71288 -0.95805

C 3.04771 -2.42547 -1.47192

H 3.17692 -1.47651 -1.97788

C 4.08973 -3.34510 -1.36067

H 5.05801 -3.11648 -1.79906

C 3.89931 -4.57251 -0.71772

H 4.71032 -5.29151 -0.64963

C 2.64935 -4.85726 -0.16698

H 2.48152 -5.80111 0.34534

C 1.60676 -3.93602 -0.27819

H 0.63046 -4.18396 0.12898

C 1.22412 -1.50241 -4.35920

H 1.18656 -0.42270 -4.21522

H 0.90756 -1.72672 -5.38432

H 2.26560 -1.82266 -4.25284

C -1.70888 -2.12177 -3.45620

H -2.28307 -2.98190 -3.08856

H -1.91866 -2.01369 -4.52650

H -2.03927 -1.24155 -2.89511

C 0.44799 -4.26618 -3.36272

H 1.50789 -4.51751 -3.26141

H 0.11574 -4.57507 -4.36158

H -0.10262 -4.86674 -2.62974

O 1.82079 0.21283 -1.07318

C 0.60531 -0.35354 -1.15645

Na 3.30498 0.29308 0.56027

N 3.86081 -1.19520 2.50849

N 2.28296 2.27458 1.60544

N 5.42982 0.91591 -0.56082

N 5.01873 1.58735 2.35223

C 5.06829 0.74888 3.55009

H 4.21066 0.99943 4.17774

H 5.96338 0.96309 4.16259

C 5.04496 -0.74712 3.24580

H 5.91983 -1.01330 2.64414

H 5.14859 -1.29515 4.20183

C 4.45646 2.90982 2.63552

H 4.74557 3.58376 1.82676

H 4.87994 3.34660 3.55823

C 2.92911 2.89262 2.75749

H 2.63816 2.32208 3.64592

H 2.58176 3.92943 2.92934

C 6.30958 1.66437 1.66527

H 6.83165 0.71553 1.80667

H 6.96307 2.43774 2.10825

C 6.17600 1.94591 0.16711

H 5.65380 2.89603 0.02102

H 7.19003 2.08066 -0.25407

C 4.05077 -2.57220 2.04986

H 3.17647 -2.89469 1.48673

H 4.20962 -3.27298 2.88951

H 4.91399 -2.62580 1.38423

C 2.64230 -1.10720 3.31351

H 2.44990 -0.07482 3.60876

H 2.69770 -1.72723 4.22675

H 1.79373 -1.44816 2.71583

C 0.88665 1.92314 1.86593

H 0.50086 1.31374 1.04413

H 0.24025 2.81134 1.98513

H 0.82845 1.33520 2.78703

C 2.40602 3.08225 0.39031

H 3.44653 3.37287 0.23547

H 1.80087 4.00545 0.43652

H 2.09476 2.47286 -0.46071

C 5.02755 1.39235 -1.88749

H 4.37438 0.65472 -2.35698

H 5.89351 1.58550 -2.54507

H 4.45198 2.31439 -1.78798

C 6.19663 -0.32315 -0.68522

H 7.14872 -0.17360 -1.22580

H 5.60155 -1.06034 -1.22436

H 6.42229 -0.73909 0.29915

O -1.81508 -0.22903 -1.06579

C -0.59979 0.33521 -1.16092

Si -0.13219 2.35491 -3.15731

Na -3.30628 -0.27938 0.56152

N -5.43696 -0.90646 -0.55138

N -3.84924 1.22171 2.50260

N -2.28822 -2.24887 1.63143

N -5.02463 -1.55574 2.36930

C -6.31451 -1.63880 1.68165

H -6.83665 -0.68887 1.81503

H -6.96835 -2.40855 2.13038

C -6.18000 -1.93244 0.18595

H -5.65555 -2.88249 0.04751

H -7.19369 -2.07312 -0.23409

C -5.07476 -0.70385 3.55760

H -4.22177 -0.95241 4.19230

H -5.97399 -0.90589 4.16815

C -5.04126 0.78859 3.23647

H -5.91050 1.05262 2.62557

H -5.14838 1.34821 4.18536

C -4.46219 -2.87519 2.66582

H -4.74999 -3.55680 1.86298

H -4.88636 -3.30355 3.59216

C -2.93487 -2.85625 2.78874

H -2.64467 -2.27748 3.67208

H -2.58734 -3.89135 2.97048

C -5.03884 -1.39388 -1.87551

H -4.39863 -0.65392 -2.35885

H -5.90758 -1.60396 -2.52414

H -4.45148 -2.30780 -1.76988

C -6.20788 0.32931 -0.68467

H -6.43480 0.75154 0.29669

H -7.15959 0.17273 -1.22389

H -5.61524 1.06435 -1.22929

C -4.02759 2.59508 2.02824

H -3.14753 2.90668 1.46763

H -4.18790 3.30558 2.85935

H -4.88560 2.64695 1.35584

C -2.63732 1.13522 3.31768

H -2.45444 0.10537 3.62719

H -2.69507 1.76713 4.22262

H -1.78185 1.46241 2.72208

C -0.89213 -1.89473 1.88993

H -0.50580 -1.29143 1.06382

H -0.24566 -2.78192 2.01641

H -0.83447 -1.29998 2.80668

C -2.41006 -3.06824 0.42382

H -1.80500 -3.99101 0.47971

H -2.09777 -2.46693 -0.43272

H -3.45057 -3.36023 0.27104

C -1.75820 2.69798 -0.99266

C -3.04418 2.40796 -1.49526

H -3.18097 1.45443 -1.99037

C -4.08304 3.33185 -1.38881

H -5.05434 3.10095 -1.81916

C -3.88586 4.56632 -0.76177

H -4.69464 5.28816 -0.69751

C -2.63184 4.85435 -0.22229

H -2.45822 5.80388 0.27745

C -1.59239 3.92906 -0.32893

H -0.61308 4.17971 0.06893

C -0.55493 1.87113 -1.34099

H 0.29451 2.26938 -0.78995

C -0.47431 4.20577 -3.43879

H -1.53695 4.44586 -3.33806

H -0.14952 4.49981 -4.44453

H 0.07145 4.82636 -2.71905

C -1.21739 1.41525 -4.38523

H -1.16190 0.33896 -4.22417

H -0.90434 1.62874 -5.41373

H -2.26412 1.71993 -4.28373

C 1.70511 2.07922 -3.49525

H 2.27463 2.94872 -3.14269

H 1.91460 1.95397 -4.56374

H 2.04143 1.21033 -2.92036

TS(**E**-**F**)-Na

SCF E (BS-A) = -3299.740305971534

$\omega$1 = -54.80371864

$\omega$2 = 11.69734306

Si -0.86660 1.46822 -2.80221

Na -2.51202 0.65550 0.68086

N -4.17344 2.53116 0.77868

N -2.33940 0.28203 3.14472

N -6.07044 -0.53775 -1.68916

N -4.64041 -0.44728 1.24123

C -5.69943 0.58674 1.23502

H -5.87146 0.91777 2.26271

H -6.64607 0.16011 0.87945

C -5.37325 1.79464 0.36007

H -5.21914 1.44396 -0.65870

H -6.25743 2.45854 0.33237

C -4.43175 -0.99421 2.59418

H -3.84335 -1.90989 2.49827

H -5.38946 -1.28745 3.05956

C -3.71727 -0.03341 3.54510

H -4.27040 0.90675 3.60465

H -3.73967 -0.47359 4.55897

C -4.89772 -1.55361 0.30071

H -5.84796 -2.05863 0.54607

H -4.10510 -2.28513 0.46649

C -4.85970 -1.19273 -1.18604

H -3.99957 -0.54097 -1.37318

H -4.65916 -2.12483 -1.75028

C -3.67790 3.35598 -0.32636

H -2.76340 3.86805 -0.02172

H -4.41851 4.10805 -0.65195

H -3.43171 2.71623 -1.17188

C -4.42777 3.37607 1.94275

H -4.78518 2.78200 2.78609

H -5.18386 4.15346 1.73117

H -3.50094 3.87294 2.24065

C -1.84536 1.44653 3.88220

H -0.82284 1.66979 3.56944

H -1.84134 1.27748 4.97366

H -2.46888 2.31665 3.66833

C -1.45076 -0.85868 3.37909

H -1.80466 -1.73901 2.84796

H -1.37377 -1.10487 4.45316

H -0.45630 -0.63152 2.99597

C -5.82069 0.10200 -2.97646

H -6.73255 0.59876 -3.32122

H -5.50044 -0.61058 -3.75829

H -5.03697 0.85709 -2.87382

C -7.19269 -1.46223 -1.79428

H -6.99588 -2.29158 -2.50023

H -8.07885 -0.92276 -2.14104

H -7.42833 -1.89778 -0.82057

C 0.40730 2.81515 -0.38117

C -0.26574 2.88744 0.85557

H -0.66060 1.96722 1.27331

C -0.36058 4.07293 1.58431

H -0.87500 4.07290 2.54233

C 0.22284 5.24691 1.10510

H 0.15496 6.17205 1.66903

C 0.89905 5.20446 -0.12078

H 1.35636 6.10792 -0.51666

C 0.98112 4.02099 -0.84791

H 1.48015 4.01849 -1.81229

C 0.49201 1.57755 -1.18143

H 1.51383 1.39986 -1.52746

C 0.64179 1.48648 -4.04079

H 1.22481 2.40486 -3.89167

H 0.33995 1.43887 -5.09555

H 1.31610 0.64335 -3.85104

C -1.64779 3.20501 -2.99077

H -2.73362 3.15112 -3.12253

H -1.24212 3.66581 -3.89929

H -1.43289 3.87900 -2.15813

C -2.00830 0.36822 -3.92138

H -1.62333 -0.65832 -3.97678

H -2.06803 0.74729 -4.95034

H -3.02471 0.29977 -3.52197

C -0.23566 0.36426 -0.74499

O -1.53922 0.51688 -1.33102

Si 0.05939 -3.18168 -2.04775

Na 3.48670 -0.04661 -0.05151

N 4.68039 -2.27517 -0.19234

N 3.26988 1.11007 2.12984

N 4.82167 1.45708 -1.54421

N 5.89961 0.16210 0.98699

C 6.38376 -1.19196 1.27699

H 5.89851 -1.53158 2.19509

H 7.46961 -1.19922 1.48409

C 6.09704 -2.19370 0.15726

H 6.64589 -1.90607 -0.74504

H 6.50209 -3.17705 0.46233

C 5.75946 0.95472 2.21617

H 5.83959 2.01239 1.95792

H 6.58520 0.75111 2.91976

C 4.42915 0.71227 2.93065

H 4.32743 -0.35502 3.15461

H 4.45051 1.23768 3.90427

C 6.72697 0.84084 -0.01591

H 7.09244 0.09621 -0.72488

H 7.62662 1.29128 0.43986

C 5.97965 1.93392 -0.77835

H 5.61271 2.68586 -0.07322

H 6.70276 2.45059 -1.43707

C 4.48636 -2.95461 -1.47249

H 3.42744 -2.93564 -1.73009

H 4.82276 -4.00674 -1.44880

H 5.04105 -2.43499 -2.25862

C 3.88755 -2.94200 0.84311

H 3.99748 -2.42594 1.80057

H 4.18803 -3.99599 0.98346

H 2.83791 -2.88073 0.55743

C 2.02938 0.54864 2.66872

H 1.21105 0.76078 1.97956

H 1.78604 0.95672 3.66707

H 2.11872 -0.53726 2.74368

C 3.15165 2.56541 2.01814

H 4.03435 2.98904 1.53475

H 3.03751 3.04724 3.00615

H 2.28272 2.81418 1.41045

C 4.03480 2.60310 -2.00850

H 3.18735 2.25133 -2.59896

H 4.63086 3.29162 -2.63340

H 3.64435 3.15612 -1.15161

C 5.21950 0.63264 -2.68734

H 5.85613 1.18534 -3.40080

H 4.32448 0.29194 -3.21424

H 5.76561 -0.25004 -2.35139

C -0.78236 -2.97105 0.61576

C 0.18954 -3.06621 1.62577

H 1.06947 -2.44366 1.52307

C 0.00414 -3.90197 2.72549

H 0.76831 -3.94884 3.49744

C -1.15728 -4.66863 2.85149

H -1.30118 -5.31639 3.71104

C -2.12845 -4.59511 1.85148

H -3.03520 -5.18939 1.92597

C -1.93581 -3.76248 0.74706

H -2.68488 -3.73370 -0.03919

C -0.59638 -2.09739 -0.59925

H -1.58040 -1.76231 -0.94342

C -1.45247 -3.83550 -2.98603

H -2.08063 -4.45989 -2.34107

H -1.15884 -4.44205 -3.85019

H -2.06494 -3.00390 -3.35123

C 1.02650 -4.67643 -1.40672

H 1.86189 -4.37955 -0.76888

H 1.42622 -5.26655 -2.23941

H 0.37618 -5.32513 -0.81167

C 1.12337 -2.16366 -3.22603

H 0.50981 -1.48259 -3.82000

H 1.68315 -2.80747 -3.91397

H 1.82594 -1.56301 -2.64271

C 0.29081 -0.86535 -0.43559

O 1.54672 -1.06374 -0.09839

**F**-Na

SCF E (BS-A) = -3299.756139819432

$\omega$1 = 21.00672688

$\omega$2 = 26.96021290

Si -1.54735 0.70469 -2.90670

Na -2.68998 0.88234 0.36062

N -4.41985 2.60020 0.07657

N -2.19208 1.01620 2.80232

N -6.86226 -0.86870 -0.85865

N -4.71852 -0.09468 1.53777

C -5.74309 0.97377 1.49207

H -5.63766 1.58715 2.38932

H -6.75076 0.53763 1.53109

C -5.68794 1.87936 0.26205

H -5.87415 1.27819 -0.62706

H -6.53059 2.59241 0.34185

C -4.29791 -0.35318 2.93129

H -3.75668 -1.30169 2.94135

H -5.17516 -0.49277 3.58725

C -3.41884 0.72905 3.55302

H -3.97718 1.66533 3.64904

H -3.18379 0.40632 4.58475

C -5.19379 -1.37790 0.98117

H -6.04125 -1.76078 1.57439

H -4.37509 -2.08693 1.11523

C -5.54018 -1.39124 -0.50537

H -4.78760 -0.80367 -1.03844

H -5.42982 -2.43499 -0.86195

C -4.44029 3.31199 -1.20529

H -3.48349 3.81478 -1.35778

H -5.24495 4.06658 -1.24337

H -4.59217 2.60768 -2.02525

C -4.18085 3.57741 1.14368

H -4.09795 3.08103 2.10934

H -4.99118 4.32508 1.20422

H -3.23955 4.09390 0.94784

C -1.50248 2.16225 3.40044

H -0.60265 2.39236 2.82959

H -1.21405 1.96535 4.44787

H -2.15278 3.04052 3.38195

C -1.29613 -0.14546 2.77683

H -1.80396 -1.01395 2.36211

H -0.93228 -0.41319 3.78322

H -0.44199 0.06031 2.13071

C -6.97313 -0.71413 -2.30639

H -7.95017 -0.29136 -2.55687

H -6.86524 -1.67269 -2.84784

H -6.19990 -0.03465 -2.67275

C -7.94571 -1.71146 -0.36440

H -7.89893 -2.74241 -0.76368

H -8.90453 -1.27585 -0.65887

H -7.92889 -1.76930 0.72571

C 0.70729 2.65850 -0.90385

C -0.20852 2.97632 0.16075

H -0.66099 2.15115 0.70040

C -0.44264 4.28079 0.57911

H -1.10552 4.44823 1.42655

C 0.17435 5.37626 -0.03897

H -0.01705 6.39263 0.28924

C 1.05088 5.10313 -1.10639

H 1.53747 5.92881 -1.62401

C 1.30540 3.81014 -1.52808

H 1.96328 3.64736 -2.37790

C 0.99007 1.34187 -1.30483

H 1.75562 1.20499 -2.07240

C -0.47498 -0.30213 -4.07604

H 0.58503 -0.13425 -3.87584

H -0.68234 -0.03071 -5.11748

H -0.67883 -1.37036 -3.95761

C -1.46371 2.54596 -3.28766

H -2.24163 2.84000 -4.00242

H -0.48793 2.82247 -3.69366

H -1.58660 3.12711 -2.37047

C -3.34064 0.14051 -3.14605

H -3.43613 -0.93908 -2.98973

H -3.69334 0.36544 -4.15934

H -4.02304 0.64106 -2.45216

C 0.19474 0.18262 -0.94961

O -1.25014 0.28838 -1.29609

Si 0.56259 -3.67999 -1.72772

Na 3.65662 0.02478 -0.07925

N 4.91676 -2.15401 0.41345

N 3.35907 1.53330 1.85642

N 5.22704 1.20234 -1.64830

N 6.08782 0.45237 1.17564

C 6.55236 -0.81927 1.73378

H 6.01855 -0.98997 2.67159

H 7.62598 -0.78390 1.99631

C 6.31591 -2.00691 0.79998

H 6.90216 -1.87743 -0.11560

H 6.70800 -2.91887 1.29011

C 5.81791 1.44002 2.22612

H 5.91168 2.43934 1.79616

H 6.56901 1.38523 3.03371

C 4.42125 1.28487 2.82976

H 4.30167 0.26259 3.20430

H 4.33438 1.95400 3.70708

C 6.98587 0.96633 0.14013

H 7.43602 0.11874 -0.38011

H 7.82835 1.53292 0.57654

C 6.28423 1.86571 -0.87623

H 5.82194 2.71018 -0.35640

H 7.05278 2.29355 -1.54785

C 4.76040 -3.04006 -0.73788

H 3.71135 -3.03998 -1.03113

H 5.07524 -4.07782 -0.52262

H 5.35660 -2.66898 -1.57641

C 4.07688 -2.62729 1.51388

H 4.13491 -1.94233 2.36374

H 4.37369 -3.63383 1.86282

H 3.04575 -2.63783 1.16029

C 2.07154 1.00041 2.30199

H 1.34632 1.13215 1.49762

H 1.70209 1.49907 3.21651

H 2.16014 -0.07160 2.49788

C 3.22452 2.95718 1.54009

H 4.14914 3.34497 1.10629

H 2.99121 3.55616 2.43923

H 2.42614 3.09428 0.81230

C 4.46267 2.20628 -2.39228

H 3.67575 1.71539 -2.96833

H 5.09810 2.78206 -3.08904

H 3.98277 2.89808 -1.69755

C 5.76373 0.20033 -2.56928

H 6.45662 0.63864 -3.31020

H 4.93809 -0.27445 -3.10613

H 6.29511 -0.57920 -2.02150

C -0.76550 -2.83096 0.58309

C 0.07107 -2.85971 1.71268

H 1.05695 -2.42224 1.61323

C -0.36882 -3.39813 2.91891

H 0.29538 -3.40136 3.77925

C -1.65892 -3.92572 3.03458

H -2.00014 -4.34754 3.97514

C -2.50074 -3.90768 1.92193

H -3.50498 -4.31746 1.99243

C -2.05621 -3.36639 0.71184

H -2.71765 -3.35833 -0.15136

C -0.28853 -2.27506 -0.73456

H -1.17375 -1.99222 -1.31106

C -0.82390 -4.63628 -2.60515

H -1.54287 -5.03667 -1.88174

H -0.42865 -5.47972 -3.18261

H -1.37277 -3.98716 -3.29723

C 1.44418 -4.90896 -0.59301

H 2.19411 -4.41784 0.02953

H 1.94346 -5.68710 -1.18184

H 0.72709 -5.39639 0.07476

C 1.75438 -3.00637 -3.03302

H 1.22541 -2.64043 -3.91671

H 2.45674 -3.78330 -3.35595

H 2.31521 -2.17856 -2.59324

C 0.64605 -1.07001 -0.62060

O 1.88691 -1.33461 -0.26412

TS(**F**-**G**)-Na

SCF E (BS-A) = -3299.751797988180

$\omega$1 = -223.72361560

$\omega$2 = 17.36768883

Si 1.77984 -0.27988 3.07801

Na 2.31670 0.92083 -0.49478

N 3.69299 2.91963 -0.00485

N 2.16878 1.32147 -3.00933

N 6.29911 -0.56605 1.25773

N 4.57510 0.28675 -1.38708

C 5.45075 1.41902 -0.99651

H 5.54621 2.09871 -1.84667

H 6.46000 1.05679 -0.77127

C 4.95237 2.19897 0.22113

H 4.80269 1.49984 1.04530

H 5.75343 2.89239 0.54081

C 4.41809 0.20133 -2.85081

H 3.97190 -0.76977 -3.07915

H 5.40092 0.21603 -3.35523

C 3.56208 1.30167 -3.46833

H 3.99548 2.28030 -3.24808

H 3.60856 1.18104 -4.56741

C 5.07842 -1.01676 -0.91561

H 6.05178 -1.24570 -1.38217

H 4.37499 -1.76635 -1.28304

C 5.15879 -1.20423 0.59698

H 4.23737 -0.81425 1.04044

H 5.16169 -2.29637 0.79067

C 3.10500 3.34665 1.27097

H 2.19624 3.91768 1.07828

H 3.80401 3.96787 1.85929

H 2.83140 2.47124 1.85592

C 3.87329 4.08739 -0.86440

H 4.26893 3.80118 -1.84052

H 4.56762 4.82346 -0.41963

H 2.90598 4.57079 -1.01378

C 1.51514 2.53305 -3.50590

H 0.47837 2.55978 -3.16643

H 1.51857 2.58111 -4.60964

H 2.02813 3.41543 -3.11866

C 1.43459 0.14199 -3.47210

H 1.87054 -0.76937 -3.06816

H 1.42332 0.06471 -4.57406

H 0.40512 0.19370 -3.11625

C 6.14657 -0.63819 2.70667

H 6.96518 -0.09847 3.19176

H 6.14901 -1.67774 3.08560

H 5.20442 -0.17754 3.00610

C 7.57479 -1.15394 0.86553

H 7.63279 -2.23555 1.09338

H 8.38380 -0.65028 1.40209

H 7.75496 -1.02328 -0.20356

C -0.53633 2.55329 0.95918

C 0.00660 2.91945 -0.31047

H 0.26018 2.12592 -1.00664

C 0.15935 4.24507 -0.70230

H 0.55249 4.46184 -1.69178

C -0.19830 5.30221 0.14210

H -0.07468 6.33488 -0.16753

C -0.71851 4.97833 1.40526

H -0.99314 5.77446 2.09465

C -0.87726 3.65879 1.80223

H -1.25837 3.44431 2.79757

C -0.74898 1.20760 1.38760

H -1.16883 1.12961 2.39366

C 0.17490 -0.35530 4.07884

H -0.18544 0.64534 4.33108

H 0.35205 -0.89989 5.01429

H -0.61329 -0.87036 3.52644

C 2.82198 1.04799 3.94534

H 3.78779 1.24914 3.47390

H 3.01745 0.73668 4.97885

H 2.27118 1.99392 3.98396

C 2.62671 -1.95458 3.36942

H 1.93326 -2.77079 3.15163

H 2.92622 -2.05228 4.41971

H 3.51660 -2.10770 2.75434

C -0.07250 0.07386 0.88731

O 1.59513 0.00842 1.45428

Si -0.18228 -3.92583 1.04845

Na -3.45840 0.05390 0.19765

N -4.80135 -2.08774 -0.19619

N -3.45690 1.51987 -1.80305

N -4.82686 1.27511 1.93864

N -6.08699 0.52277 -0.72047

C -6.63414 -0.74378 -1.21038

H -6.26110 -0.90496 -2.22425

H -7.73606 -0.70892 -1.29393

C -6.24595 -1.93758 -0.33810

H -6.66805 -1.81276 0.66438

H -6.71608 -2.84673 -0.75998

C -5.95428 1.51175 -1.79348

H -5.96632 2.50961 -1.35073

H -6.81520 1.47800 -2.48462

C -4.66865 1.33574 -2.60321

H -4.64098 0.32475 -3.02272

H -4.69519 2.03311 -3.46229

C -6.83006 1.03583 0.43097

H -7.20151 0.18610 1.00712

H -7.72656 1.60215 0.11887

C -5.99407 1.93279 1.34177

H -5.62536 2.79040 0.77110

H -6.65909 2.34371 2.12531

C -4.45551 -2.98910 0.90114

H -3.37192 -2.99029 1.01474

H -4.80255 -4.02442 0.72898

H -4.90387 -2.62849 1.83167

C -4.16506 -2.54381 -1.43268

H -4.36866 -1.84575 -2.24832

H -4.52138 -3.54424 -1.74056

H -3.08800 -2.56457 -1.26813

C -2.27195 1.02926 -2.50828

H -1.41359 1.04519 -1.83098

H -2.03618 1.62853 -3.40675

H -2.42936 -0.00855 -2.81366

C -3.26843 2.91984 -1.41534

H -4.10337 3.26523 -0.80327

H -3.19118 3.58275 -2.29659

H -2.35580 3.01674 -0.82965

C -3.96591 2.28000 2.56779

H -3.10381 1.78779 3.01840

H -4.49739 2.85249 3.34929

H -3.58831 2.97444 1.81493

C -5.21113 0.26262 2.92277

H -5.79304 0.68911 3.75979

H -4.30978 -0.20260 3.33088

H -5.80807 -0.52183 2.45476

C 0.84002 -2.65312 -1.18965

C -0.10908 -2.54807 -2.21943

H -1.08150 -2.14870 -1.95224

C 0.20231 -2.93185 -3.52206

H -0.54890 -2.84174 -4.30245

C 1.47282 -3.42470 -3.83481

H 1.71318 -3.72350 -4.85065

C 2.42919 -3.52918 -2.82349

H 3.42160 -3.91124 -3.04743

C 2.11160 -3.15027 -1.51664

H 2.85109 -3.25587 -0.72769

C 0.50073 -2.32156 0.24364

H 1.43110 -2.08672 0.76120

C 1.29065 -5.07297 1.38721

H 1.79071 -5.33881 0.44983

H 0.95261 -6.00386 1.85678

H 2.03540 -4.61711 2.04608

C -1.32013 -4.89390 -0.10733

H -2.19897 -4.31941 -0.39886

H -1.65769 -5.81379 0.38427

H -0.78668 -5.17494 -1.02028

C -1.06668 -3.53934 2.67275

H -0.36248 -3.25017 3.45752

H -1.63077 -4.40847 3.02912

H -1.76245 -2.70983 2.52303

C -0.46971 -1.14962 0.40170

O -1.70133 -1.37616 0.02461

**G**-Na

SCF E (BS-A) = -1958.358787301990

$\omega$1 = 10.47379455

$\omega$2 = 16.64515980

C 0.78258 3.70941 -0.50199

C 0.65939 4.02120 0.86410

H -0.06847 3.46758 1.44975

C 1.43929 5.01537 1.44671

H 1.32507 5.23848 2.50418

C 2.35863 5.73614 0.67709

H 2.96512 6.51375 1.13171

C 2.47670 5.45464 -0.68496

H 3.17974 6.01355 -1.29664

C 1.69348 4.45753 -1.26627

H 1.79023 4.24309 -2.32817

C -0.00107 2.62620 -1.12412

H 0.00659 2.65084 -2.22392

C -0.64805 1.67066 -0.46416

Si -3.66477 -0.22450 -2.00918

Na 1.26375 -0.95063 -0.17820

N 0.85839 -3.34089 -0.63044

N 1.50144 -0.28375 2.15364

N 3.23027 -0.19060 -1.46729

N 3.27110 -2.26242 0.73937

C 2.88260 -3.67746 0.78919

H 2.28527 -3.83051 1.69084

H 3.76336 -4.33657 0.89045

C 2.07300 -4.12880 -0.42985

H 2.68414 -4.03124 -1.33282

H 1.85415 -5.20747 -0.32040

C 3.54416 -1.72528 2.08000

H 4.19033 -0.85161 1.97497

H 4.10863 -2.44752 2.69490

C 2.27531 -1.32250 2.83562

H 1.62521 -2.19658 2.94778

H 2.56137 -1.01328 3.85843

C 4.38207 -2.02525 -0.18939

H 4.28157 -2.71730 -1.02782

H 5.35654 -2.25387 0.27738

C 4.43008 -0.59209 -0.72328

H 4.54284 0.10533 0.11201

H 5.33933 -0.48350 -1.34313

C 0.31629 -3.49106 -1.98045

H -0.51276 -2.79066 -2.10150

H -0.04314 -4.51522 -2.18498

H 1.08580 -3.24462 -2.71772

C -0.17913 -3.63965 0.35789

H 0.18883 -3.45359 1.37008

H -0.51796 -4.68978 0.30235

H -1.02340 -2.97250 0.18066

C 0.14391 -0.17955 2.69677

H -0.41772 0.55126 2.11115

H 0.14041 0.12534 3.75825

H -0.36375 -1.14255 2.60990

C 2.15599 1.02425 2.19893

H 3.14881 0.97710 1.74623

H 2.26884 1.39914 3.23221

H 1.56349 1.74101 1.62937

C 3.20181 1.26393 -1.64950

H 2.28544 1.54643 -2.16918

H 4.06823 1.63063 -2.22714

H 3.19177 1.76323 -0.67876

C 3.14204 -0.85927 -2.76673

H 3.99617 -0.61460 -3.42267

H 2.22076 -0.55245 -3.26887

H 3.10820 -1.94321 -2.64020

C -3.57264 0.26045 0.78250

C -3.14364 -0.88252 1.47647

H -2.29750 -1.42745 1.07850

C -3.77492 -1.28456 2.65216

H -3.42036 -2.16893 3.17554

C -4.85959 -0.56424 3.15751

H -5.35374 -0.88157 4.07096

C -5.30044 0.56957 2.47397

H -6.13960 1.14428 2.85604

C -4.66117 0.97681 1.30252

H -5.00476 1.86756 0.78307

C -2.91708 0.70293 -0.50438

H -3.15274 1.76326 -0.65781

C -5.40128 0.47105 -2.29052

H -6.01644 0.34787 -1.39294

H -5.90694 -0.0406 -3.11686

H -5.36751 1.53983 -2.52852

C -3.78574 -2.07861 -1.69275

H -2.78916 -2.48788 -1.51221

H -4.23193 -2.59773 -2.54830

H -4.39882 -2.28433 -0.81014

C -2.57470 0.12262 -3.51269

H -2.46539 1.20021 -3.67604

H -2.98722 -0.31870 -4.42640

H -1.57736 -0.29368 -3.34255

C -1.39806 0.53675 -0.55160

O -0.91740 -0.66589 -0.58904

[Na]OSiMe3

SCF E (BS-A) = -1341.401172530526

$\omega$1 = 29.74460738

$\omega$2 = 40.24752127

Si -3.42620 0.02611 0.20031

C -4.63594 -1.00699 1.25078

H -4.32099 -2.05737 1.28654

H -5.65933 -0.98132 0.85615

H -4.66778 -0.64088 2.28391

C -3.57064 -0.69508 -1.56996

H -3.13005 -0.02605 -2.32092

H -4.61776 -0.85227 -1.85813

H -3.05946 -1.66269 -1.64713

C -4.18031 1.77205 0.06355

H -4.23598 2.26032 1.04364

H -5.18987 1.76537 -0.36582

H -3.55019 2.3988 -0.57968

Na 0.09818 0.01484 0.15801

N 0.44933 1.07983 -2.01522

N 0.73817 -2.33617 0.12352

N 1.16680 1.27091 1.94996

N 2.63555 -0.05378 -0.28347

C 2.81431 0.32000 -1.69116

H 2.64456 -0.56774 -2.30414

H 3.85133 0.63847 -1.89884

C 1.86532 1.43311 -2.14681

H 2.03883 2.32835 -1.54212

H 2.12298 1.70623 -3.18681

C 3.06716 -1.42998 -0.01148

H 3.21611 -1.53106 1.06583

H 4.04550 -1.64862 -0.47557

C 2.06253 -2.48833 -0.47934

H 1.93823 -2.41872 -1.56456

H 2.49411 -3.48713 -0.28078

C 3.25617 0.91243 0.63169

H 3.18952 1.90323 0.17715

H 4.33389 0.71110 0.76429

C 2.59193 0.95033 2.01248

H 2.68837 -0.03015 2.48898

H 3.15174 1.66095 2.64887

C -0.41183 2.26417 -2.04928

H -1.44075 1.97519 -1.82040

H -0.39357 2.77470 -3.02810

H -0.08901 2.97332 -1.28290

C 0.02454 0.11210 -3.02675

H 0.62879 -0.79581 -2.96560

H 0.11071 0.51362 -4.05223

H -1.01535 -0.16700 -2.84664

C -0.29059 -3.06521 -0.61811

H -1.26777 -2.83785 -0.18589

H -0.13522 -4.15849 -0.59853

H -0.29744 -2.73505 -1.66046

C 0.70944 -2.71933 1.53465

H 1.44441 -2.14442 2.10237

H 0.92420 -3.79269 1.68309

H -0.27874 -2.49501 1.94344

C 0.44095 0.87272 3.15780

H -0.63117 0.96068 2.96507

H 0.71545 1.47982 4.03843

H 0.65283 -0.17605 3.38196

C 0.91582 2.67895 1.65152

H 1.28476 3.34973 2.44829

H -0.15981 2.83350 1.53503

H 1.40041 2.96703 0.71558

O -1.93242 0.03450 0.77916

TS(**G**-**4**)-Na

SCF E (BS-A) = -1958.322672673288

$\omega$1 = -220.35495539

$\omega$2 = 11.13648286

C -1.87317 3.40406 0.32804

C -1.43247 2.89753 1.56325

H -1.74399 1.90353 1.86514

C -0.62215 3.65692 2.40308

H -0.29838 3.24485 3.35482

C -0.23999 4.95039 2.03572

H 0.38949 5.54353 2.69216

C -0.69768 5.48149 0.82798

H -0.41994 6.49088 0.53806

C -1.51321 4.72079 -0.00793

H -1.86166 5.13656 -0.94975

C -2.66953 2.59358 -0.61291

H -3.33710 3.17195 -1.25639

C -2.63511 1.25910 -0.75255

Si -3.88340 -0.36649 -1.32222

Na 1.52161 -0.30325 -0.37139

N 2.47788 -2.21408 -1.56994

N 1.89401 -0.62229 2.01025

N 2.16613 1.83460 -1.39111

N 4.01931 -0.11527 -0.04641

C 4.60613 -1.36228 -0.55273

H 4.55876 -2.11220 0.23878

H 5.67698 -1.23880 -0.78946

C 3.89333 -1.89690 -1.79803

H 3.93886 -1.14717 -2.59397

H 4.44754 -2.77886 -2.16756

C 4.22890 0.05956 1.39742

H 4.04272 1.10848 1.63860

H 5.27809 -0.13698 1.68062

C 3.32231 -0.82260 2.26186

H 3.54346 -1.87701 2.06849

H 3.58049 -0.64315 3.32211

C 4.47361 1.05435 -0.81236

H 4.65935 0.73741 -1.84086

H 5.43835 1.43296 -0.43276

C 3.46255 2.20264 -0.81559

H 3.28102 2.53286 0.21212

H 3.91331 3.06337 -1.34360

C 1.76396 -2.38282 -2.83884

H 0.70603 -2.57328 -2.63896

H 2.15881 -3.22050 -3.43894

H 1.83942 -1.46671 -3.43084

C 2.31446 -3.41018 -0.74071

H 2.81911 -3.28358 0.21849

H 2.72048 -4.31376 -1.22816

H 1.25526 -3.56587 -0.53366

C 1.08933 -1.70912 2.57527

H 0.04173 -1.56363 2.30716

H 1.17259 -1.76354 3.67483

H 1.40891 -2.66604 2.15605

C 1.40959 0.66698 2.50632

H 1.96963 1.49118 2.05900

H 1.49083 0.75155 3.60464

H 0.36655 0.78883 2.21319

C 1.13537 2.82046 -1.04875

H 0.16780 2.47109 -1.40903

H 1.34723 3.81336 -1.48069

H 1.06120 2.92317 0.03514

C 2.23071 1.66996 -2.84321

H 2.52484 2.60188 -3.35795

H 1.24753 1.36862 -3.21422

H 2.94691 0.89155 -3.11635

C -2.33146 -2.04371 0.94276

C -1.53270 -2.86887 0.12448

H -1.24174 -2.50804 -0.85564

C -1.14493 -4.13925 0.54376

H -0.55703 -4.76842 -0.11948

C -1.52630 -4.62449 1.79763

H -1.22341 -5.61607 2.11997

C -2.31989 -3.82246 2.61987

H -2.63399 -4.18559 3.59471

C -2.71784 -2.55588 2.19473

H -3.33967 -1.94213 2.84162

C -2.77672 -0.71504 0.49252

H -3.46441 -0.23088 1.19600

C -4.99282 -1.92891 -0.99249

H -4.39190 -2.81895 -0.77786

H -5.63566 -2.14936 -1.85567

H -5.65245 -1.76884 -0.12909

C -2.80561 -0.89281 -2.80771

H -1.74389 -0.89801 -2.53691

H -2.92465 -0.17579 -3.62764

H -3.07096 -1.88837 -3.17921

C -5.30725 0.83231 -1.81288

H -5.78070 1.24700 -0.91430

H -6.08840 0.32142 -2.38836

H -4.92864 1.67745 -2.39737

C -1.82242 0.26507 -0.05015

O -0.60066 0.07526 -0.22422

**4**

SCF E (BS-A) = -1958.393031731940

$\omega$1 = 12.09021966

$\omega$2 = 23.85072962

Si -2.27742 2.14714 2.40107

Na 1.36443 -0.40069 0.24032

O -0.76444 -0.30368 0.06306

N 3.80634 -1.15405 0.38444

N 1.38271 -2.45707 1.58044

N 2.08311 -0.79475 -2.04183

N 2.60006 1.47685 1.25637

C 3.80539 -2.49551 0.98324

H 3.62002 -3.22223 0.18949

H 4.79321 -2.74975 1.40580

C 2.74429 -2.66539 2.07368

H 2.86805 -3.66506 2.53023

H 2.92277 -1.93798 2.87230

C 4.32202 -1.16318 -0.99103

H 5.21964 -1.79938 -1.08175

H 4.64460 -0.15089 -1.24304

C 3.28525 -1.62962 -2.01727

H 3.77203 -1.67754 -3.00914

H 2.97214 -2.65054 -1.77733

C 4.49359 -0.17234 1.23088

H 5.58856 -0.21098 1.09162

H 4.31390 -0.43717 2.27522

C 4.02698 1.26469 0.98867

H 4.65397 1.94250 1.59675

H 4.20625 1.53453 -0.05641

C 0.91395 -3.57023 0.75426

H -0.10495 -3.36465 0.42285

H 0.91754 -4.52678 1.30693

H 1.54270 -3.68697 -0.13179

C 0.43230 -2.21160 2.66682

H 0.78437 -1.37817 3.28092

H 0.29656 -3.09060 3.32103

H -0.52907 -1.93084 2.23274

C 2.31873 0.51250 -2.65509

H 1.41421 1.11636 -2.58123

H 2.59592 0.43033 -3.72113

H 3.12253 1.03994 -2.13618

C 0.96263 -1.46928 -2.70201

H 1.15162 -1.64846 -3.77515

H 0.06508 -0.85880 -2.59415

H 0.77528 -2.43058 -2.21675

C 2.30050 1.45861 2.68886

H 1.22942 1.61400 2.83396

H 2.84460 2.24534 3.24096

H 2.56059 0.49223 3.12666

C 2.14004 2.73623 0.66503

H 2.29499 2.71649 -0.41636

H 2.66505 3.61396 1.0813

H 1.06950 2.84709 0.84455

C -1.89785 0.29012 0.21634

C -1.81387 1.74643 0.61937

C -1.33612 2.70932 -0.1997

H -1.19466 3.70976 0.21204

C -0.95246 2.61572 -1.61846

C -1.40814 1.6009 -2.48224

H -2.07451 0.83799 -2.09845

C -1.03418 1.59056 -3.82364

H -1.40517 0.80346 -4.47352

C -0.19166 2.58015 -4.33776

H 0.09903 2.56343 -5.38370

C 0.26689 3.59397 -3.49517

H 0.91541 4.37450 -3.88232

C -0.11883 3.61346 -2.15560

H 0.22541 4.41583 -1.50772

C -3.15146 -0.27750 0.08337

H -4.00537 0.36806 0.27031

C -3.44669 -1.63456 -0.32397

C -2.45745 -2.55131 -0.76292

H -1.43416 -2.20373 -0.79317

C -2.79433 -3.84492 -1.15274

H -2.00836 -4.52007 -1.48602

C -4.12042 -4.28522 -1.12560

H -4.37700 -5.29633 -1.42763

C -5.11256 -3.39320 -0.70535

H -6.15219 -3.71111 -0.68099

C -4.78386 -2.09933 -0.31581

H -5.56969 -1.42014 0.00744

C -1.72379 0.73004 3.51843

H -0.63988 0.58887 3.48662

H -2.18936 -0.20330 3.18871

H -2.01360 0.91214 4.55893

C -4.14840 2.35335 2.56748

H -4.42343 2.59794 3.59969

H -4.66497 1.43000 2.28977

H -4.51680 3.15472 1.91887

C -1.43162 3.74971 2.95120

H -1.67580 3.96926 3.99645

H -1.75417 4.60782 2.35242

H -0.34162 3.67810 2.87194

**C**-Li + CO → **D**-Li

TS(**C**-**D**)-Li

SCF E (BS-A) = -1608.373963756615

$\omega$1 = -122.90465861

$\omega$2 = 20.41729576

O 0.41171 0.68451 3.36047

C -0.52514 1.22962 2.89731

Si -3.30977 2.16901 0.35159

N 2.22799 -0.77261 0.22747

N 2.29880 0.42497 -2.42759

N 3.25152 1.97476 0.52760

N 0.09635 -3.64725 1.53201

C 2.98137 -1.41516 -0.86649

H 2.35207 -2.19758 -1.29712

H 3.88516 -1.92081 -0.49642

C 3.39863 -0.44616 -1.98300

H 4.20503 0.20446 -1.63780

H 3.80539 -1.03674 -2.82323

C 3.04867 -0.37066 1.38346

H 3.67723 -1.19862 1.74999

H 2.35810 -0.10671 2.18795

C 3.97127 0.81041 1.07918

H 4.51593 1.07992 1.99946

H 4.72840 0.50853 0.35040

C 1.03781 -1.54727 0.63702

H 0.42368 -1.69440 -0.25457

H 0.44919 -0.92062 1.31079

C 1.32727 -2.89026 1.31670

H 2.06775 -3.47536 0.73418

H 1.78054 -2.69852 2.29424

C 1.23362 -0.33919 -3.08924

H 1.61502 -0.90660 -3.95455

H 0.46227 0.35051 -3.43514

H 0.76085 -1.03082 -2.39306

C 2.79809 1.46350 -3.33524

H 3.57232 2.05126 -2.83803

H 1.97716 2.13252 -3.60577

H 3.22195 1.04066 -4.26095

C 4.18339 2.89291 -0.13348

H 4.91968 3.31601 0.56933

H 3.62486 3.71870 -0.58222

H 4.72887 2.37183 -0.92414

C 2.52066 2.69796 1.58258

H 1.81867 2.03496 2.08176

H 1.95101 3.51399 1.13154

H 3.21012 3.11848 2.33276

C -0.39021 -4.26404 0.30421

H -0.58728 -3.51110 -0.46019

H -1.33721 -4.77380 0.50188

H 0.32104 -5.00703 -0.10833

C 0.26026 -4.64810 2.57708

H 1.01580 -5.41887 2.32824

H -0.69236 -5.15824 2.74982

H 0.56178 -4.16464 3.51082

C -2.18382 0.63498 0.58441

H -2.48292 0.24888 1.56923

C -2.43835 -0.42753 -0.44565

C -2.02470 -0.29258 -1.78315

H -1.51768 0.61592 -2.07869

C -2.23972 -1.31465 -2.70772

H -1.90633 -1.18572 -3.73435

C -2.88662 -2.49251 -2.32848

H -3.06232 -3.28242 -3.05251

C -3.30677 -2.63912 -1.00436

H -3.80614 -3.55112 -0.68938

C -3.07590 -1.62521 -0.07675

H -3.38559 -1.75914 0.95574

C -3.23768 2.80809 -1.42227

H -2.20672 3.05541 -1.69403

H -3.84553 3.71410 -1.52552

H -3.60848 2.06629 -2.13512

C -2.75055 3.52773 1.53467

H -2.51367 3.10522 2.51529

H -3.51948 4.29950 1.65009

H -1.84005 4.00869 1.16160

C -5.07150 1.62380 0.76562

H -5.37461 0.78205 0.13423

H -5.78864 2.43789 0.61267

H -5.14694 1.30296 1.81021

Li 1.64632 1.09443 -0.54468

O -0.14450 1.53451 -0.28590

C -0.71384 1.05552 0.72813

**D**-Li

SCF E (BS-A) = -1608.412820700183

$\omega$1 = 10.99563022

$\omega$2 = 17.62630165

O -0.07789 0.11305 3.45837

C -0.50180 0.42652 2.38065

Si -3.24730 2.16716 0.27237

N 2.45167 -0.70603 -0.11897

N 1.99608 1.18029 -2.32126

N 3.16429 1.92610 0.92711

N 0.81101 -3.93559 1.04241

C 3.01631 -0.93681 -1.46084

H 2.34237 -1.60532 -2.00135

H 3.98418 -1.45789 -1.40933

C 3.20614 0.34621 -2.28316

H 4.00507 0.95456 -1.85342

H 3.53447 0.06440 -3.29988

C 3.45489 -0.55308 0.94423

H 4.24280 -1.32173 0.88934

H 2.94155 -0.69862 1.89799

C 4.13333 0.81878 0.93412

H 4.81439 0.88318 1.80037

H 4.75913 0.91409 0.04290

C 1.38163 -1.65741 0.23986

H 0.58760 -1.54887 -0.50156

H 0.95229 -1.33710 1.19176

C 1.81457 -3.12373 0.36111

H 2.05445 -3.53195 -0.64321

H 2.73739 -3.18078 0.95011

C 0.92422 0.54882 -3.09870

H 1.21887 0.39372 -4.15032

H 0.03692 1.18433 -3.07184

H 0.65654 -0.41538 -2.66701

C 2.28715 2.50556 -2.87590

H 3.06470 2.99356 -2.28495

H 1.38471 3.12065 -2.83208

H 2.62511 2.45615 -3.92446

C 3.80616 3.18114 0.53018

H 4.57783 3.50368 1.24909

H 3.05135 3.96894 0.46022

H 4.28030 3.06571 -0.44781

C 2.53245 2.09501 2.24462

H 2.05905 1.16916 2.56627

H 1.75214 2.85539 2.17624

H 3.26737 2.39742 3.00917

C -0.47324 -3.93920 0.34892

H -0.90831 -2.93966 0.32445

H -1.17034 -4.58820 0.88613

H -0.39991 -4.30739 -0.69266

C 1.29898 -5.29163 1.24310

H 1.49111 -5.83049 0.29423

H 0.56435 -5.86783 1.81369

H 2.23338 -5.27048 1.81321

C -2.42313 0.55119 0.90358

H -2.94162 0.32350 1.84595

C -2.64426 -0.59902 -0.04902

C -1.89954 -0.68944 -1.23687

H -1.15670 0.07518 -1.41770

C -2.10118 -1.73717 -2.13217

H -1.51085 -1.78888 -3.04365

C -3.06021 -2.71835 -1.86650

H -3.22062 -3.53419 -2.56477

C -3.81067 -2.63747 -0.69293

H -4.55687 -3.39535 -0.47157

C -3.60363 -1.58831 0.20528

H -4.18813 -1.53852 1.12034

C -2.71534 2.59048 -1.48723

H -1.62701 2.68782 -1.50596

H -3.16947 3.52948 -1.82314

H -3.00365 1.80234 -2.18944

C -2.76178 3.55309 1.45612

H -3.02377 3.30049 2.48897

H -3.25663 4.49672 1.20197

H -1.67920 3.70297 1.41103

C -5.11966 1.89115 0.30851

H -5.39338 1.02484 -0.30320

H -5.65992 2.76118 -0.08083

H -5.47604 1.70512 1.32770

Li 1.50494 1.20570 -0.25246

O -0.17909 1.43626 0.28386

C -0.95443 0.83384 1.19989

**C’**-Li → **3’**

**C’**-Li

SCF E (BS-A) = -1495.058216266771

$\omega$1 = 12.83640333

$\omega$2 = 21.66121024

Si 2.10701 2.43646 0.15466

N -2.75987 -0.73859 0.06060

N -1.25592 -2.78728 -1.47188

N -0.87480 -1.88288 1.90699

N -4.29325 2.74424 0.11641

C -3.31229 -1.41423 -1.12007

H -3.09766 -0.78235 -1.98586

H -4.40901 -1.51657 -1.06480

C -2.72157 -2.80543 -1.34988

H -2.98414 -3.45654 -0.51201

H -3.19415 -3.24608 -2.24541

C -3.23025 -1.27264 1.34859

H -4.22665 -1.73193 1.26241

H -3.34263 -0.43622 2.04309

C -2.27528 -2.30596 1.96935

H -2.60191 -2.50288 3.00723

H -2.34848 -3.25571 1.43355

C -2.84972 0.72767 -0.03769

H -2.26710 1.01960 -0.91262

H -2.34082 1.15999 0.82960

C -4.27329 1.29762 -0.09030

H -4.75882 1.01735 -1.04651

H -4.87033 0.84337 0.70938

C -0.83595 -2.27631 -2.78475

H -1.21355 -2.91143 -3.60403

H 0.25448 -2.25020 -2.82604

H -1.18552 -1.25407 -2.92316

C -0.69805 -4.12610 -1.26722

H -1.00812 -4.51405 -0.29381

H 0.39315 -4.07015 -1.28553

H -1.02499 -4.84096 -2.04144

C 0.03391 -2.97067 2.27025

H -0.07284 -3.27425 3.32573

H 1.06356 -2.64650 2.09804

H -0.15842 -3.84176 1.64021

C -0.60191 -0.71183 2.74232

H -1.20964 0.13743 2.42594

H 0.44515 -0.43181 2.61722

H -0.80080 -0.90738 3.81014

C -3.61545 3.46717 -0.95552

H -2.55764 3.20013 -0.99621

H -3.67783 4.54180 -0.76161

H -4.06007 3.27324 -1.95095

C -5.66019 3.22360 0.27239

H -6.28414 3.05943 -0.62743

H -5.65310 4.29699 0.48492

H -6.13940 2.71234 1.11316

C 2.26518 0.77683 -0.80141

H 2.25953 1.09722 -1.85750

C 3.57201 0.11454 -0.46364

C 3.71558 -0.65678 0.70288

H 2.84863 -0.79620 1.33821

C 4.93665 -1.24662 1.02841

H 5.02181 -1.83923 1.93554

C 6.04741 -1.07837 0.19937

H 6.99926 -1.53397 0.45585

C 5.91897 -0.31865 -0.96482

H 6.77268 -0.18223 -1.62298

C 4.69514 0.26615 -1.29084

H 4.60297 0.85322 -2.20102

C 2.13149 2.15749 2.02203

H 1.23690 1.62000 2.34903

H 2.16016 3.11611 2.55198

H 3.00703 1.57670 2.32641

C 0.49559 3.29820 -0.31759

H 0.43570 3.46197 -1.39810

H 0.41151 4.26969 0.18236

H -0.36077 2.68272 -0.03108

C 3.57881 3.52194 -0.32168

H 4.52341 3.02385 -0.08296

H 3.55146 4.47707 0.21483

H 3.57875 3.73967 -1.39512

Li -0.61071 -1.21941 -0.12312

O 0.01876 0.23306 -1.22794

C 1.02712 -0.08482 -0.53331

TS-(**C’**-**3’**)-Li

SCF E (BS-A) = -1495.032869338116

$\omega$1 = -321.72591553

$\omega$2 = 21.57638655

Si 2.06839 1.85264 -0.14671

N -3.03220 -0.60836 0.15878

N -1.81827 -2.72634 -1.42507

N -0.86011 -1.61267 1.78390

N -4.52125 2.88469 0.25394

C -3.81681 -1.38137 -0.81764

H -3.82296 -0.81745 -1.75411

H -4.86897 -1.48883 -0.50983

C -3.24185 -2.77591 -1.06992

H -3.34208 -3.38404 -0.16657

H -3.84277 -3.27281 -1.85166

C -3.26706 -1.00060 1.56420

H -4.26967 -1.43138 1.69951

H -3.23898 -0.09672 2.17709

C -2.23452 -2.00015 2.10865

H -2.39127 -2.10175 3.19781

H -2.40389 -2.98961 1.67438

C -3.14230 0.84478 -0.05851

H -2.73648 1.05459 -1.05229

H -2.48704 1.34617 0.65802

C -4.55186 1.43415 0.08739

H -5.17862 1.13425 -0.77640

H -5.02492 1.01035 0.98092

C -1.60346 -2.19827 -2.77966

H -2.05128 -2.85031 -3.54789

H -0.53138 -2.09980 -2.95364

H -2.03832 -1.20113 -2.87235

C -1.18825 -4.04224 -1.30005

H -1.33344 -4.42755 -0.28739

H -0.11551 -3.94643 -1.48477

H -1.60270 -4.77617 -2.01131

C 0.09328 -2.68873 2.06351

H

0.15134 -2.92829 3.13850

H 1.08058 -2.37697 1.71477

H -0.19649 -3.59312 1.52245

C -0.42648 -0.38437 2.46171

H -1.11325 0.43542 2.24414

H 0.55422 -0.11274 2.06259

H -0.37775 -0.51452 3.55602

C -3.94405 3.56598 -0.90077

H -2.89692 3.28367 -1.03275

H -3.97551 4.64629 -0.73430

H -4.48456 3.34991 -1.84276

C -5.85395 3.39738 0.54371

H -6.57384 3.22718 -0.27984

H -5.80246 4.47437 0.72913

H -6.24928 2.91461 1.44293

C 2.76013 0.12425 -1.10142

H 2.75266 0.10278 -2.20414

C 4.11109 -0.05524 -0.50825

C 4.31215 -0.71457 0.71894

H 3.44919 -1.12461 1.23065

C 5.58691 -0.83418 1.26949

H 5.71344 -1.34313 2.22139

C 6.70075 -0.32745 0.59688

H 7.69505 -0.43503 1.01993

C 6.52176 0.30342 -0.63651

H 7.37942 0.69247 -1.17831

C 5.24539 0.43955 -1.17754

H 5.11449 0.93484 -2.13554

C 2.68878 2.11544 1.61961

H 2.04975 1.61539 2.35198

H 2.70280 3.18903 1.84562

H 3.70577 1.73449 1.74214

C 0.25890 2.40108 -0.33480

H -0.13229 2.07461 -1.30326

H 0.17278 3.49218 -0.27037

H -0.36157 1.95029 0.44222

C 3.01656 3.14698 -1.19609

H 4.09830 2.98899 -1.14844

H 2.80859 4.16089 -0.83241

H 2.71794 3.10318 -2.24963

Li -0.95807 -1.20395 -0.23871

O 0.54342 -0.61730 -1.08700

C 1.55675 -0.22610 -0.38917

**3’**-Li

SCF E (BS-A) = -1495.101201628154

$\omega$1 = 12.32501966

$\omega$2 = 22.98069371

Si -1.57987 -1.76754 1.56186

N 2.63889 0.33691 -0.45478

N 0.60605 2.04628 -1.60032

N 2.46741 2.57068 1.40735

N 2.70390 -3.44477 -0.82063

C 2.76482 0.80882 -1.84509

H 2.40381 0.02194 -2.51212

H 3.81580 0.97931 -2.11852

C 1.97865 2.10061 -2.12014

H 2.47384 2.94740 -1.63644

H 1.99582 2.30040 -3.20667

C 3.76547 0.68950 0.41607

H 4.74042 0.46447 -0.04647

H 3.69186 0.07032 1.31414

C 3.75087 2.16677 0.81192

H 4.59409 2.36505 1.49604

H 3.91841 2.78323 -0.07597

C 2.23211 -1.07178 -0.33781

H 1.32426 -1.19781 -0.93066

H 1.93022 -1.25045 0.69675

C 3.28530 -2.10469 -0.75778

H 3.75257 -1.81596 -1.71973

H 4.08903 -2.11507 -0.01449

C -0.22514 1.07297 -2.32223

H -0.32012 1.33256 -3.38975

H -1.21292 1.03975 -1.86111

H 0.20474 0.07342 -2.24675

C -0.02879 3.36680 -1.65339

H 0.55886 4.08519 -1.07546

H -1.02612 3.29894 -1.21332

H -0.12473 3.74441 -2.68491

C 2.33435 4.02933 1.42503

H 3.10009 4.51506 2.05246

H 1.34912 4.29874 1.81426

H 2.42528 4.42304 0.40896

C 2.31092 2.03565 2.76902

H 2.30760 0.94583 2.75113

H 1.34445 2.34829 3.16669

H 3.11301 2.38903 3.43802

C 1.95098 -3.65582 -2.05135

H 1.16222 -2.90739 -2.15380

H 1.46888 -4.63690 -2.02272

H 2.59038 -3.61000 -2.9539

C 3.70979 -4.48541 -0.65122

H 4.47255 -4.48811 -1.45316

H 3.22499 -5.46626 -0.64687

H 4.22208 -4.35474 0.30633

C -2.17948 0.95099 0.55855

H -1.90562 2.00725 0.63048

C -3.45443 0.67520 -0.08266

C -4.49747 1.62874 -0.03772

H -4.33551 2.55008 0.51653

C -5.71909 1.40703 -0.66481

H -6.49905 2.16157 -0.60104

C -5.95306 0.22020 -1.36654

H -6.90742 0.04546 -1.85379

C -4.92946 -0.72660 -1.44716

H -5.08087 -1.64312 -2.01182

C -3.69942 -0.49507 -0.83630

H -2.89805 -1.21459 -0.96110

C -0.54992 -2.05322 3.12319

H 0.50146 -1.82332 2.93339

H -0.62718 -3.09170 3.46466

H -0.88927 -1.40223 3.93576

C -0.96741 -3.00115 0.25284

H -1.34582 -2.78268 -0.75119

H -1.30016 -4.01292 0.51386

H 0.12571 -3.02039 0.20716

C -3.37844 -2.16317 2.00959

H -3.77346 -1.40642 2.69565

H -3.42699 -3.13394 2.51735

H -4.03938 -2.19546 1.14183

Li 1.00441 1.42918 0.36967

C -1.26092 0.06269 1.07872

O -0.06060 0.45134 1.45024

**2**Li + PhNCS → **7**

PhNCS

SCF E (BS-A) = -722.730399985260

$\omega$1 = 43.41549837

$\omega$2 = 60.02725200

C 1.97896 -0.16492 0.00048

S 3.54135 0.14201 -0.00078

N 0.82769 -0.47659 0.00147

C -0.52980 -0.20914 0.00087

C -1.43553 -1.27705 0.00017

C -0.98838 1.11731 0.00083

C -2.80273 -1.01306 -0.00079

H -1.05751 -2.29299 0.00030

C -2.35784 1.36345 -0.00011

H -0.27087 1.93041 0.00150

C -3.26784 0.30329 -0.00094

H -3.50605 -1.83931 -0.00141

H -2.71501 2.38820 -0.00022

H -4.33424 0.50314 -0.00168

TS(**2**-**E**)-Li

SCF E (BS-A) = -2104.450479466110

$\omega$1 = -201.44242925

$\omega$2 = 20.00694209

C -1.07859 -2.10927 0.61444

S 0.41549 -2.44644 1.17049

N -2.10997 -2.44743 0.06598

C -3.46100 -2.18638 -0.04155

C -4.07192 -2.22676 -1.30331

C -4.23168 -1.92707 1.10447

C -5.43047 -1.95198 -1.42070

H -3.46836 -2.45216 -2.17527

C -5.59194 -1.66306 0.97230

H -3.75239 -1.92458 2.07680

C -6.19553 -1.66401 -0.28738

H -5.89473 -1.96349 -2.40171

H -6.18129 -1.45016 1.85857

H -7.25469 -1.44974 -0.38435

Si -0.55682 1.04153 2.65040

N 2.86028 0.21989 -0.68528

N 0.29122 -0.53661 -2.11563

N 2.17888 3.33586 -0.10657

N 5.06550 -2.71334 0.38701

C 2.74564 -0.01212 -2.14415

H 3.00422 -1.04926 -2.37115

H 3.47404 0.59635 -2.69456

C 1.35870 0.29689 -2.69935

H 1.10128 1.33957 -2.48801

H 1.38568 0.17691 -3.79749

C 3.73911 1.36666 -0.37530

H 4.73824 1.20750 -0.81115

H 3.87359 1.39066 0.70945

C 3.26471 2.72991 -0.87410

H 4.14824 3.39753 -0.89861

H 2.93220 2.63837 -1.91242

C 3.34033 -0.98279 0.02925

H 2.64626 -1.79003 -0.20052

H 3.24964 -0.79763 1.10241

C 4.77793 -1.43769 -0.26631

H 4.95676 -1.49034 -1.35888

H 5.48184 -0.69948 0.12870

C 0.49219 -1.96465 -2.40916

H 0.59245 -2.14211 -3.49247

H -0.36872 -2.52389 -2.04109

H 1.37730 -2.35239 -1.90572

C -1.00568 -0.12486 -2.68335

H -1.15341 0.94411 -2.54112

H -1.81085 -0.64684 -2.16781

H -1.05597 -0.35616 -3.76001

C 1.58443 4.43363 -0.86377

H 2.30404 5.24849 -1.06645

H 0.74375 4.85316 -0.30537

H 1.20868 4.06252 -1.82047

C 2.63673 3.83463 1.18836

H 3.08585 3.03423 1.77696

H 1.78199 4.21623 1.75290

H 3.37955 4.64731 1.08373

C 4.40512 -3.83702 -0.26890

H 3.31943 -3.71859 -0.25030

H 4.64185 -4.76034 0.26701

H 4.72191 -3.96224 -1.32282

C 6.50153 -2.94828 0.47307

H 6.98944 -3.02695 -0.51758

H 6.69010 -3.88011 1.01455

H 6.97949 -2.13203 1.02288

C -1.47305 0.46006 1.13944

H -2.28376 -0.16962 1.50740

C -1.96930 1.36863 0.11249

C -1.19016 2.43758 -0.40956

H -0.18860 2.60450 -0.02058

C -1.67252 3.27058 -1.41897

H -1.05030 4.08222 -1.78317

C -2.94176 3.07762 -1.97015

H -3.30782 3.72416 -2.76164

C -3.73270 2.03832 -1.46625

H -4.73044 1.87164 -1.86535

C -3.26508 1.21570 -0.45029

H -3.90624 0.42923 -0.07149

C 1.33390 0.85911 2.56054

H 1.62366 -0.19377 2.61210

H 1.80879 1.38403 3.39711

H 1.73573 1.28469 1.63700

C -1.12914 -0.03413 4.10030

H -2.19937 0.09799 4.29412

H -0.58748 0.20454 5.02231

H -0.95482 -1.09166 3.87384

C -0.90176 2.86548 3.05127

H -0.61419 3.51425 2.21812

H -0.35060 3.19035 3.94144

H -1.96965 3.02728 3.23302

Li 0.42715 -0.06086 -0.06126

**E**-Li

SCF E (BS-A) = -2104.541318813156

$\omega$1 = 12.96751498

$\omega$2 = 17.75419857

C -1.26272 -0.74639 0.63996

S 0.10685 0.00870 1.45473

N -1.15560 -1.93619 0.14700

C -2.22596 -2.57432 -0.49574

C -2.64906 -2.20992 -1.78729

C -2.85545 -3.66426 0.13046

C -3.68208 -2.90124 -2.41842

H -2.17346 -1.36829 -2.27938

C -3.89370 -4.34473 -0.50218

H -2.52034 -3.95403 1.12120

C -4.31484 -3.96985 -1.78045

H -3.99783 -2.59743 -3.41284

H -4.37672 -5.17425 0.00673

H -5.12243 -4.50333 -2.27179

Si -3.25647 0.38099 2.39176

N 3.66392 0.40469 -0.37996

N 1.38672 0.54731 -2.15286

N 2.21351 2.77250 0.55602

N 5.57424 -2.54267 1.12393

C 3.82370 0.19001 -1.83259

H 3.80192 -0.88530 -2.01993

H 4.80171 0.54444 -2.18881

C 2.72705 0.85668 -2.66515

H 2.84299 1.94363 -2.64557

H 2.84101 0.54486 -3.71847

C 4.37354 1.59111 0.12813

H 5.34382 1.73519 -0.37137

H 4.59192 1.42242 1.18517

C 3.56344 2.88411 -0.01857

H 4.13174 3.70864 0.44679

H 3.45347 3.13851 -1.07637

C 3.92929 -0.81820 0.40465

H 3.20329 -1.56722 0.07970

H 3.69964 -0.61084 1.45345

C 5.36059 -1.35884 0.29611

H 5.60798 -1.56068 -0.76554

H 6.05705 -0.58414 0.63654

C 1.03777 -0.86938 -2.33401

H 1.00215 -1.14576 -3.40062

H 0.07175 -1.06508 -1.87087

H 1.76183 -1.51029 -1.83027

C 0.38141 1.39406 -2.79490

H 0.60925 2.44692 -2.61100

H -0.60125 1.18437 -2.36884

H 0.33697 1.23202 -3.88476

C 1.38528 3.92462 0.17517

H 1.82570 4.87888 0.50792

H 0.40099 3.82166 0.63000

H 1.26785 3.95564 -0.91090

C 2.25700 2.66009 2.02212

H 2.79643 1.75964 2.32034

H 1.23846 2.56591 2.40070

H 2.74381 3.53441 2.48432

C 4.81592 -3.69640 0.64831

H 3.74196 -3.50872 0.70464

H 5.03250 -4.55822 1.28532

H 5.06639 -3.97146 -0.39497

C 6.99379 -2.86611 1.19658

H 7.43127 -3.11914 0.21122

H 7.14327 -3.72293 1.86007

H 7.54908 -2.01637 1.60510

C -2.59325 0.01199 0.62875

H -3.34575 -0.69140 0.25642

C -2.64796 1.20620 -0.30092

C -1.87966 2.36397 -0.10425

H -1.19421 2.38503 0.73325

C -1.98513 3.45119 -0.97112

H -1.38861 4.34106 -0.79357

C -2.85631 3.40826 -2.06048

H -2.93774 4.25709 -2.73272

C -3.62651 2.26389 -2.27029

H -4.31161 2.21395 -3.11171

C -3.52279 1.17926 -1.39890

H -4.12149 0.29003 -1.57179

C -2.49283 1.88674 3.23019

H -1.41029 1.76419 3.30707

H -2.90918 2.00558 4.23716

H -2.70172 2.80295 2.66969

C -2.98476 -1.17624 3.42004

H -3.41159 -2.04984 2.91560

H -3.45355 -1.09143 4.40639

H -1.91549 -1.35869 3.5605

C -5.10813 0.69430 2.16403

H -5.27806 1.53680 1.48511

H -5.58993 0.93060 3.11909

H -5.61158 -0.18199 1.74178

Li 1.56911 0.87917 -0.12527

TS(**E**-**7**)-Li

SCF E (BS-A) = -2104.519588704812

$\omega$1 = -122.60030978

$\omega$2 = 10.36933335

C 1.41557 -0.51201 -0.20888

S -0.14372 -0.55232 -0.92245

N 2.18337 -1.57330 0.04048

C 1.76126 -2.92299 -0.05596

C 1.59352 -3.67840 1.10852

C 1.56633 -3.52163 -1.30513

C 1.22747 -5.02136 1.02509

H 1.75720 -3.20342 2.07055

C 1.19881 -4.86356 -1.38430

H 1.69956 -2.92494 -2.20033

C 1.02871 -5.61796 -0.22128

H 1.10003 -5.60226 1.93380

H 1.04683 -5.32196 -2.35694

H 0.74617 -6.66421 -0.28635

Si 3.93910 -0.70468 0.23368

N -3.22189 0.71381 0.49907

N -0.99225 1.10891 2.38758

N -1.77261 3.03788 -0.48736

N -5.21962 -2.05550 -1.18914

C -3.37750 0.50169 1.94991

H -3.22188 -0.56150 2.14677

H -4.39785 0.73539 2.29035

C -2.39289 1.31211 2.79481

H -2.61106 2.37891 2.70644

H -2.54115 1.04671 3.85611

C -3.94396 1.88993 -0.02178

H -4.89880 2.05058 0.50144

H -4.19451 1.68781 -1.06569

C -3.13303 3.18675 0.04945

H -3.69044 3.97610 -0.48513

H -3.04091 3.51974 1.08638

C -3.50675 -0.50922 -0.27785

H -2.80461 -1.27324 0.06125

H -3.26217 -0.31057 -1.32555

C -4.95256 -1.01476 -0.20047

H -5.17806 -1.36138 0.82843

H -5.63511 -0.18266 -0.40723

C -0.52959 -0.24817 2.72042

H -0.63679 -0.45952 3.79683

H 0.51981 -0.34874 2.44275

H -1.08529 -0.99213 2.14941

C -0.12630 2.10388 3.03149

H -0.45865 3.11010 2.76555

H 0.89725 1.97737 2.67553

H -0.13282 2.01251 4.12969

C -0.96828 4.22726 -0.18011

H -1.38561 5.13621 -0.64399

H 0.04927 4.08441 -0.54092

H -0.93623 4.37914 0.90191

C -1.77838 2.80671 -1.94094

H -2.28183 1.86795 -2.17821

H -0.74702 2.72833 -2.28660

H -2.28083 3.62659 -2.48031

C -4.44046 -3.26635 -0.94751

H -3.37066 -3.06365 -1.03093

H -4.69467 -4.01518 -1.70277

H -4.63329 -3.70782 0.04957

C -6.64292 -2.36237 -1.24320

H -7.03608 -2.77236 -0.29285

H -6.83146 -3.09844 -2.03034

H -7.20949 -1.45650 -1.48047

C 2.20593 0.64715 0.25052

H 2.12978 0.73083 1.33789

C 2.20370 1.95654 -0.40460

C 1.95641 2.12084 -1.78497

H 1.67184 1.25595 -2.37300

C 2.08036 3.36590 -2.39847

H 1.88643 3.45509 -3.46414

C 2.45954 4.49274 -1.66301

H 2.56072 5.45947 -2.14617

C 2.70746 4.35149 -0.29539

H 3.00284 5.21393 0.29608

C 2.57318 3.10928 0.32084

H 2.77828 3.01472 1.38413

C 4.85785 0.40316 1.50748

H 4.76087 1.46371 1.25463

H 5.92298 0.15087 1.56913

H 4.43581 0.26481 2.51183

C 4.53223 -0.35052 -1.53094

H 3.99332 -0.97442 -2.25228

H 5.59828 -0.58539 -1.62617

H 4.38021 0.69494 -1.81223

C 4.77562 -2.38045 0.69053

H 4.48614 -2.73172 1.68912

H 5.86600 -2.26317 0.69408

H 4.52329 -3.18067 -0.01356

Li -1.12709 1.22864 0.29933

**7**

SCF E (BS-A) = -2104.555538568558

$\omega$1 = 11.16290398

$\omega$2 = 20.3635794

Coords

**2**Na + PhNCS → **8**

TS(**2**-**H**)-Na

SCF E (BS-A) = -2259.224936485524

$\omega$1 = -446.30968853

$\omega$2 = 13.02315076

Si -2.79047 1.93521 1.31531

Na 1.94439 -0.05682 0.05922

N 3.57909 1.24849 1.50716

N 3.24009 -0.63638 -2.12101

N 1.03466 -1.86284 1.58378

N 3.93560 -1.61012 0.72222

C 4.99626 -0.81054 1.35158

H 5.62272 -0.37858 0.57057

H 5.66741 -1.43959 1.96027

C 4.44367 0.30824 2.23217

H 3.85484 -0.12372 3.04612

H 5.29183 0.83290 2.70838

C 4.38045 -2.22077 -0.53830

H 3.65315 -2.98720 -0.81830

H 5.34585 -2.74217 -0.41001

C 4.51686 -1.21594 -1.67942

H 5.16628 -0.39371 -1.36859

H 5.03270 -1.71107 -2.52131

C 3.42408 -2.62803 1.65301

H 3.53866 -2.25350 2.67180

H 4.02782 -3.54977 1.60178

C 1.96270 -2.98979 1.41231

H 1.83736 -3.36305 0.39019

H 1.69862 -3.82377 2.08867

C 2.84692 2.09295 2.45819

H 2.19140 2.77266 1.91384

H 3.52322 2.68983 3.09375

H 2.22621 1.46730 3.10481

C 4.36342 2.08750 0.59486

H 4.93095 1.46706 -0.09966

H 5.07574 2.73375 1.13668

H 3.69196 2.71246 0.00691

C 3.48836 0.58520 -2.90001

H 2.53629 1.02352 -3.19908

H 4.09709 0.38628 -3.79897

H 4.00806 1.32000 -2.28185

C 2.50721 -1.59370 -2.95899

H 2.15853 -2.44433 -2.36950

H 3.13364 -1.97188 -3.78541

H 1.63786 -1.10138 -3.39193

C -0.31897 -2.28846 1.20581

H -0.98982 -1.42829 1.19975

H -0.71793 -3.04685 1.90160

H -0.30104 -2.71355 0.20280

C 1.00966 -1.38671 2.97126

H 0.75923 -2.19655 3.67880

H 0.25836 -0.60269 3.06765

H 1.97361 -0.96752 3.26013

C -0.94315 2.04487 -0.88790

C 0.10997 2.62418 -0.09099

H -0.01287 2.66393 0.98789

C 1.21404 3.25395 -0.66163

H 1.91424 3.77174 -0.01020

C 1.41561 3.27985 -2.04697

H 2.27497 3.77885 -2.48298

C 0.45032 2.64796 -2.85057

H 0.56946 2.64831 -3.93309

C -0.67225 2.05022 -2.30220

H -1.41798 1.61613 -2.96165

C -2.15028 1.54539 -0.35202

H -2.88774 1.23392 -1.09234

C -2.75273 3.80434 1.70326

H -1.76366 4.23478 1.51603

H -3.02326 4.01532 2.74528

H -3.46288 4.33405 1.05829

C -1.83742 1.11050 2.75257

H -1.96748 0.02318 2.72004

H -2.18985 1.46139 3.73014

H -0.76411 1.32372 2.68975

C -4.59672 1.39549 1.51685

H -5.22329 1.79179 0.71166

H -4.99948 1.76805 2.46622

H -4.70605 0.30817 1.51015

C -2.04745 -1.41675 -1.41768

S -0.67206 -1.22881 -2.19987

N -3.03141 -1.84169 -0.86838

C -4.35423 -1.60564 -0.54371

C -5.12766 -0.70888 -1.29710

C -4.92707 -2.30965 0.52356

C -6.46648 -0.52036 -0.97081

H -4.66789 -0.17211 -2.11885

C -6.26678 -2.10478 0.84250

H -4.31228 -3.00109 1.08907

C -7.04116 -1.21307 0.09742

H -7.06246 0.17723 -1.55067

H -6.70702 -2.64473 1.67478

H -8.08527 -1.05749 0.34820

**H**-Na

SCF E (BS-A) = -2259.297752328294

$\omega$1 = 19.03772362

$\omega$2 = 23.85722060

Si -3.08234 0.71673 1.51195

Na 2.09901 -0.24626 -0.10424

N 3.01881 1.47008 1.48491

N 3.36588 -0.20814 -2.25042

N 1.83834 -2.47221 0.87995

N 4.44446 -1.00647 0.42943

C 5.10356 0.08156 1.16322

H 5.51191 0.78690 0.43696

H 5.96738 -0.28985 1.74167

C 4.17740 0.82800 2.12387

H 3.78692 0.13169 2.87341

H 4.78566 1.56983 2.67365

C 5.12659 -1.29467 -0.84119

H 4.83160 -2.29378 -1.16689

H 6.22213 -1.32802 -0.70974

C 4.79667 -0.28965 -1.94622

H 5.12829 0.70915 -1.64452

H 5.38941 -0.5532 -2.84168

C 4.29918 -2.21602 1.25342

H 4.12207 -1.90727 2.28599

H 5.23228 -2.80559 1.26627

C 3.15194 -3.11862 0.79424

H 3.31078 -3.40939 -0.24821

H 3.18256 -4.05245 1.38484

C 2.12093 1.99367 2.51980

H 1.25276 2.46254 2.05404

H 2.61523 2.74188 3.16266

H 1.76882 1.17340 3.15223

C 3.41400 2.56155 0.58800

H 4.04859 2.18780 -0.21666

H 3.96168 3.35984 1.11849

H 2.51955 2.99221 0.13190

C 3.06584 0.98886 -3.04218

H 1.98883 1.04887 -3.21067

H 3.58011 0.98445 -4.01900

H 3.37539 1.88249 -2.49324

C 2.88261 -1.40030 -2.95569

H 3.06926 -2.29781 -2.36194

H 3.37507 -1.52790 -3.93575

H 1.80305 -1.31449 -3.09686

C 0.83898 -3.17794 0.06518

H -0.09331 -2.60896 0.02956

H 0.63745 -4.19460 0.44555

H 1.19868 -3.25623 -0.96322

C 1.38043 -2.37321 2.26672

H 1.29724 -3.36369 2.74844

H 0.40137 -1.89489 2.29154

H 2.06770 -1.76320 2.85909

C -2.05316 2.34910 -0.52506

C -0.88160 2.72760 0.14931

H -0.37764 1.96671 0.73492

C -0.38734 4.02384 0.06766

H 0.51431 4.29951 0.60827

C -1.05740 4.98042 -0.70384

H -0.67505 5.99464 -0.77081

C -2.22310 4.62182 -1.37690

H -2.75327 5.35613 -1.97651

C -2.71683 3.31601 -1.28299

H -3.62704 3.04436 -1.81137

C -2.60611 0.95663 -0.35899

H -3.56647 0.90882 -0.88127

C -3.13538 2.39135 2.39391

H -2.15191 2.85687 2.48554

H -3.54869 2.25347 3.39996

H -3.77980 3.09683 1.85948

C -1.76042 -0.35272 2.32961

H -1.70930 -1.32767 1.83876

H -1.95718 -0.50396 3.39606

H -0.78206 0.13216 2.22665

C -4.81489 0.00043 1.71697

H -5.53599 0.59367 1.14415

H -5.10680 0.05894 2.77216

H -4.90128 -1.03544 1.39179

C -1.72818 -0.18874 -0.92800

S -0.12998 0.18169 -1.54248

N -2.13655 -1.41739 -0.98304

C -3.43290 -1.86745 -0.76631

C -4.53565 -1.40140 -1.51297

C -3.64526 -2.94382 0.11872

C -5.80098 -1.95510 -1.33756

H -4.37987 -0.61738 -2.24704

C -4.91439 -3.48836 0.29313

H -2.79377 -3.33675 0.66562

C -6.00580 -2.99434 -0.42712

H -6.63406 -1.57467 -1.92264

H -5.05273 -4.30812 0.99288

H -6.99431 -3.42182 -0.29254

TS(**H**-**8**)-Na

SCF E (BS-A) = -2259.280655567467

$\omega$1 = -197.94696276

$\omega$2 = 24.81652781

Si -3.79764 -0.45232 0.90781

Na 1.71390 0.24603 -0.04618

N 1.93555 1.74968 1.89224

N 2.98177 1.12354 -1.96246

N 2.39607 -2.11165 0.35167

N 4.22786 0.23792 0.61761

C 4.39087 1.28737 1.63050

H 4.54056 2.24044 1.11950

H 5.29774 1.12518 2.23837

C 3.19130 1.39925 2.57181

H 3.03368 0.44203 3.07880

H 3.43149 2.13458 3.36087

C 4.99626 0.52223 -0.60151

H 5.13231 -0.41369 -1.14646

H 6.01096 0.88613 -0.36388

C 4.31457 1.53999 -1.51567

H 4.20012 2.49002 -0.98419

H 4.98283 1.74351 -2.37245

C 4.53437 -1.09187 1.15969

H 4.20664 -1.11928 2.20119

H 5.62244 -1.27937 1.17820

C 3.85651 -2.22273 0.38678

H 4.21021 -2.22195 -0.64854

H 4.17937 -3.18615 0.82259

C 0.79706 1.55241 2.79726

H -0.1314 1.81105 2.28529

H 0.87355 2.17097 3.70743

H 0.74233 0.50232 3.09788

C 1.95394 3.13542 1.41282

H 2.77139 3.28759 0.70627

H 2.06934 3.85948 2.23746

H 1.02139 3.34973 0.89098

C 2.24750 2.25615 -2.53089

H 1.23613 1.93716 -2.79137

H 2.73983 2.66746 -3.42911

H 2.16763 3.05406 -1.78706

C 3.04366 0.03399 -2.94088

H 3.54237 -0.83806 -2.51373

H 3.58592 0.32964 -3.85595

H 2.02849 -0.26170 -3.21423

C 1.83050 -2.98818 -0.68106

H 0.75729 -2.82058 -0.75498

H 2.01473 -4.05459 -0.46859

H 2.27535 -2.74184 -1.64844

C 1.79717 -2.41730 1.65223

H 2.01262 -3.44952 1.98044

H 0.71474 -2.30356 1.58284

H 2.16757 -1.73204 2.41881

C -2.78525 2.26650 -0.70180

C -1.62702 2.83340 -0.12701

H -0.81929 2.17106 0.16463

C -1.50754 4.20954 0.05227

H -0.60931 4.61976 0.50675

C -2.53382 5.07475 -0.33422

H -2.43761 6.14610 -0.18733

C -3.68554 4.53478 -0.91107

H -4.49541 5.18902 -1.22291

C -3.80480 3.16034 -1.09353

H -4.70833 2.75456 -1.54100

C -3.00951 0.82854 -0.78987

H -3.86454 0.56609 -1.41607

C -5.49735 0.42524 0.72283

H -5.37351 1.50196 0.57626

H -6.13006 0.25700 1.60276

H -6.04247 0.03358 -0.14549

C -2.81518 0.34135 2.32160

H -3.35151 0.22850 3.27056

H -2.65358 1.40820 2.14143

H -1.83750 -0.13929 2.43340

C -4.48584 -2.12127 1.58696

H -4.96958 -2.70172 0.79062

H -5.25472 -1.90906 2.33889

H -3.74521 -2.77802 2.04901

C -1.98461 -0.19357 -0.89576

S -0.44616 -0.09930 -1.66463

N -2.45393 -1.26373 -0.21175

C -1.96628 -2.58468 -0.35373

C -1.92510 -3.20376 -1.61071

C -1.55485 -3.30616 0.77276

C -1.47373 -4.51519 -1.73422

H -2.24474 -2.64310 -2.48169

C -1.11615 -4.62482 0.64859

H -1.58057 -2.82427 1.74322

C -1.07102 -5.23409 -0.60479

H -1.44451 -4.98161 -2.71444

H -0.80172 -5.17093 1.53288

H -0.72718 -6.25900 -0.70341

**8**

SCF E (BS-A) = -2259.321601321450

$\omega$1 = 16.52039750

$\omega$2 = 32.45155976

Si -3.38629 0.93271 1.64956

Na 1.51752 -0.53355 -0.15043

N 2.50398 0.97025 1.61604

N 3.09653 -1.04716 -1.93284

N 0.61374 -2.72278 0.70205

N 3.43738 -1.824 0.95861

C 4.21352 -0.86071 1.74895

H 4.88144 -0.32051 1.0754

H 4.86508 -1.37204 2.47966

C 3.33553 0.14066 2.49636

H 2.66489 -0.39346 3.17642

H 3.98645 0.76823 3.13256

C 4.22969 -2.38935 -0.14119

H 3.74687 -3.31004 -0.4727

H 5.24003 -2.67911 0.19777

C 4.37518 -1.44251 -1.33573

H 4.88595 -0.52713 -1.02019

H 5.03632 -1.9259 -2.07882

C 2.85569 -2.86502 1.81633

H 2.519 -2.38983 2.73992

H 3.61364 -3.61089 2.11654

C 1.67877 -3.60429 1.17879

H 2.03 -4.18129 0.31784

H 1.30484 -4.34425 1.91152

C 1.48881 1.67123 2.40749

H 0.85307 2.2557 1.74325

H 1.93803 2.34754 3.15586

H 0.86554 0.94356 2.93135

C 3.31646 1.94723 0.88307

H 4.01862 1.43993 0.21709

H 3.89469 2.5945 1.5657

H 2.66906 2.57318 0.2701

C 3.2659 0.09419 -2.83433

H 2.29142 0.37758 -3.23749

H 3.94454 -0.1302 -3.67576

H 3.66496 0.94836 -2.2808

C 2.44595 -2.15342 -2.63962

H 2.25989 -2.98488 -1.9578

H 3.05464 -2.52288 -3.48359

H 1.47898 -1.81587 -3.01881

C -0.30048 -3.44176 -0.18858

H -1.01009 -2.73317 -0.61762

H -0.84484 -4.2469 0.33719

H 0.26634 -3.8901 -1.00923

C -0.1427 -2.10129 1.79068

H -0.66465 -2.84645 2.41886

H -0.88448 -1.41966 1.36463

H 0.51971 -1.52128 2.43975

C -0.08299 2.96576 -0.65157

C 0.80152 2.68262 -1.71934

H 0.64911 1.76903 -2.27725

C 1.8294 3.56089 -2.05939

H 2.48126 3.31033 -2.89291

C 2.02972 4.74951 -1.35499

H 2.83467 5.42671 -1.62387

C 1.16112 5.05634 -0.30213

H 1.28867 5.97922 0.25741

C 0.12935 4.18847 0.03508

H -0.53843 4.4477 0.85378

C -1.18819 2.13108 -0.21113

H -1.82738 2.64207 0.50057

C -4.45277 2.43583 1.23445

H -3.88238 3.25946 0.79757

H -4.95977 2.80993 2.13116

H -5.22405 2.14821 0.51163

C -2.08415 1.34364 2.94952

H -2.58411 1.56983 3.89808

H -1.4524 2.19223 2.6854

H -1.43624 0.47683 3.11417

C -4.53146 -0.35189 2.41945

H -5.32606 -0.66704 1.73941

H -4.99908 0.09522 3.30487

H -3.99203 -1.24638 2.74402

C -1.53898 0.84029 -0.52491

S -0.72993 -0.19758 -1.68911

N -2.63162 0.2457 0.18685

C -3.45835 -0.66998 -0.54639

C -4.16286 -0.23178 -1.67534

C -3.61767 -1.9908 -0.11796

C -5.00192 -1.10241 -2.36617

H -4.02775 0.79246 -2.0073

C -4.46609 -2.86052 -0.8035

H -3.0672 -2.33125 0.7509

C -5.1592 -2.42125 -1.93174

H -5.53767 -0.75101 -3.24311

H -4.5781 -3.88445 -0.45873

H -5.81593 -3.09912 -2.46844

**2**Na + *t*BuNCS → **9**

*t*BuNCS

SCF E (BS-A) = -648.939225041767

$\omega$1 = 14.53937620

$\omega$2 = 54.06321940

C 1.15675 0.00035 -0.17807

S 2.74544 -0.00005 0.01945

N -0.00893 0.00077 -0.40829

C -1.40155 -0.00004 0.01308

C -1.64465 -1.26573 0.85008

C -1.64689 1.26715 0.84717

C -2.27659 -0.00225 -1.24730

H -1.43171 -2.16299 0.26358

H -1.00779 -1.26862 1.73859

H -2.69004 -1.29846 1.16991

H -1.43573 2.16344 0.25853

H -2.69227 1.29866 1.16712

H -1.00986 1.27332 1.73554

H -3.33195 -0.00280 -0.96222

H -2.08016 0.88466 -1.85518

H -2.07869 -0.89026 -1.85310

TS(**2**-**9**)-Na

SCF E (BS-A) = -2185.425571619270

$\omega$1 = -205.85307289

$\omega$2 = 17.90695668

Si 2.67774 1.15094 -2.03868

Na -1.57582 -0.24832 -0.10228

N -3.10549 1.46701 -1.27336

N -2.28004 -0.35563 2.34061

N -1.62384 -2.44765 -1.32042

N -4.05303 -1.10082 -0.05013

C -4.95171 -0.07114 -0.59028

H -5.24436 0.59723 0.22114

H -5.88879 -0.51396 -0.96717

C -4.30598 0.74233 -1.70819

H -4.01323 0.07417 -2.52411

H -5.05947 1.43478 -2.12574

C -4.34023 -1.37702 1.36497

H -3.92447 -2.35521 1.61537

H -5.42702 -1.44975 1.54336

C -3.74732 -0.32386 2.30011

H -4.05437 0.67186 1.96979

H -4.17475 -0.46084 3.30973

C -4.09704 -2.32296 -0.86476

H -4.29452 -2.03879 -1.90048

H -4.93795 -2.97176 -0.56521

C -2.81166 -3.14245 -0.80280

H -2.59909 -3.41669 0.23603

H -2.98214 -4.08756 -1.35101

C -2.38322 1.99000 -2.43859

H -1.48407 2.51082 -2.10346

H -2.99474 2.69540 -3.02716

H -2.08143 1.16689 -3.09160

C -3.44688 2.57811 -0.37903

H -3.98210 2.21770 0.49987

H -4.07953 3.33068 -0.88099

H -2.53066 3.05639 -0.03523

C -1.73921 0.90325 2.87507

H -0.64858 0.87930 2.84237

H -2.06333 1.08947 3.91338

H -2.06077 1.74353 2.25602

C -1.82132 -1.48192 3.16198

H -2.12401 -2.43265 2.71652

H -2.23117 -1.43112 4.18587

H -0.73328 -1.47588 3.22105

C -0.44028 -3.28413 -1.08281

H 0.45421 -2.76591 -1.43109

H -0.51498 -4.25375 -1.60495

H -0.32259 -3.46091 -0.01256

C -1.73693 -2.16506 -2.75494

H -1.9078 -3.08088 -3.34765

H -0.81567 -1.69249 -3.09906

H -2.55789 -1.47352 -2.95217

C 1.82287 2.22160 0.50578

C 0.69725 2.89729 -0.06695

H 0.54670 2.84500 -1.14133

C -0.14010 3.71634 0.68469

H -0.91905 4.27944 0.17430

C 0.03269 3.87484 2.06397

H -0.61949 4.52184 2.64216

C 1.09666 3.18497 2.66743

H 1.26084 3.28048 3.73935

C 1.95363 2.39049 1.92286

H 2.77363 1.87746 2.42059

C 2.74303 1.44082 -0.24158

H 3.66064 1.19311 0.28041

C 2.74838 2.71313 -3.13464

H 1.97327 3.43781 -2.86356

H 2.62629 2.47138 -4.19809

H 3.71497 3.21534 -3.01377

C 1.08987 0.22789 -2.56811

H 1.05252 0.05027 -3.64988

H 0.19228 0.78836 -2.29100

H 1.05456 -0.73770 -2.05460

C 4.10732 0.04221 -2.61452

H 5.08485 0.47233 -2.37107

H 4.07313 -0.10279 -3.70042

H 4.04082 -0.94120 -2.14102

C 2.43264 -1.11548 0.69577

S 0.89972 -1.01386 1.23713

N 3.47676 -1.65993 0.46505

C 4.88062 -1.84537 0.78317

C 4.93739 -2.50406 2.17468

H 4.40618 -3.45948 2.17059

H 5.97881 -2.68302 2.45926

H 4.47775 -1.85378 2.92396

C 5.61907 -0.49853 0.80685

H 6.67233 -0.66985 1.04965

H 5.55895 -0.00133 -0.16145

H 5.19160 0.16314 1.56393

C 5.49501 -2.77924 -0.26892

H 6.53719 -2.98842 -0.01200

H 4.94888 -3.72576 -0.30746

H 5.46873 -2.31996 -1.25945

**9**

SCF E (BS-A) = -2185.501756351532

$\omega$1 = 16.34706218

$\omega$2 = 38.43835045

Si 3.09650 -1.22008 1.78398

Na -1.59089 0.18067 0.09001

N -2.64040 -2.03328 -0.01658

N -2.26443 1.67401 -1.80951

N -1.99316 1.13453 2.34326

N -4.12872 0.56511 0.26611

C -4.76892 -0.70503 -0.09651

H -4.81492 -0.76504 -1.18569

H -5.81514 -0.7466 0.25463

C -4.0295 -1.92976 0.44299

H -4.01073 -1.89321 1.53650

H -4.60836 -2.83203 0.17239

C -4.48683 1.64071 -0.66864

H -4.28165 2.59629 -0.18176

H -5.56859 1.63659 -0.89035

C -3.7142 1.57298 -1.98613

H -3.92198 0.6208 -2.48487

H -4.09861 2.36397 -2.65675

C -4.39977 0.94548 1.65918

H -4.51534 0.03506 2.25033

H -5.35761 1.48649 1.74780

C -3.29465 1.80891 2.27107

H -3.16287 2.71204 1.66699

H -3.62851 2.14593 3.27000

C -1.90368 -3.01036 0.78915

H -0.86749 -3.04981 0.45079

H -2.33186 -4.02548 0.71378

H -1.91821 -2.70695 1.83954

C -2.55454 -2.38421 -1.43752

H -3.04284 -1.62201 -2.04897

H -3.03014 -3.35694 -1.65474

H -1.5064 -2.43798 -1.73694

C -1.55404 1.24906 -3.01954

H -0.48061 1.27638 -2.82083

H -1.78423 1.89063 -3.88832

H -1.83108 0.21978 -3.26605

C -1.85054 3.03319 -1.44563

H -2.31745 3.33810 -0.50587

H -2.12546 3.76972 -2.22161

H -0.76869 3.04341 -1.29953

C -0.92281 2.09783 2.63096

H 0.04776 1.59787 2.58267

H -1.04663 2.57012 3.62129

H -0.91637 2.87673 1.86537

C -1.98976 0.07218 3.35074

H -2.19987 0.45830 4.36407

H -1.01332 -0.41424 3.36057

H -2.73812 -0.68732 3.11515

C 2.01895 -1.88695 -0.91015

C 1.62248 -3.14279 -0.43483

H 1.83017 -3.42298 0.59161

C 0.97054 -4.06423 -1.26161

H 0.68425 -5.03331 -0.86277

C 0.68749 -3.74141 -2.58648

H 0.18352 -4.45486 -3.23167

C 1.06482 -2.48633 -3.07557

H 0.85798 -2.22027 -4.10855

C 1.72082 -1.57818 -2.24933

H 2.02188 -0.61274 -2.64082

C 2.85383 -0.91548 -0.09709

H 3.89094 -1.05139 -0.44117

C 4.23434 -2.72550 1.98341

H 3.79111 -3.65435 1.61260

H 4.48652 -2.87452 3.03957

H 5.17148 -2.56838 1.43822

C 1.52497 -1.49828 2.81393

H 1.16417 -0.52735 3.16941

H 1.74174 -2.11989 3.68940

H 0.72053 -1.97176 2.24424

C 3.98612 0.27373 2.50881

H 4.87876 0.51944 1.92824

H 4.27858 0.08225 3.54761

H 3.33381 1.15098 2.48799

C 2.58447 0.56997 -0.37442

S 0.89460 1.10770 -0.33670

N 3.65455 1.25582 -0.57719

C 3.71894 2.71066 -0.78265

C 3.00515 3.12633 -2.08223

H 1.93585 2.92214 -2.01553

H 3.15548 4.19480 -2.27588

H 3.41486 2.56480 -2.92882

C 5.21857 3.03140 -0.92143

H 5.38228 4.10330 -1.08054

H 5.75543 2.72714 -0.01718

H 5.64813 2.48247 -1.76524

C 3.16415 3.49541 0.42147

H 3.29868 4.57220 0.26558

H 2.10351 3.28532 0.56580

H 3.70307 3.21524 1.33231

**References**

1. (a) N. Davison, C. L. McMullin, L. Zhang, S. X. Hu, P. G. Waddell, C. Wills, C. Dixon, and E. Lu, “Li vs Na: Divergent Reaction Patterns between Organolithium and Organosodium Complexes and Ligand-Catalyzed Ketone/Aldehyde Methylenation,” *Journal of the American Chemical Society* **145** (2023): 6562–6576. (b) J. Barker, N. Davison, P. G. Waddell, and E. Lu, “Monomeric Lithium and Sodium Silylbenzyl Complexes: Syntheses, Structures, and C=O Bond Olefination,” *Chemical Communications* **59** (2023): 8083–8086. [↑](#endnote-ref-1)
2. R. Fuks, D. Baudoux, C. Piccinni-Leopardi, J. P. Declercq, and M. Van Meerssche, “A New and Facile Synthesis of Ketene Imines and Their 2-Iminoazetidine Dimer from Nitriles via Their Nitrilium Salts,” *The Journal of Organic Chemistry* **53** (1988): 18–22. [↑](#endnote-ref-2)
3. M. Westerhausen, M. Wieneke, B. B. Rademacher, and W. Schwarz, “Synthesis and Characterization of Substituted Benzyl Zinc Derivatives—Molecular Structures of (tmeda)Li–CH(GeMe_3_)Ph, (tmeda)Zn(CH_2_Ph)_2_, (tmeda)Zn[CH(SiMe_3_)Ph]_2_, and (tmeda)Zn[CH(SiMe_3_)Ph]N(H)Si(SiMe_3_)_3_” *Chemische Berichte* **130** (1997): 1499–1505. [↑](#endnote-ref-3)
4. R. C. Clark and J. S. Reid, “The Analytical Calculation of Absorption in Multifaceted Crystals,” *Acta Crystallographica Section A: Foundations of Crystallography* **51** (1995): 887–897. [↑](#endnote-ref-4)
5. CrysAlisPro, Rigaku Oxford Diffraction, Tokyo, Japan. [↑](#endnote-ref-5)
6. O. V. Dolomanov, L. J. Bourhis, R. J. Gildea, J. A. K. Howard, and H. Puschmann, “OLEX2: A Complete Structure Solution, Refinement and Analysis Program,” *Journal of Applied Crystallography* **42** (2009): 339–341. [↑](#endnote-ref-6)
7. G. M. Sheldrick, “SHELXT–Integrated Space-Group and Crystal-Structure Determination,” *Acta Crystallographica Section A: Foundations and Advances* **71** (2015): 3–8. [↑](#endnote-ref-7)
8. L. J. Bourhis, O. V. Dolomanov, R. J. Gildea, J. A. Howard, and H. Puschmann, “The Anatomy of a Comprehensive Constrained, Restrained Refinement Program for the Modern Computing Environment–Olex2 Dissected,” *Acta Crystallographica Section A: Foundations and Advances* **71** (2015): 59–75. [↑](#endnote-ref-8)
9. (a) G. M. Sheldrick, “A Short History of SHELX,” *Acta Crystallographica Section A: Foundations of Crystallography* **64** (2008): 112–122. (b) G. M. Sheldrick, “Crystal Structure Refinement with SHELXL,” *Acta Crystallographica Section C: Structural Chemistry* **71** (2015): 3–8. [↑](#endnote-ref-9)
10. A. D. Becke, “Density-Functional Thermochemistry. III. The Role of Exact Exchange,” *The Journal of Chemical Physics* **98** (1993): 5648–5652. [↑](#endnote-ref-10)
11. C. Lee, W. Yang, and R. G. Parr, “Development of the Colle–Salvetti Correlation-Energy Formula into a Functional of the Electron Density,” *Physical Review B* **37** (1988): 785–789. [↑](#endnote-ref-11)
12. S. Maeda, K. Ohno, and K. Morokuma, “Systematic Exploration of the Mechanism of Chemical Reactions: The Global Reaction Route Mapping (GRRM) Strategy Using the ADDF and AFIR Methods,” *Physical Chemistry Chemical Physics* **15** (2013): 3683–3701. [↑](#endnote-ref-12)
13. Gaussian 16, Revision C.01, M. J. Frisch, G. W. Trucks, H. B. Schlegel, G. E. Scuseria, M. A. Robb, J. R. Cheeseman, G. Scalmani, V. Barone, G. A. Petersson, H. Nakatsuji, X. Li, M. Caricato, A. V. Marenich, J. Bloino, B. G. Janesko, R. Gomperts, B. Mennucci, H. P. Hratchian, J. V. Ortiz, A. F. Izmaylov, J. L. Sonnenberg, D. Williams-Young, F. Ding, F. Lipparini, F. Egidi, J. Goings, B. Peng, A. Petrone, T. Henderson, D. Ranasinghe, V. G. Zakrzewski, J. Gao, N. Rega, G. Zheng, W. Liang, M. Hada, M. Ehara, K. Toyota, R. Fukuda, J. Hasegawa, M. Ishida, T. Nakajima, Y. Honda, O. Kitao, H. Nakai, T. Vreven, K. Throssell, J. A. Montgomery, Jr., J. E. Peralta, F. Ogliaro, M. J. Bearpark, J. J. Heyd, E. N. Brothers, K. N. Kudin, V. N. Staroverov, T. A. Keith, R. Kobayashi, J. Normand, K. Raghavachari, A. P. Rendell, J. C. Burant, S. S. Iyengar, J. Tomasi, M. Cossi, J. M. Millam, M. Klene, C. Adamo, R. Cammi, J. W. Ochterski, R. L. Martin, K. Morokuma, O. Farkas, J. B. Foresman, and D. J. Fox, Gaussian, Inc., Wallingford CT, **2016**. [↑](#endnote-ref-13)
14. Chemcraft - graphical software for visualization of quantum chemistry computations. Version 1.8, build 682. https://www.chemcraftprog.com [↑](#endnote-ref-14)
15. L. Falivene, R. Credendino, A. Poater, A. Petta, L. Serra, R. Oliva, S. Scarano, and L. Cavallo, “SambVca 2. A Web Tool for Analyzing Catalytic Pockets with Topographic Steric Maps,” *Organometallics* **35** (2016): 2286–2293. [↑](#endnote-ref-15)
