# Supplementary material for: Tandem Reactivity of Metal−Carbon and Carbon−Silicon Bonds in Mononuclear α‐Silyl Organolithium or Organosodium Complexes Towards CO, CO2 and Heteroallenes
Source: Angew Chem Int Ed Engl. 2026 Apr 11;65(21):e8906317. doi: 10.1002/anie.8906317 (PMC13182206; doi:10.1002/anie.8906317)
Supplement: Supplementary file 2 — Supporting File 2: anie72186‐sup‐0002‐CIF.zip. [file ANIE-65-e8906317-s001.zip › anie72186-sup-0002-CIF/10_cifreport.html]

checkCIF/PLATON page 2


# checkCIF (basic structural check) running

---

  
*Checking for embedded fcf data in CIF ...*   
*Found embedded fcf data in CIF. Extracting fcf data from uploaded CIF, please wait* **.** **.** 

# checkCIF/PLATON (basic structural check)

---

Structure factors have been supplied for datablock(s) xy2025-04-39-2

THIS REPORT IS FOR GUIDANCE ONLY. IF USED AS PART OF A REVIEW PROCEDURE FOR PUBLICATION, IT SHOULD NOT REPLACE THE EXPERTISE OF AN EXPERIENCED CRYSTALLOGRAPHIC REFEREE.

```
No syntax errors found. CIF dictionary  
Please wait while processing ....  Interpreting this report
```

Structure factor report  
  

**Datablock: xy2025-04-39-2**


---

|  |  |  |
| --- | --- | --- |
| Bond precision: | C-C = 0.0027 A | Wavelength=1.54184 |

|  |  |  |  |
| --- | --- | --- | --- |
| Cell: | a=9.7589(3) | b=18.6713(6) | c=12.3973(4) |
|  | alpha=90 | beta=90.891(3) | gamma=90 |
| Temperature: | 100 K |  |  |

|  |  |  |
| --- | --- | --- |
|  | Calculated | Reported |
| Volume | 2258.66(12) | 2258.66(12) |
| Space group | P 21/n | P 1 21/n 1 |
| Hall group | -P 2yn | -P 2yn |
| Moiety formula | C15 H39 N4 Na S Si | C15 H39 N4 Na S Si |
| Sum formula | C15 H39 N4 Na S Si | C15 H39 N4 Na S Si |
| Mr | 358.64 | 358.64 |
| Dx,g cm-3 | 1.055 | 1.055 |
| Z | 4 | 4 |
| Mu (mm-1) | 1.974 | 1.974 |
| F000 | 792.0 | 792.0 |
| F000' | 796.31 |  |
| h,k,lmax | 12,23,15 | 12,23,15 |
| Nref | 4792 | 4675 |
| Tmin,Tmax | 0.808,0.871 | 0.622,0.730 |
| Tmin' | 0.805 |  |

|  |  |
| --- | --- |
| Correction method= # Reported T Limits: Tmin=0.622 Tmax=0.730 AbsCorr = GAUSSIAN |  |

|  |  |
| --- | --- |
| Data completeness= 0.976 | Theta(max)= 77.203 |

|  |  |
| --- | --- |
| R(reflections)= 0.0391( 3601) | wR2(reflections)= 0.1046( 4675) |
| |  |  | | --- | --- | | S = 1.033 | Npar= 208 | |

---

```
The following ALERTS were generated. Each ALERT has the format
       test-name_ALERT_alert-type_alert-level.
Click on the hyperlinks for more details of the test.


---

Alert level G
PLAT912_ALERT_4_G Missing # of FCF Reflections Above STh/L=  0.600        117 Note  
PLAT941_ALERT_3_G Average HKL Measurement Multiplicity ...........        2.7 Low   
PLAT969_ALERT_5_G The 'Henn et al.' R-Factor-gap value ...........      2.071 Note  
              Predicted wR2: Based on SigI**2  5.05 or SHELX Weight 10.13       
PLAT978_ALERT_2_G Number C-C Bonds with Positive Residual Density.          2 Info  
PLAT994_ALERT_1_G SHELXL .ins Contains no or MERG 0 Instruction ..          ! Note  


---

   0 ALERT level A = Most likely a serious problem - resolve or explain
   0 ALERT level B = A potentially serious problem, consider carefully
   0 ALERT level C = Check. Ensure it is not caused by an omission or oversight
   5 ALERT level G = General information/check it is not something unexpected

   1 ALERT type 1 CIF construction/syntax error, inconsistent or missing data
   1 ALERT type 2 Indicator that the structure model may be wrong or deficient
   1 ALERT type 3 Indicator that the structure quality may be low
   1 ALERT type 4 Improvement, methodology, query or suggestion
   1 ALERT type 5 Informative message, check
```

---

---

It is advisable to attempt to resolve as many as possible of the alerts in all categories. Often the minor alerts point to easily fixed oversights, errors and omissions in your CIF or refinement strategy, so attention to these fine details can be worthwhile. It is up to the individual to critically assess their own results and, if necessary, seek expert advice. |

---

**PLATON version of 15/01/2026; check.def file version of 02/01/2026**

|  |
| --- |
| **Datablock xy2025-04-39-2** - ellipsoid plot |
|  |

---

 Download CIF editor (publCIF) from the IUCr   
 Download CIF editor (enCIFer) from the CCDC   
 Test a new CIF entry 
