# Supplementary material for: Tandem Reactivity of Metal−Carbon and Carbon−Silicon Bonds in Mononuclear α‐Silyl Organolithium or Organosodium Complexes Towards CO, CO2 and Heteroallenes
Source: Angew Chem Int Ed Engl. 2026 Apr 11;65(21):e8906317. doi: 10.1002/anie.8906317 (PMC13182206; doi:10.1002/anie.8906317)
Supplement: Supplementary file 2 — Supporting File 2: anie72186‐sup‐0002‐CIF.zip. [file ANIE-65-e8906317-s001.zip › anie72186-sup-0002-CIF/fig4.pdf]

(a)

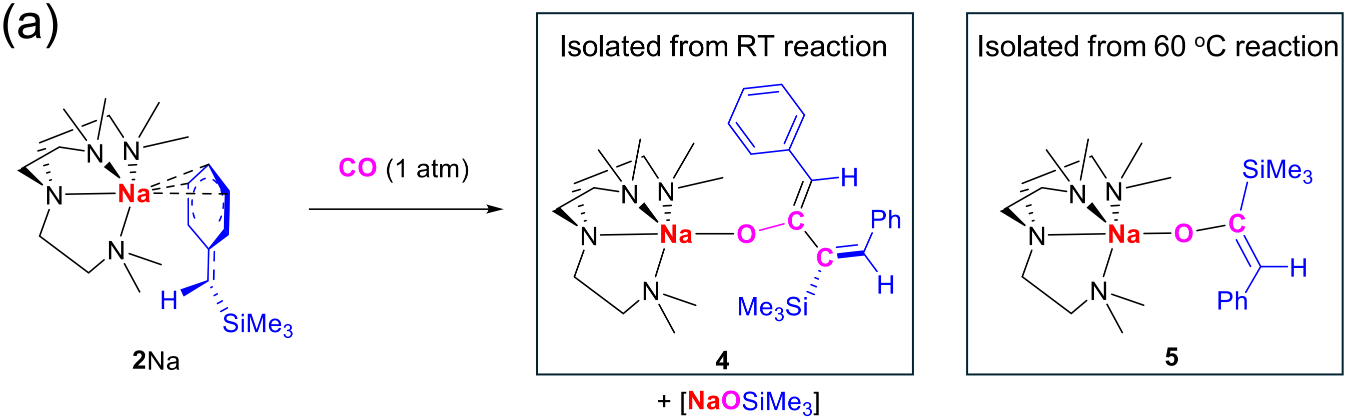

Condition	**4** : **5** ratio in crude product *	Isolated product and yield
RT, 91 h	1 : 1	0.9%, **4**
60 °C, 25 h	1 : 5	20%, **5**

*: There are unidentifiable mixtures in the crude products for both RT and 60 °C reactions.

(b)

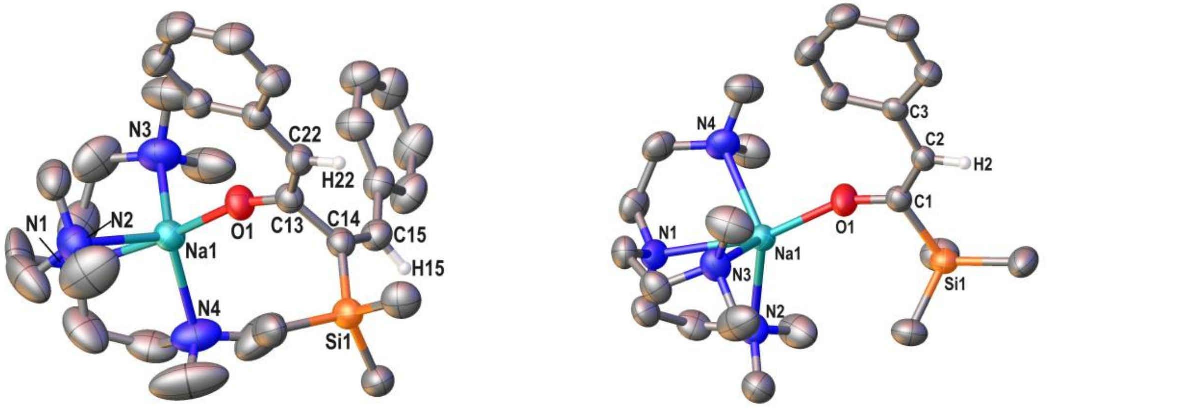

4 **5**
