# Supplementary figures and images for: Tandem Reactivity of Metal−Carbon and Carbon−Silicon Bonds in Mononuclear α‐Silyl Organolithium or Organosodium Complexes Towards CO, CO2 and Heteroallenes
Source: Angew Chem Int Ed Engl. 2026 Apr 11;65(21):e8906317. doi: 10.1002/anie.8906317 (PMC13182206; doi:10.1002/anie.8906317)

(a)

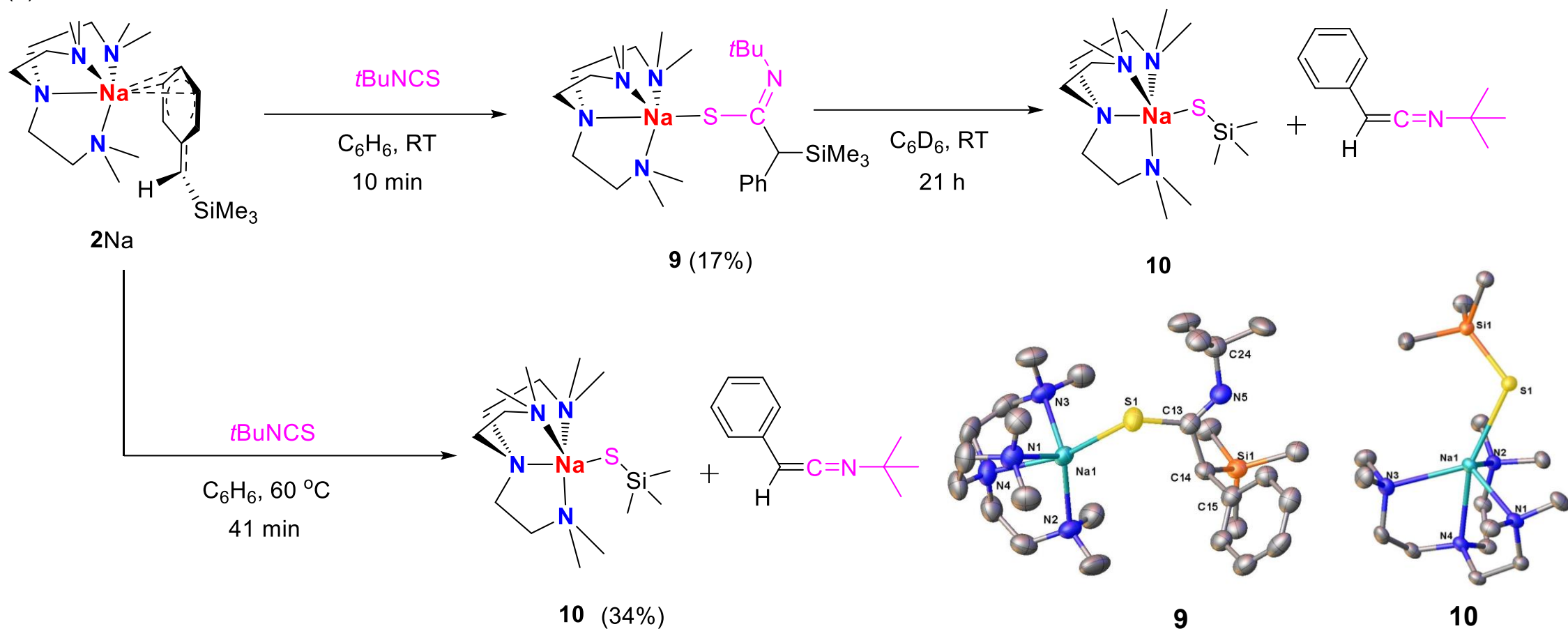

(b)

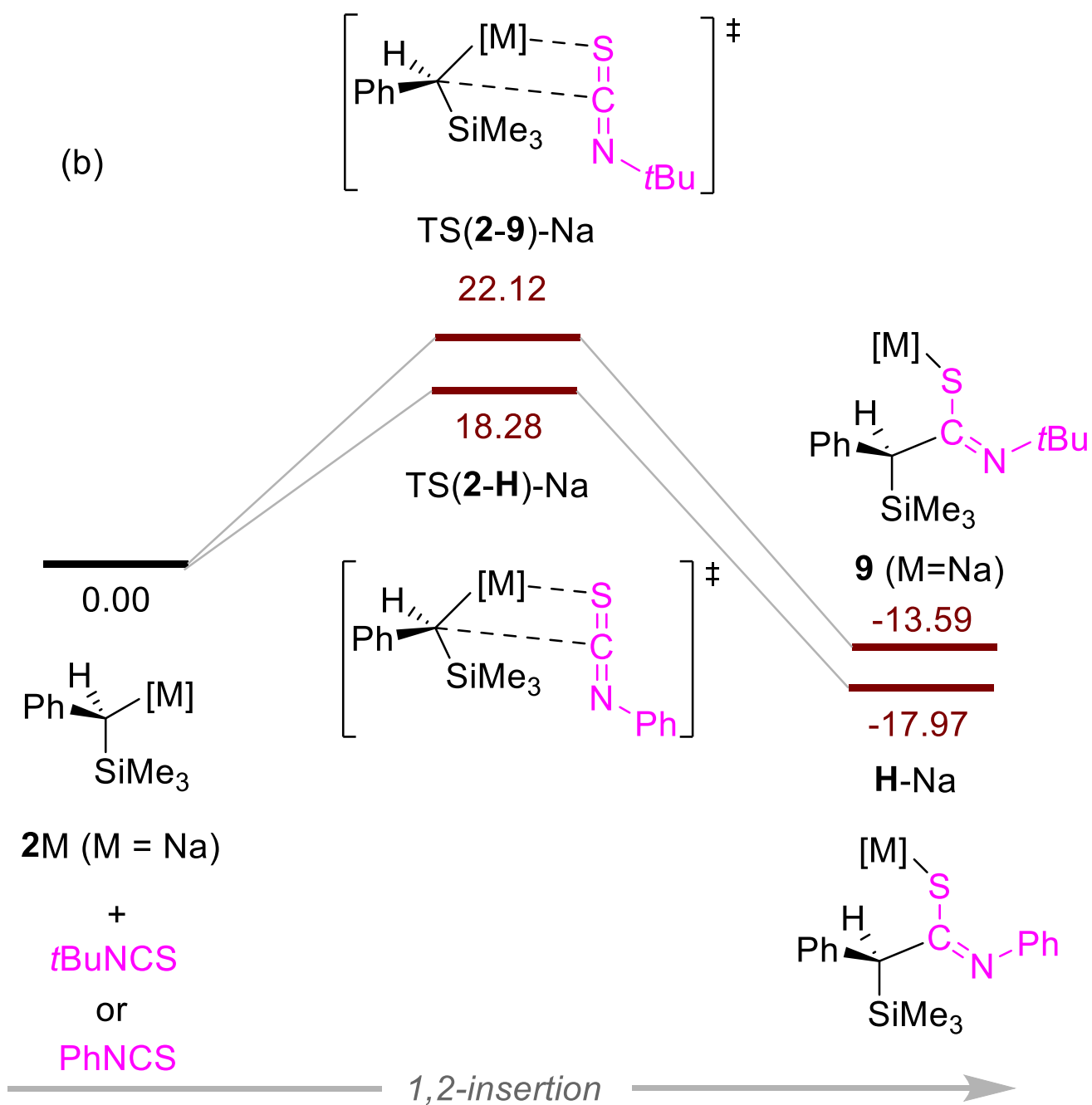pathways of sodium complex **2Na**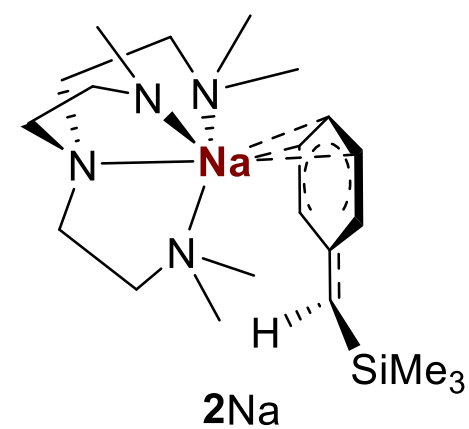

Supplement: Supplementary file 2 — Supporting File 2: anie72186‐sup‐0002‐CIF.zip. [file ANIE-65-e8906317-s001.zip › anie72186-sup-0002-CIF/fig13.pdf]

(a)

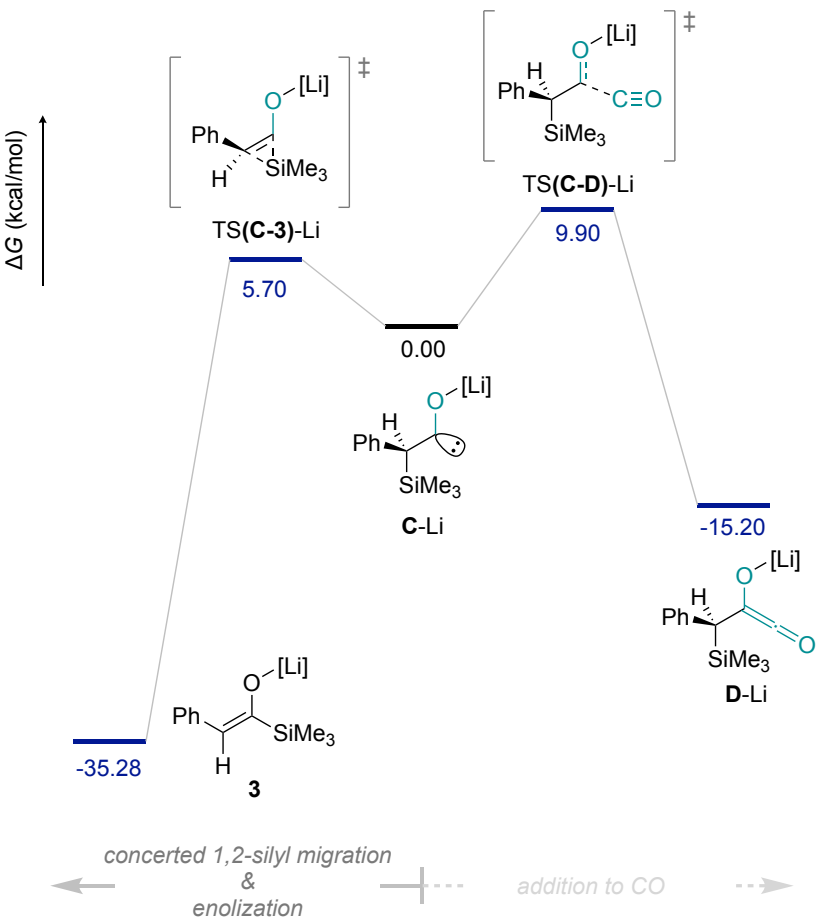

(b)

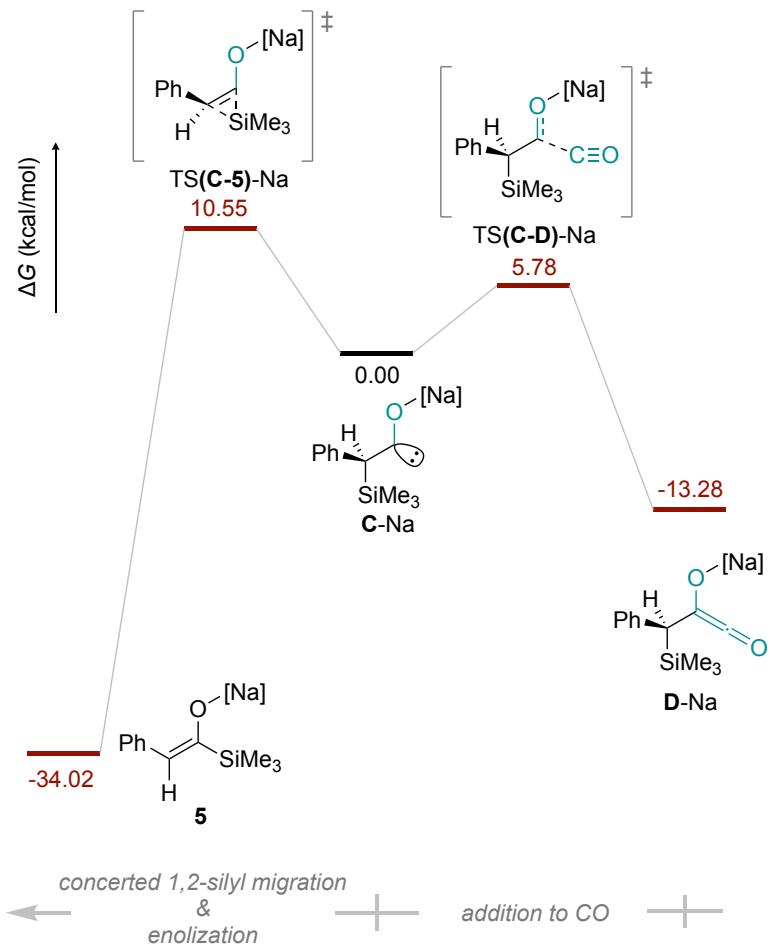

Supplement: Supplementary file 2 — Supporting File 2: anie72186‐sup‐0002‐CIF.zip. [file ANIE-65-e8906317-s001.zip › anie72186-sup-0002-CIF/fig7.pdf]
